# Supplementary material for: A functional analysis of 180 cancer cell lines reveals conserved intrinsic metabolic programs
Source: Mol Syst Biol. 2022 Nov 2;18(11):e11033. doi: 10.15252/msb.202211033 (PMC9627673; doi:10.15252/msb.202211033)
Supplement: Supplementary file 1 — Appendix S1 [file MSB-18-e11033-s006.pdf]

Appendix to:

A functional analysis of 180 cancer cell lines reveals conserved intrinsic metabolic programs

Sarah Cherkaoui [1,2], Stephan Durot [1,2], Jenna Bradley [3], Susan Critchlow [3], Sebastien Dubuis [1], Mauro Miguel Masiero [1,2], Rebekka Wegmann [1,2], Berend Snijder [1], Alaa Othman [1,4], Claus Bendtsen [3], Nicola Zamboni [1, 4]

[1] Institute of Molecular Systems Biology, ETH Zürich, Otto-Stern-Weg 3, 8093 Zürich, Switzerland

[2] PhD Program in Systems Biology, Life Science Zürich, 8057 Zürich, Switzerland

[3] AstraZeneca, R&D, 310 Milton Road, CB4 0WG Cambridge, United Kingdom

[4] PHRT Swiss Multi-OMICS Center / smoc.ethz.ch, Switzerland

Table of Content:

Appendix Figures S1-7

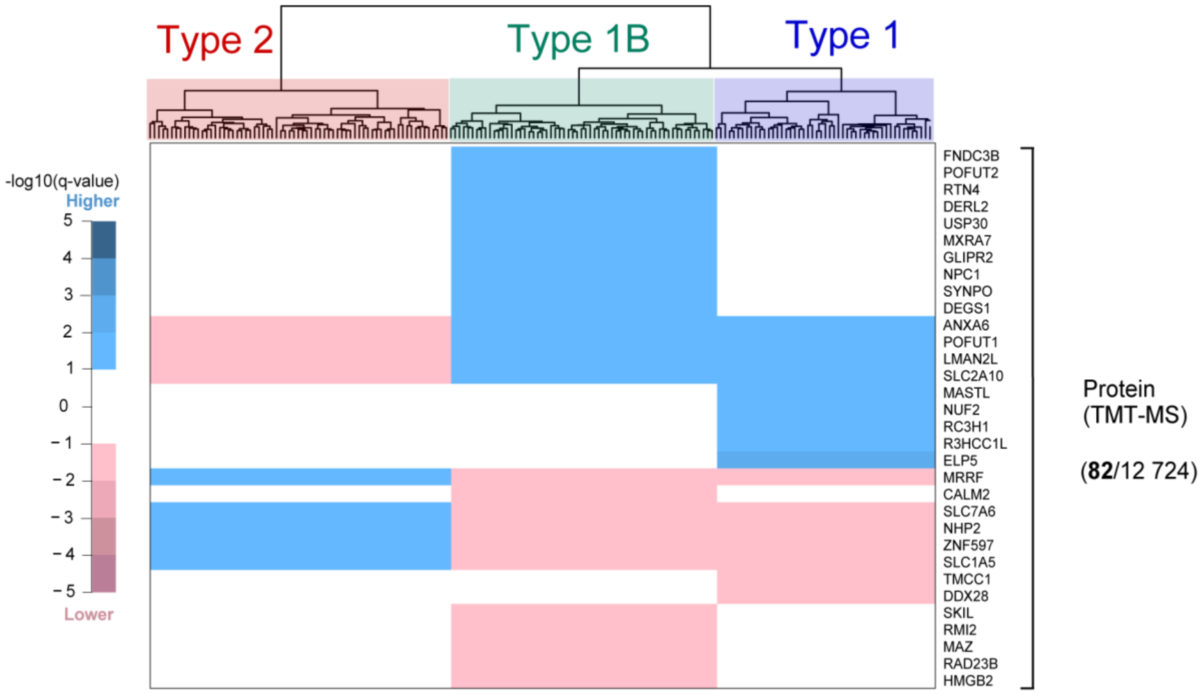

Appendix Figure S1. The proteins associated to metabolic activity phenotypes. Summary of significant associations between metabolic types and proteins levels from Tandem Mass Tag Mass Spectrometry (TMT-MS) dataset. The list and number of traits integrated to the metabolic phenotype are listed on the right panel, where traits were considered significant at 10% FDR (cell lines n=180). For visualization, q-values were extended with a sign to indicate whether the trait is significantly higher or lower in the compared to the rest of the tree. Only traits significantly associated to the tree main types (1, 2, and 1B) are shown. Full results are reported in data availability.

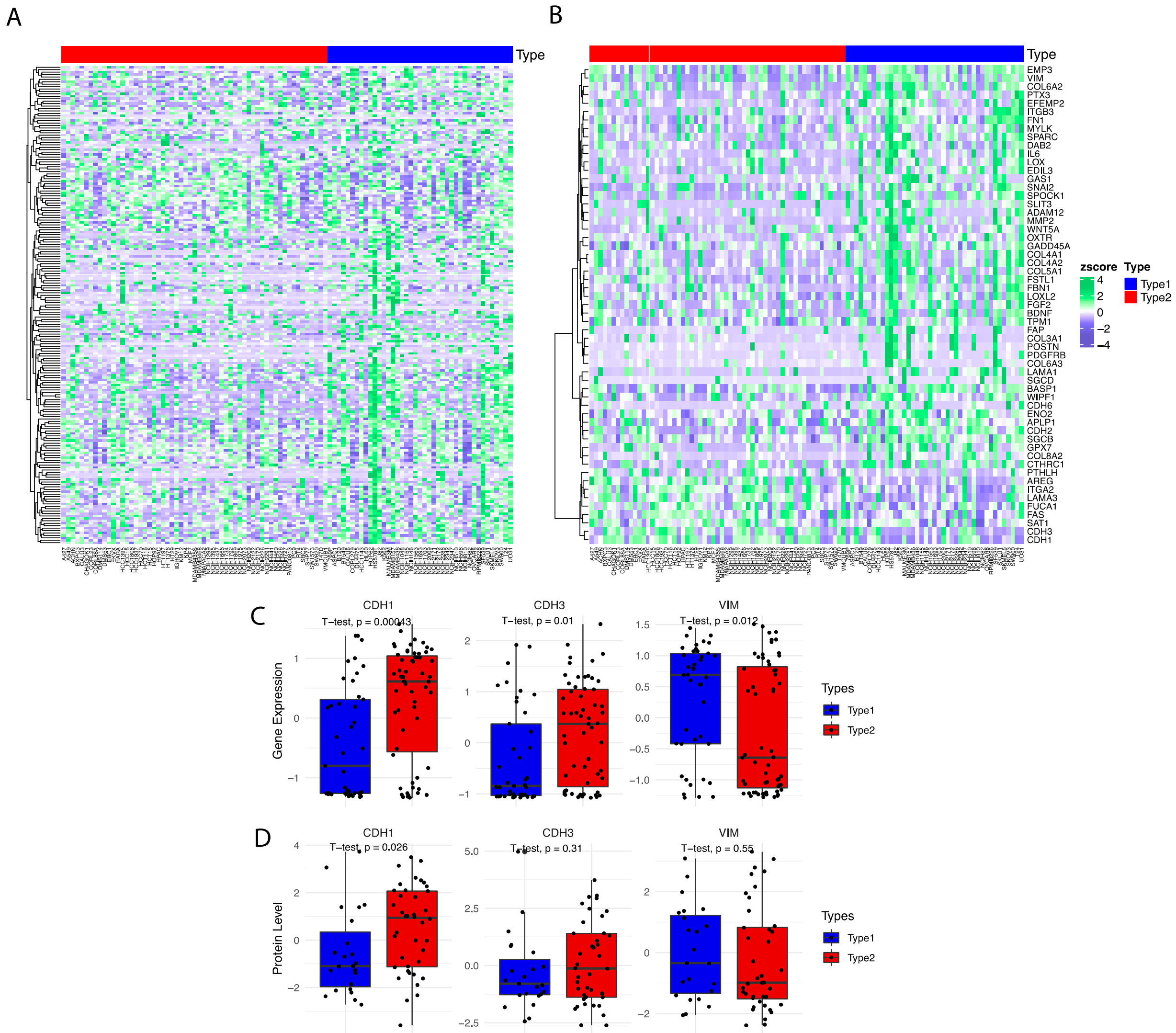

Appendix Figure S2. EMT gene expression signature across types. A. Gene expression EMT in the two metabolic types, where gene set was taken from MSigDB (GSEA). B. Expression of 57 significatively changing EMT genes (out of 197) between type 1 and 2. Comparison of genes expression and protein levels of specific EMT genes, cadherin and vimentin. C. Gene expression data taken from CCLE. D. Protein levels taken from Tandem Mass Tag Mass Spectrometry (TMT-MS) dataset (Nusinow et al.). Data information: Each dot depicts a cell line. Boxplot depicts first quartile, median and third quartile, two-sided unpaired student t-test.

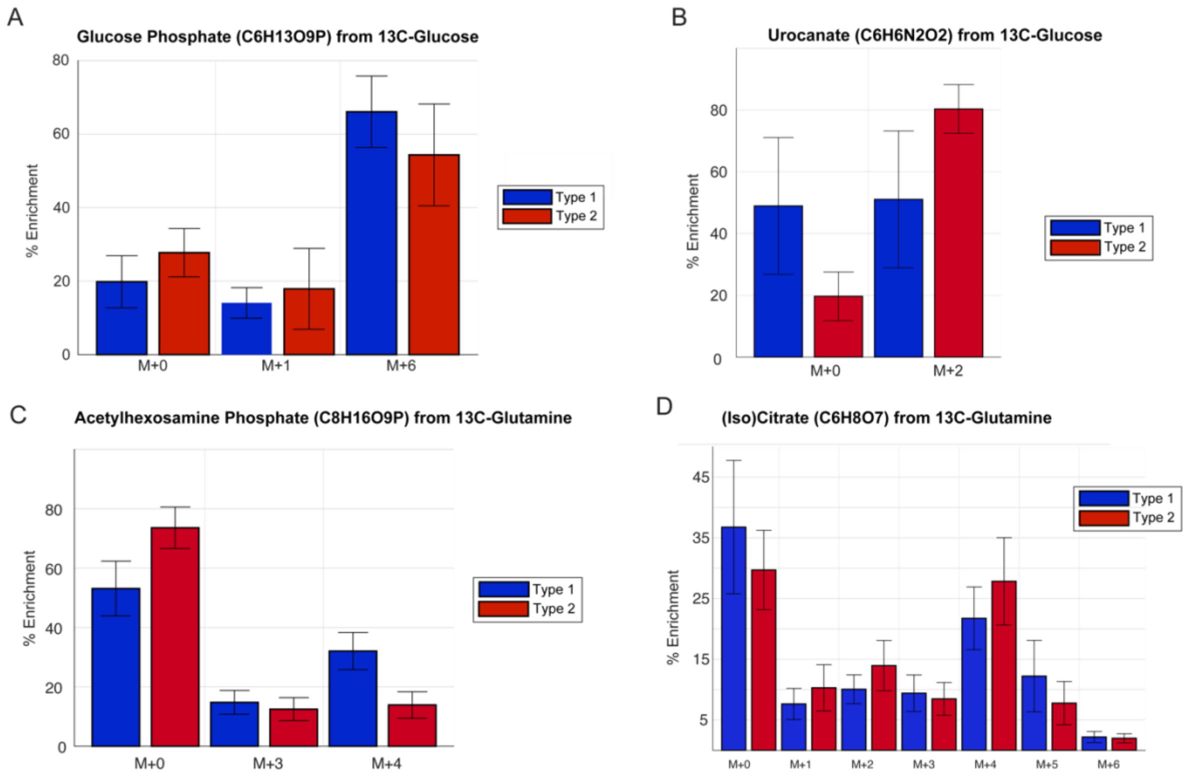

Appendix Figure S3. Mass distribution vector of changing metabolites between metabolic types. A. Glucose phosphate labeled from [U-13C]glucose. B. Urocanate labeled from [U-13C]glucose. C. Acetylhexosamine phosphate labeled from [U-13C]glutamine. D. (Iso)Citrate labeled from [U-13C]glutamine.

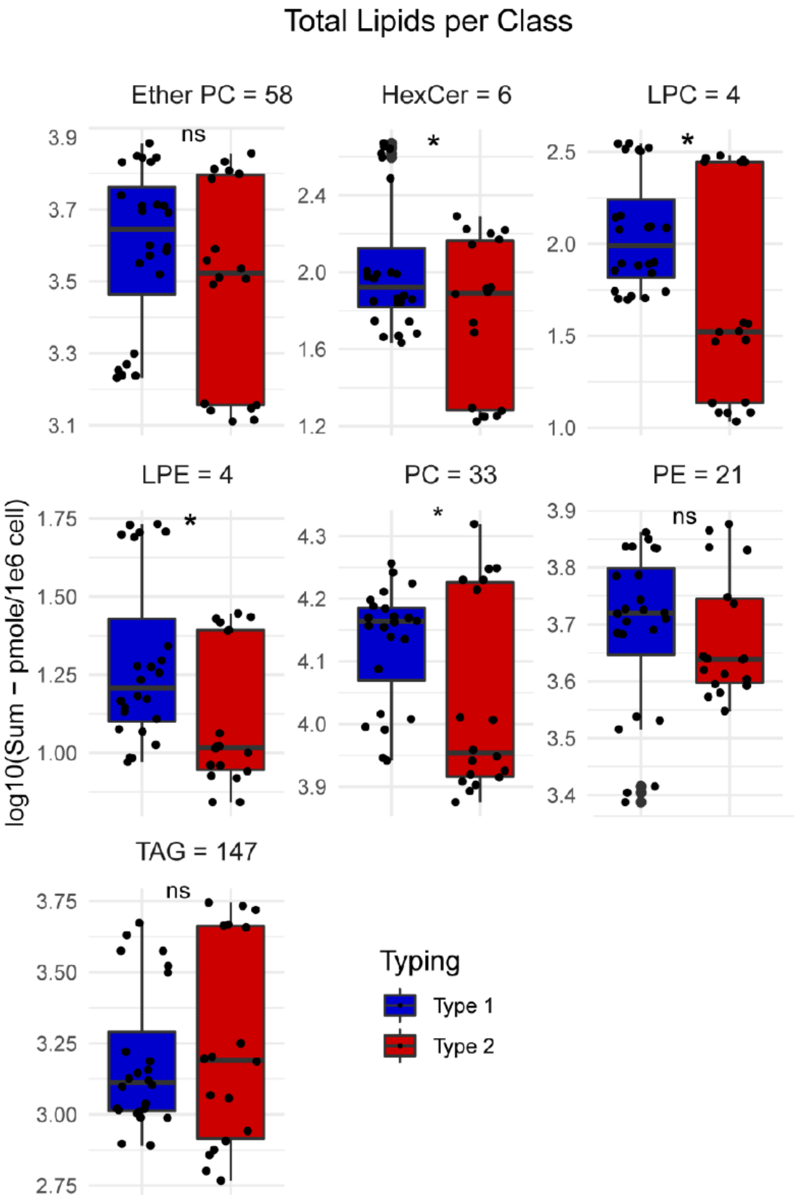

Appendix Figure S4. Differences in lipid content of individual class, with number of lipids per class in title (cell lines n=7, Student t-test). ns: p > 0.05, *: p <= 0.05, **: p <= 0.01, ***: p <= 0.001, ****: p <= 0.0001. Abbreviation: ether phosphatidylcholines (ether PC), hexosyl-ceramide (HexCer), lysophosphatidylcholines (LPC), lysophosphatidylethanolamine (LPE), phosphatidylcholines (PC), phosphatidylethanolamine (PE), sphingomyelin (SM), and triacylglycerols (TAG).

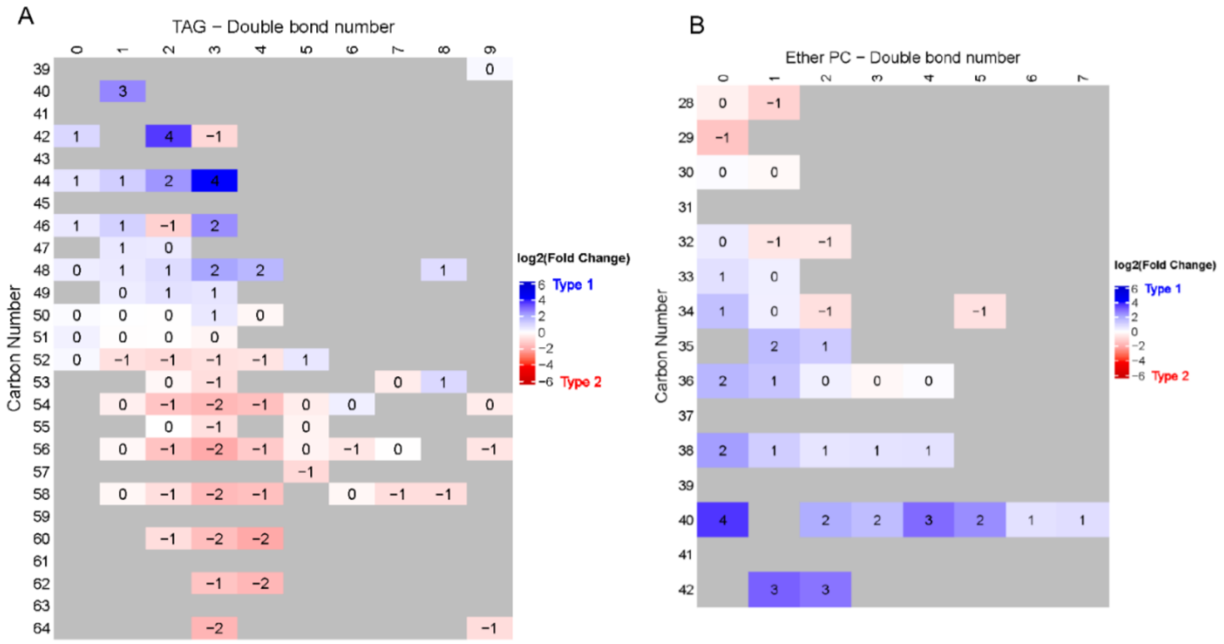

Appendix Figure S5. Comparison of lipid species by double bon and carbon number. Differential analysis of A. TAG and B. Ether PC lipid species displayed per double bond number and carbon number. Abbreviation: triacylglycerols (TAG) and ether phosphatidylcholines (ether PC).

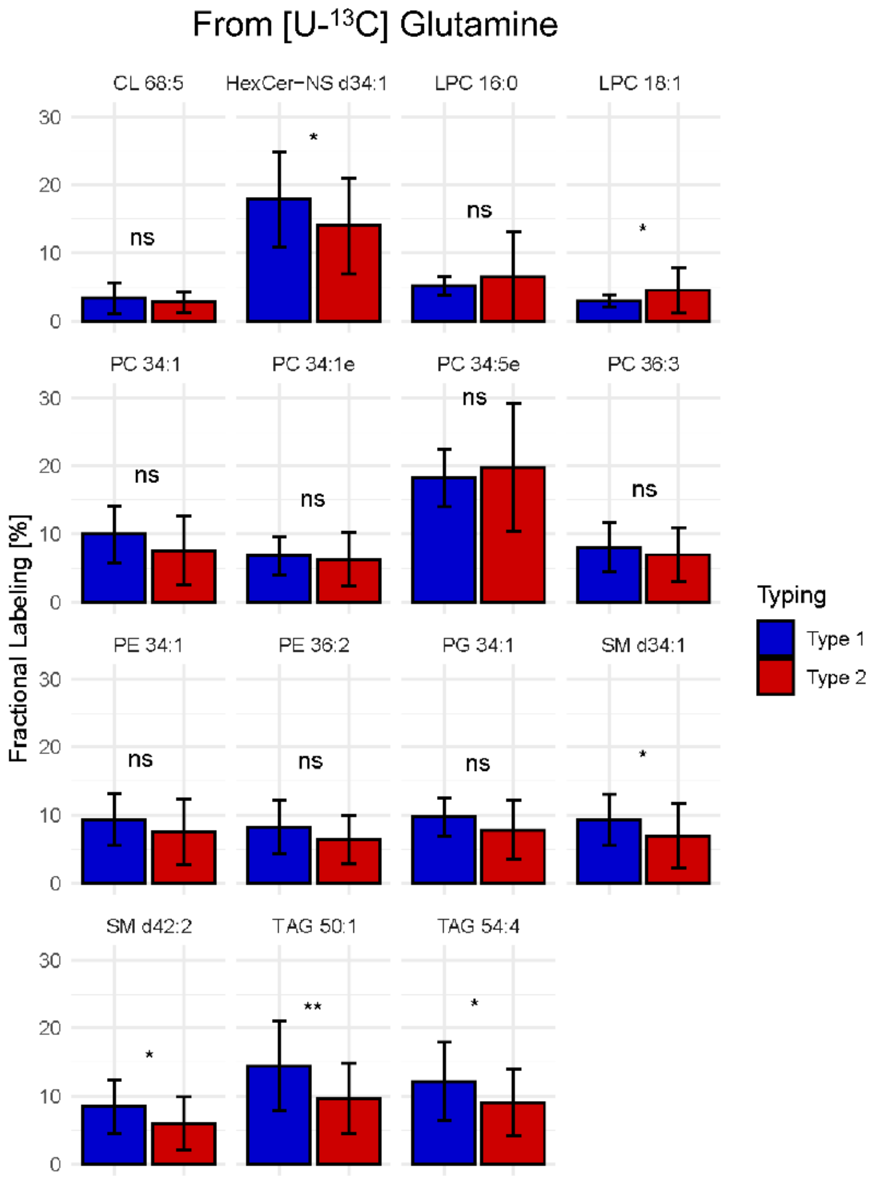

Appendix Figure S6. Differential *de novo* lipid biosynthesis from [U-^{13}C]glutamine. Bar plot (mean ± standard deviation) of fractional labeling contribution from [U-^{13}C]glutamine of the most abundant lipids per lipid class (cell lines n=9, Student t-test). ns: p > 0.05, *: p <= 0.05, **: p <= 0.01, ***: p <= 0.001, ****: p <= 0.0001. Abbreviation: cardiolipins (CL), hexosyl-ceramide (HexCer), lysophosphatidylcholines (LPC), phosphatidylcholines (PC), phosphatidylethanolamine (PE), phosphatidylglycerols (PG), sphingomyelin (SM), and triacylglycerols (TAG).

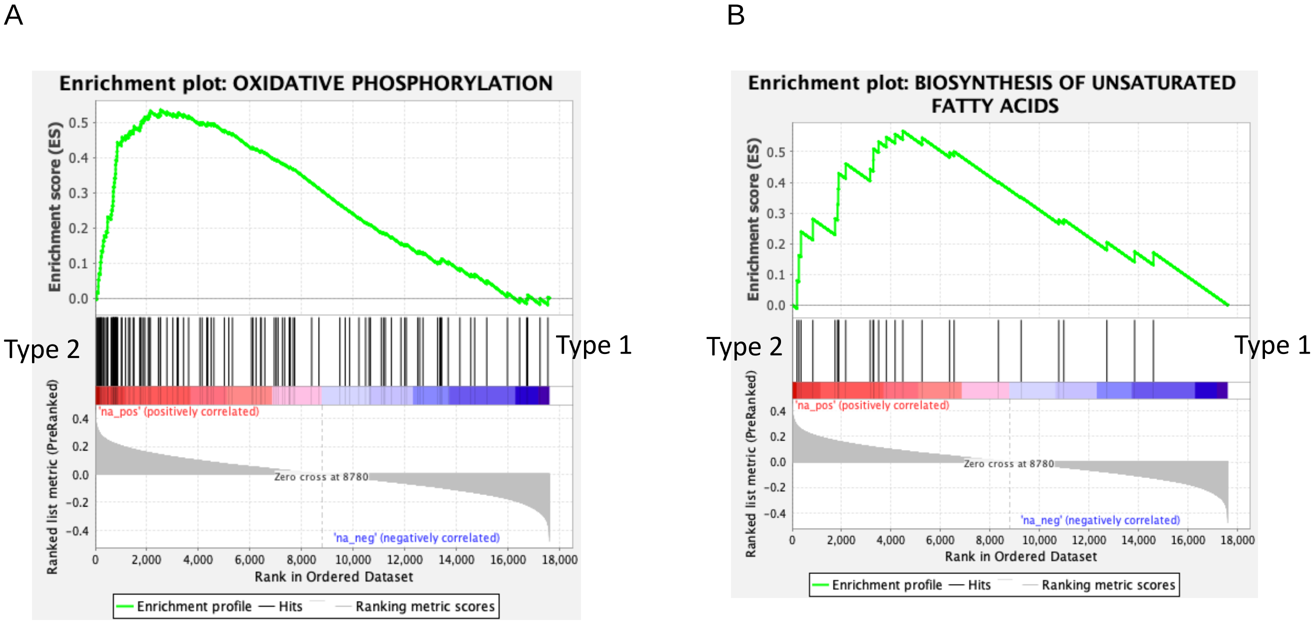

Appendix Figure S7. Pathway dependency of metabolic types. A. Results of gene set enrichment of oxidative phosphorylation and B. biosynthesis of unsaturated fatty acids. Gene set enrichment analysis results and figures were generated using leading edge analysis from GSEA (https://www.gsea-msigdb.org/gsea/index.jsp).
