# Supplementary material for: A functional analysis of 180 cancer cell lines reveals conserved intrinsic metabolic programs
Source: Mol Syst Biol. 2022 Nov 2;18(11):e11033. doi: 10.15252/msb.202211033 (PMC9627673; doi:10.15252/msb.202211033)
Supplement: Supplementary file 2 — Expanded View Figures PDF [file MSB-18-e11033-s007.pdf]

Expanded View Figures

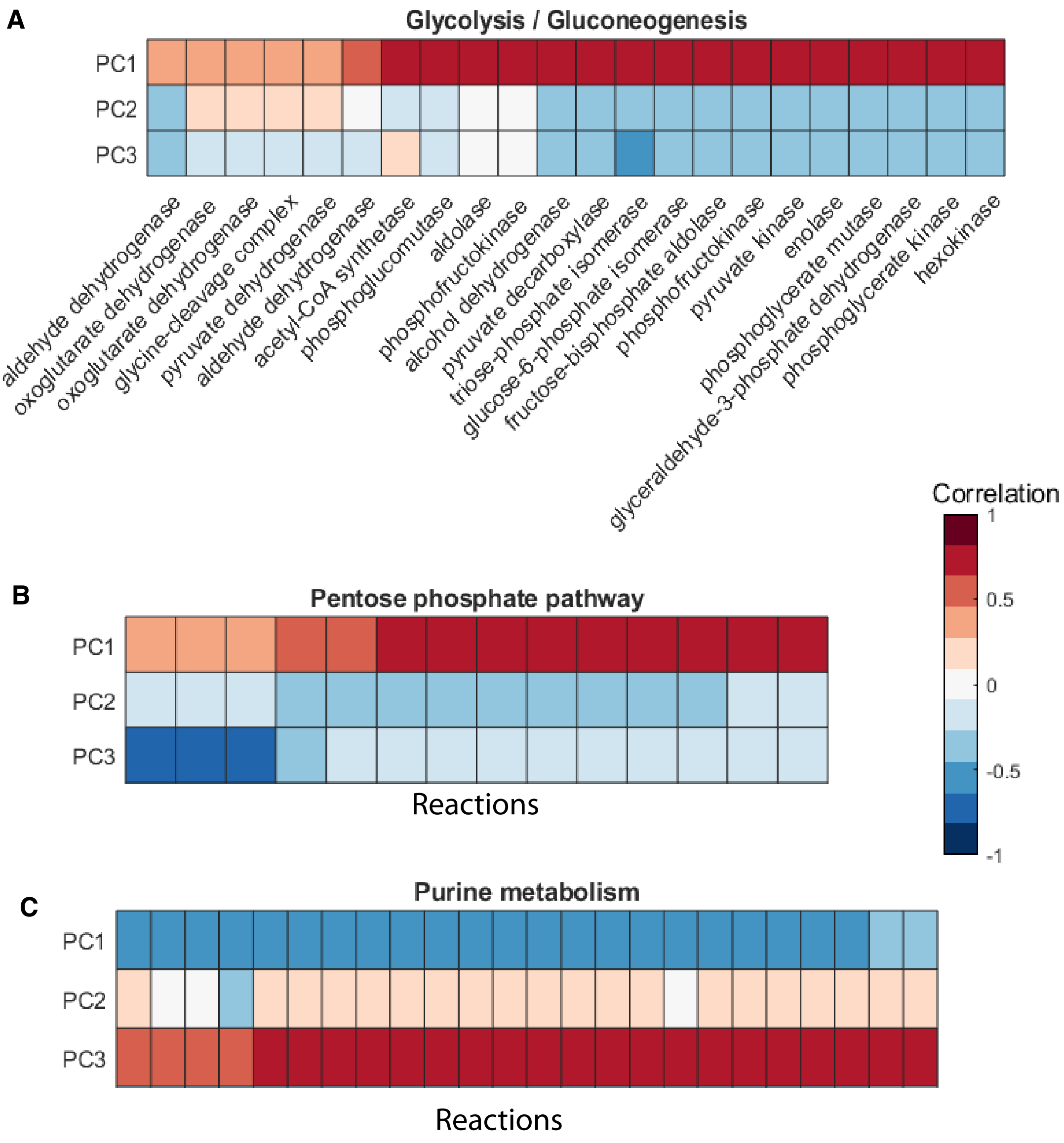

Figure EV1. Pathway activity scoring reveals link between metabolites levels and fluxes.

A–C Pearson correlation between metabolites levels factorized into principal components and fluxes of a pathway. Fluxes and metabolites of (A) glycolysis, (B) pentose phosphate pathway, and (C) purine metabolism. Data taken from Hackett *et al* (2016). PC1 is used as pathway activity score.

© 2022 The Authors

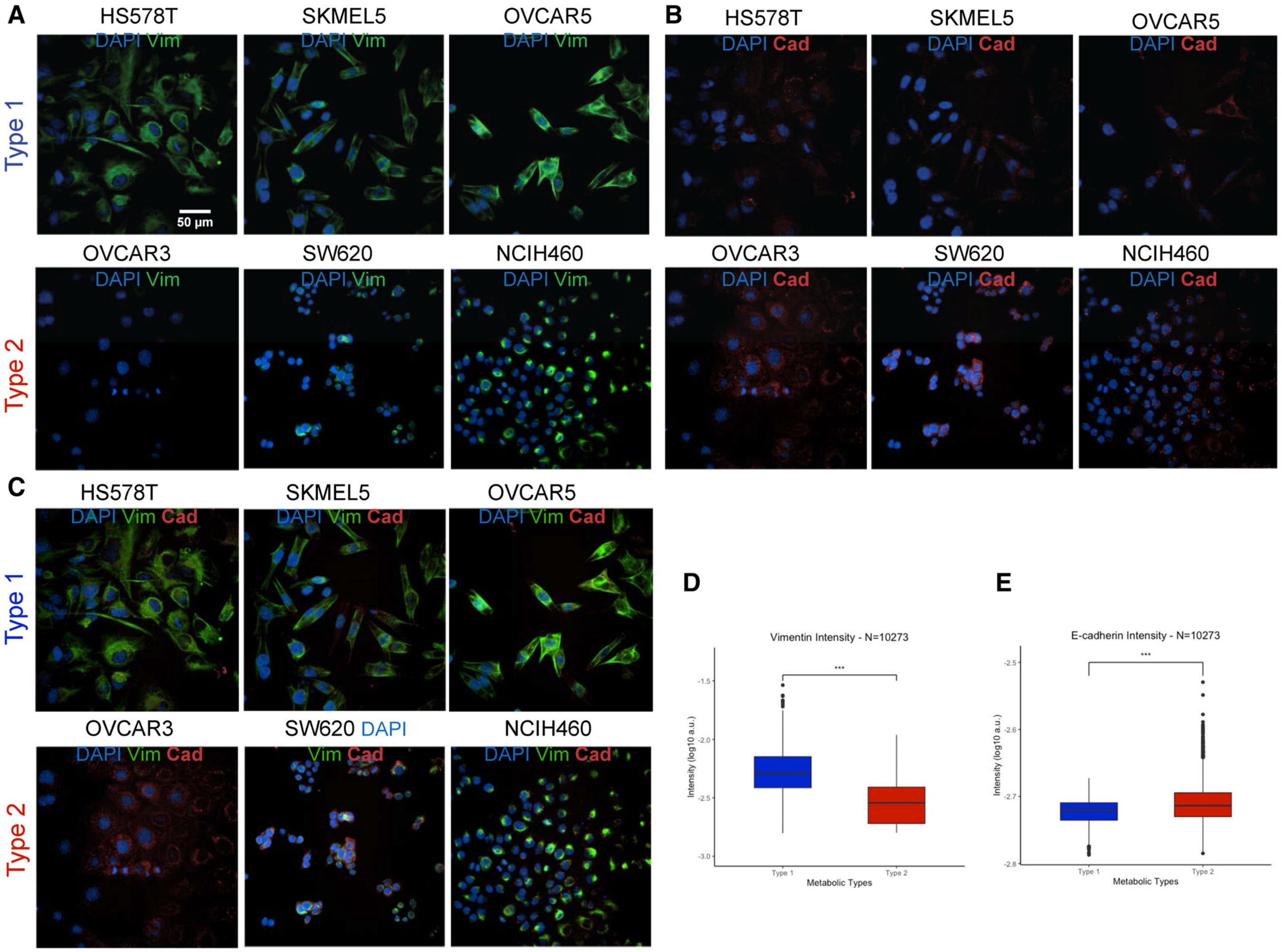

Figure EV2. Relation between metabolic types and epithelial/mesenchymal state.

A–C Metabolic types stained for the nucleus (blue), (A) for vimentin (green), and (B) e-cadherin (red), (C) both, two markers of EMT. Scaling bar in (A) applies to all images.

D, E Quantification of the expression of (D) vimentin and (E) e-cadherin of all segmented cells (number of cells $n = 10,273$, boxplot depicts first quartile, median, and third quartile, two-sided unpaired student *t*-test). $^{***}P \leq 0.001$. Abbreviation: a.u. arbitrary units.

Figure EV3. Difference in lipids unsaturation.

A, B Lipid unsaturation, concentration-weighted average double per acyl chain, across metabolic types (cell lines $n = 7$ with three biological replicates per cell line and two technical replicates, boxplot depicts first quartile, median, and third quartile, two-sided unpaired student *t*-test) for (A) each lipid class and (B) for all lipids. ns: $P > 0.05$, $^{*}P < 0.05$, $^{**}P < 0.01$, $^{***}P < 0.001$, $^{****}P < 0.0001$. Abbreviation: cardiolipins (CL), ether phosphatidylcholines (ether PC), hexosyl-ceramide (HexCer), lysophosphatidylcholines (LPC), lysophosphatidylethanolamine (LPE), phosphatidylethanolamine (PE), phosphatidylserine (PS), phosphatidylcholines (PC), sphingomyelin (SM), and triacylglycerols (TAG).

▶

© 2022 The Authors

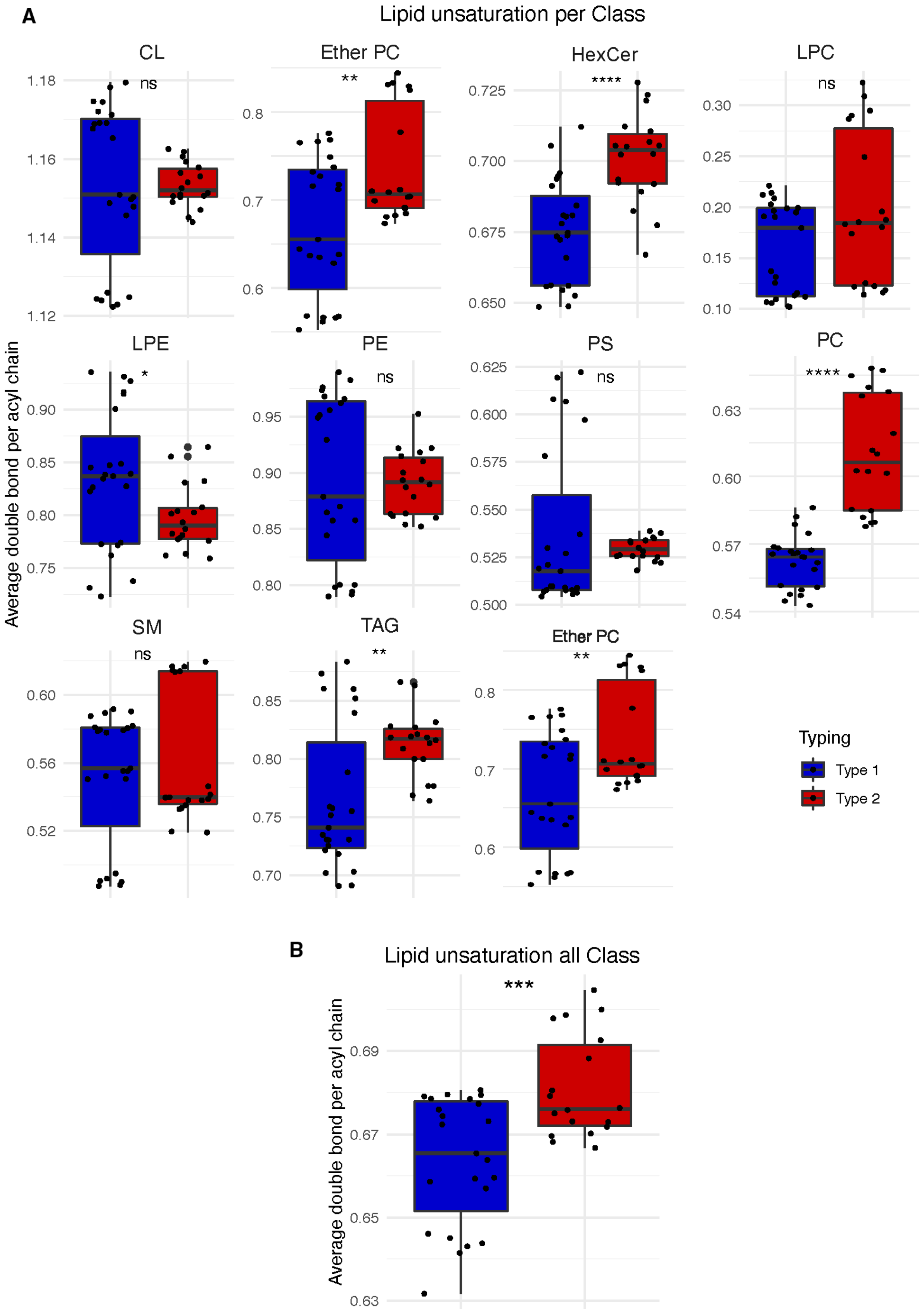

Figure EV3.

 © 2022 The Authors

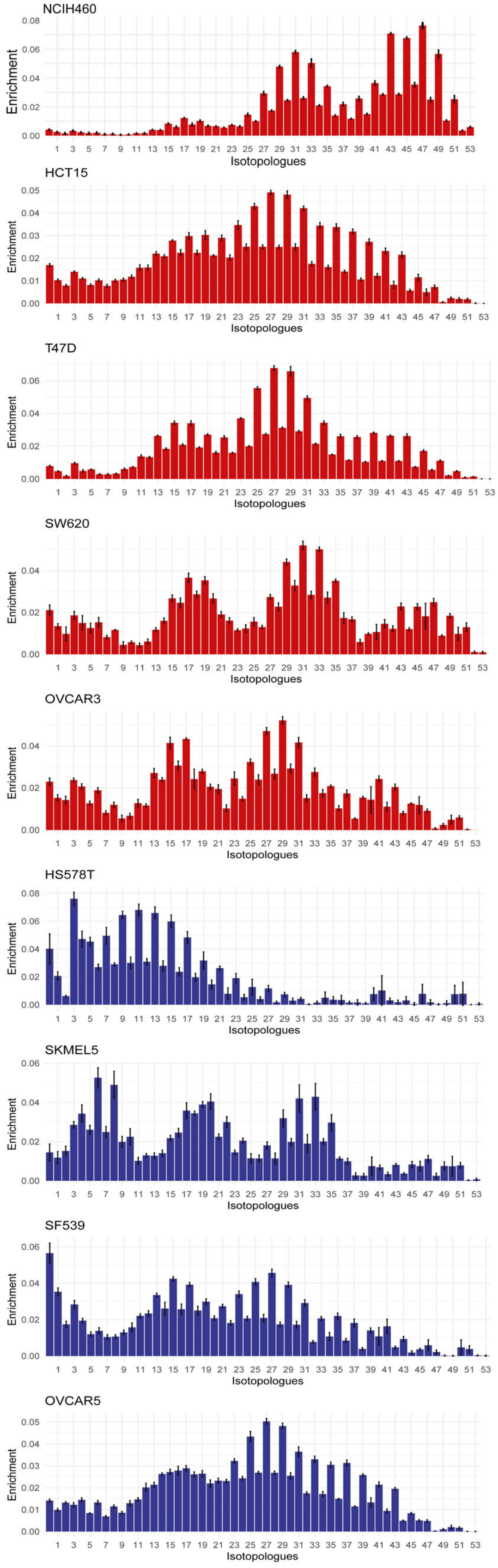

Figure EV4. Mass distribution vector of TAG 50:1 labeled from [U-^{13}C] glucose for all followed up cell lines (cell lines *n* = 9, with three biological replicates per cell line and two technical replicates, mean ± standard deviation).

© 2022 The Authors
