# Supplementary material for: A functional analysis of 180 cancer cell lines reveals conserved intrinsic metabolic programs
Source: Mol Syst Biol. 2022 Nov 2;18(11):e11033. doi: 10.15252/msb.202211033 (PMC9627673; doi:10.15252/msb.202211033)
Supplement: Supplementary file 6 — PDF+ [file MSB-18-e11033-s005.pdf]

Article

TRANSPARENT PROCESS OPEN ACCESS

molecular systems biology

A functional analysis of 180 cancer cell lines reveals conserved intrinsic metabolic programs

Sarah Cherkaoui[1,2], Stephan Durot[1,2], Jenna Bradley[3], Susan Critchlow[3], Sebastien Dubuis[1], Mauro Miguel Masiero[1,2], Rebekka Wegmann[1,2], Berend Snijder[1], Alaa Othman[1,4], Claus Bendtsen[3] & Nicola Zamboni[1,4,*]

Abstract

Cancer cells reprogram their metabolism to support growth and invasion. While previous work has highlighted how single altered reactions and pathways can drive tumorigenesis, it remains unclear how individual changes propagate at the network level and eventually determine global metabolic activity. To characterize the metabolic lifestyle of cancer cells across pathways and genotypes, we profiled the intracellular metabolome of 180 pancancer cell lines grown in identical conditions. For each cell line, we estimated activity for 49 pathways spanning the entirety of the metabolic network. Upon clustering, we discovered a convergence into only two major metabolic types. These were functionally confirmed by ^{13}C-flux analysis, lipidomics, and analysis of sensitivity to perturbations. They revealed that the major differences in cancers are associated with lipid, TCA cycle, and carbohydrate metabolism. Thorough integration of these types with multiomics highlighted little association with genetic alterations but a strong association with markers of epithelial–mesenchymal transition. Our analysis indicates that in absence of variations imposed by the microenvironment, cancer cells adopt distinct metabolic programs which serve as vulnerabilities for therapy.

Keywords cancer metabolism; cell lines; metabolomics; metabolic flux; omics
Subject Categories Cancer; Metabolism
DOI 10.15252/msb.202211033 | Received 17 March 2022 | Revised 3 October 2022 | Accepted 7 October 2022
Mol Syst Biol. (2022) 18: e11033

Introduction

Over the past two decades, altered metabolism has re-emerged as a prominent hallmark of cancer (Ward & Thompson, 2012; Pavlova *et al*, 2016)'. Beyond the seminal example of aerobic glycolysis (Warburg & Minami, 1923), multiple examples of dysregulated pathways and novel essential reactions have been presented (Dang *et al*, 2009; Jain *et al*, 2012; Son *et al*, 2013) and gave rise to tailored therapeutic opportunities. A key lesson in oncology that also extends to metabolism is that tumors are heterogeneous and, therefore, their sensitivities to drug or genetic treatments can differ greatly. In the context of metabolism, a main driver of heterogeneity is the tumor microenvironment. Previous studies have demonstrated the relevance of oxygenation and cancer specific nutrient utilization (DeBerardinis *et al*, 2007; Elia & Haigis, 2021), which give cancer cells a unique growth advantage. The second, intrinsic driver of heterogeneity is the genetic makeup of tumor cells, which varies between and within tumors. Mutations in coding sequences or regulatory regions and alterations in copy number may affect gene expression and the activity of proteins and, hence, enzymes. Mutations result in granular differences in pathway utilization, some of which provide a fitness advantage for tumor growth.

Even though intrinsic factors are rooted in genetic variations, genomics is poorly suited to investigate the metabolic heterogeneity of tumor cells. Beyond specific alterations that are frequently recurring in some cancer types (e.g., IDH1 (Dang *et al*, 2009) or PKM2 (Dayton *et al*, 2016)), sequencing of DNA or RNA fails to provide an integrated understanding of pathway activity and carbon fluxes. The latter are hard to predict because they are an emerging property governed also by nutrient availability (i.e., the microenvironment) and allosteric regulation, both of which are not captured by genomics and transcriptomics. Among the arsenal of omics technologies that are available to investigate the molecular underpinnings of cancer cells (reviewed in Li *et al*, 2019b), metabolomics is the ideal approach to assess metabolism in action. In part, this is because regardless of the cause, intrinsic or extrinsic, changes in fluxes are associated to changes in the level of intermediates of the affected pathways.

The power of metabolomics in unraveling metabolic peculiarities of cancer cells is neatly demonstrated by previous milestone studies. For instance, Jain *et al* (2012) highlighted heterogeneity in metabolite uptake and secretion rates and revealed the role of glycine in tumor proliferation. Another example by Chen *et al* (2019) combined ^{13}C tracing and metabolomics to reveal the relation

1 Institute of Molecular Systems Biology, ETH Zürich, Zürich, Switzerland
2 PhD Program in Systems Biology, Life Science Zürich, Zürich, Switzerland
3 AstraZeneca, R&D, Cambridge, UK
4 PHRT Swiss Multi-OMICS Center / smoc.ethz.ch, Zürich, Switzerland
*Corresponding author. Tel: +41 44 633 31 41; E-mail: zamboni@imsb.biol.ethz.ch

© 2022 The Authors. Published under the terms of the CC BY 4.0 license

between central carbon metabolism reprogramming and oncogenic drivers in lung cancers. More recently, Li *et al* (2019a) investigated the relation between individual metabolites and genetic alterations. The authors have identified an association between asparaginase's hypermethylation and asparagine, which serves as a potential therapy selection for specific tumors. Overall, metabolomics has been to use to unravel single metabolites or pathways dysregulation in cancers. However, no systematic characterization of metabolic-wide pathway activity has been accessed to date, thus, leaving some unexplored understanding, like identifying which parts of the network are conjointly reprogrammed.

Here, we leveraged the broad scope of untargeted metabolomics to characterize the intrinsic metabolic heterogeneity of a panel of 180 cancer cell lines across the full metabolic network. To exclude environmental factors, we grew the cancer cell lines in the same medium. We profiled ca. 1,800 putative deprotonated metabolites across the full panel. To gain a global view of metabolic reprogramming, we estimated activity scores for 49 metabolic pathways using factorization by principal components. In spite of the genetic variety of the tested 180 cell lines, we show that they cluster into two metabolic types, which we validated using lipidomics, ^{13}C tracing, and pathway sensitivity analysis. Integration of the metabolic types with genomic and phenotypic data revealed a strong connection with EMT status, which emerges as a main determinant of intrinsic metabolic activity.

Results

Large-scale metabolic profiling and analysis of cancer cell lines

We set out to broadly characterize the metabolic diversity of cancer cell lines by untargeted metabolomics (Fig 1A). To gain a representative dataset, we selected a large panel of 180 cancer cell lines encompassing 11 different lineages (Fig 1B). Our panel overlapped substantially with other major cell line panels, such as the CCLE (Ghandi *et al*, 2019), COSMIC (Forbes *et al*, 2015), and the NCI60 (Shoemaker, 2006; Fig 1C). As our objective was to focus on intrinsic heterogeneity and not be affected by differences in environment (Lagziel *et al*, 2020), we cultured the cell lines in the utmost comparable condition. We grew cells in the same nutrient condition and extracted at comparable confluency and during exponential growth. Sample generation were organized in seven major batches, with two cell lines (MCF7 and MDBADM231) included in all batches. The study design included six measurements per cell line, assessed by untargeted metabolomics by flow injection, high-resolution mass spectrometry (Fuhrer *et al*, 2011; see details in Materials and Methods). Upon data processing and quality control, the resulting metabolomic dataset included 1,809 ions putatively associated to deprotonated metabolites for a total of 1,195 measurements.

The resulting data were subject to an in-depth reproducibility analysis based on the repeated injections of MCF7 and MDBADM231 across the dataset. We used multiple quality metrics calculated for these control cell lines to measure the effect of multiple, state-of-the-art normalization procedures (see details in Materials and Methods). We identified the combination of quantile normalization and ComBat (Johnson *et al*, 2007) as the best option to correct for sample-to-sample differences and batch biases, respectively (Table EV1).

Inference of metabolic phenotypes

A grand challenge in analyzing metabolomics data is interpreting the cause of metabolite changes. For instance, the increase of an individual metabolite could point both to an increase of pathway flux as well as a block of the pathway immediately downstream. To distinguish between these opposite cases, it is necessary to analyze all detectable intermediates of a pathway together. In practice, when the flux of a pathway changes, a shift with coherent sign is observed for most intermediates in the pathway. This is a consequence of enzyme kinetics and the fact that enzymes normally operate close to their substrate affinity (e.g., the Michaelis–Menten affinity constant K_M) and far from saturation (Bar-Even *et al*, 2011; Park *et al*, 2016). Therefore, flux changes cause mild but ubiquitous effects in metabolites. To capture such flux-relevant effects, we devised a strategy that uses principal component analysis to identify common trends across all detectable intermediates of a given pathway (see details in Materials and Methods). A similar concept was successfully applied to gene expression data (Segura-Lepe *et al*, 2019), and we demonstrate that it holds for metabolic systems with exemplary data set (Hackett *et al*, 2016; Fig EV1). Using curated metabolic pathways definition, we projected all cell lines on the first principal component, which provides a qualitative proxy for pathway activity, termed "pathway score." Out of the 1,809 putatively annotated ions, 367 could be mapped to KEGG metabolic pathways. Based on this subset, we could infer the pathway score for 49 metabolic pathways.

To gain a top-down view of the differences in pathways scores across cancer cell lines, we applied hierarchical clustering (Fig 2). Surprisingly, only two major clusters of cell lines emerged, which we refer to as metabolic types. The first cluster (left) was characterized with generally high activity scores for most of the pathways of central carbon metabolism, for example, carbohydrate metabolism, amino acid metabolism, and nucleic acid metabolism. The second cluster (right) was associated with high activity scores in fewer pathways, which includes part of lipid metabolism pathways and cofactor and vitamin metabolism.

Association of multiomics to metabolic types

Identifying only two major clusters across a panel of 180 cancer cell lines was a surprise as it indicates convergence into a few, robust metabolic program. Following this clustering, we performed an in-depth association analysis to find whether one of the branches of the hierarchical clustering tree was associated with any property or trait of the cell lines (e.g., tissue and batch, Fig 2). Tested traits included metadata related to sample collection, histologic properties, genomics, transcriptomics, etc. (Table EV2). We assembled the information of 60,328 traits and for each calculated associations to all tree branches with at least 18 cell lines. This resulted in 1,025,576 *P*-values that accounted for false discovery rate (see Materials and Methods for details). By this procedure, we sought to identify the most significant associations in the clustering tree. In total, we identified 856 significant associations between a trait and a branch of the clustering tree derived from pathway activity scores (Fig 3 and Table EV2).

© 2022 The Authors

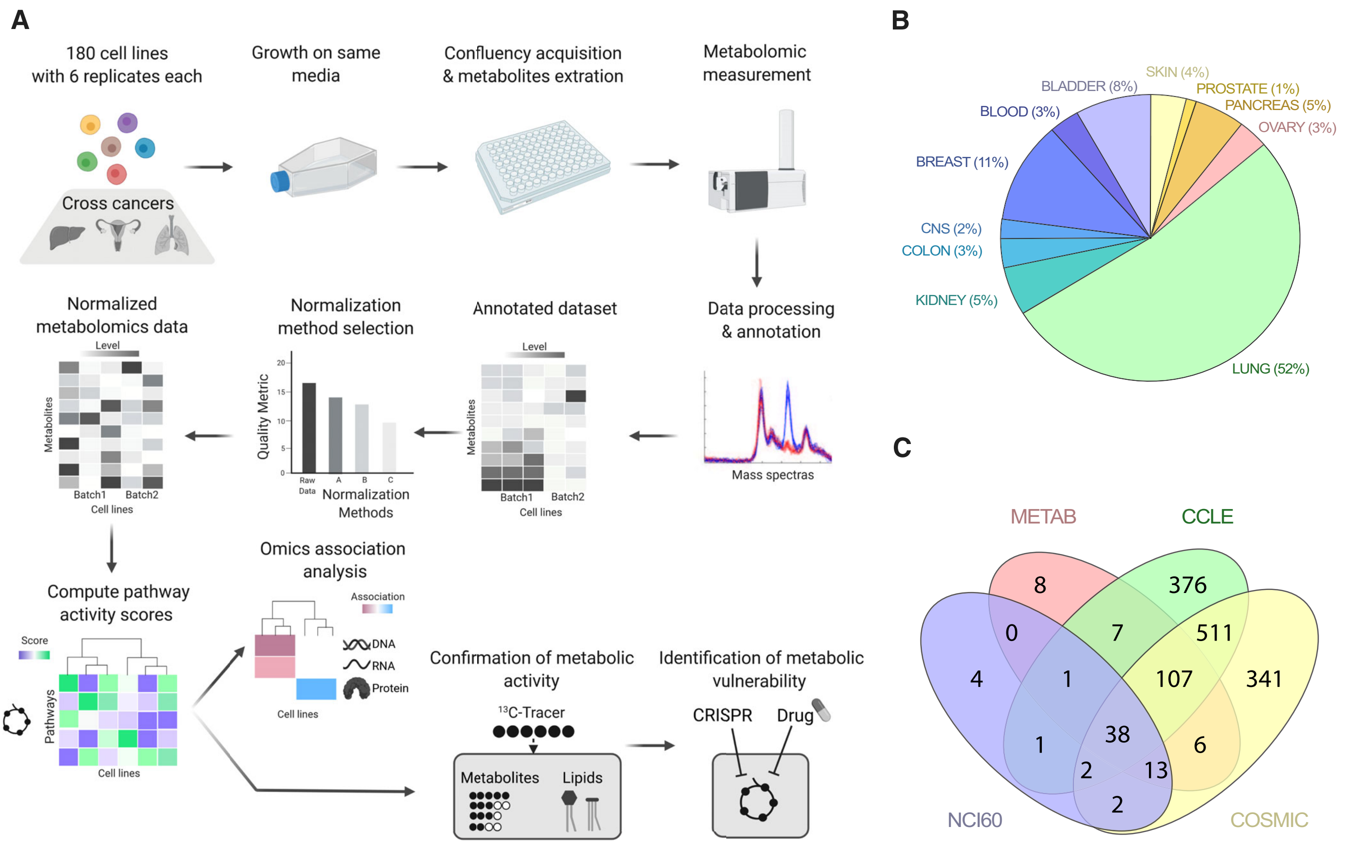

Figure 1. Metabolic profiling of 180 cancer cell lines.

A Schematic summarizing the workflow developed for the comparison of cancer cell lines metabolite profiles.

B 180 cancer cell lines from more than 11 tissues of origin were profiled.

C Overlap of this study cell line panel (METAB) with major cell lines resources such as Cancer Cell Line Encyclopedia (CCLE), National Cancer Institute 60 panel (NCI60), and the Catalogue Of Somatic Mutations In Cancer panel (COSMIC).

To exclude that the clustering was biased by noncellular factors, we tested whether any association existed between the tree structure and experimental variables. We found the right cluster, named type 1, was associated with batch no. 6 (Fig 3). It must be noted that batch no. 6 included all cell lines that were grown in suspension. All other batches dealt with adherent cells. Rather than pointing to batch effects, we believe that this association highlights fundamental differences between growth conditions. No further association was found. This is a positive outcome as it indicates the clustering was not biased by experimental parameters such as growth rate. The association analysis was extended to metadata available for many cell lines in CCLE (Ghandi *et al*, 2019) on cancer type, histology, histology subtype, pathology, or ethnicity of the cancer donor. In line with Li *et al* (2019a) and the aforementioned clustering of suspended cells, hematopoietic cells were found to be associated with type 1.

We moved on to evaluate whether the observed metabolic types were associated with molecular traits at genomic, transcriptomic, or proteomic level. Our goal was to characterize the differences between metabolic types and to seek for potential upstream regulators that drive the division into robust types. Using mutation data and focusing mainly on cancer genes (Li *et al*, 2019a), we found only one association between a subcluster of type 2, the left cluster, and mutations in Axin 1 (AXIN1). AXIN1 is a component of the beta-catenin destruction complex and thus its mutation can promote the accumulation of this cell-to-cell adhesion molecule (Jeong *et al*, 2018). In copy number data (Li *et al*, 2019a), we found two associations with minor subclusters. For instance, a sub-cluster of type 1 was associated with loss of Cyclin-dependent kinase inhibitor 2B (CDKN2b), which acts as cell growth regulator (Adamovic *et al*, 2008). We next considered the methylation status of cancer genes and found 252 significant associations, thereby highlighting a strong link between epigenetic regulation and metabolic phenotypes. Notable associations are highlighted, like the two negative ones between type 1 and Thrombospondin 1 (THBS1), and CXADR-like membrane protein (CLMP), both linked with cell-to-cell adhesion and interaction. THBS1, an activator of transforming growth factor-beta (TGFβ), has been shown to promote an aggressive phenotype through EMT (Jayachandran *et al*, 2014). In support of this claim, we also found a negative association between type 1 and Eukaryotic translation initiation factor 5A-2 (EIF5A2), known to also induce EMT (Zhu *et al*, 2012; Khosravi *et al*, 2014),

© 2022 The Authors

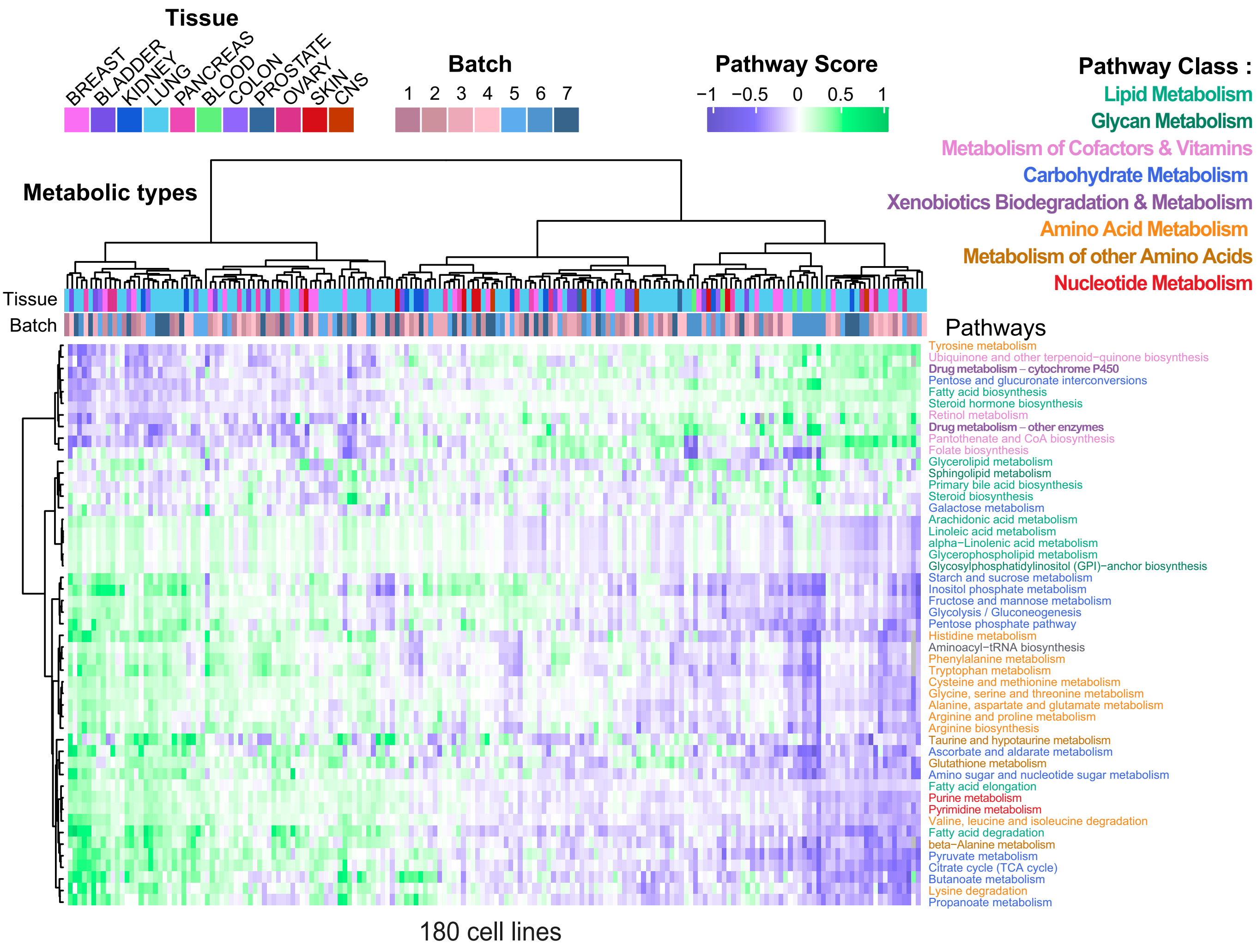

Figure 2. The metabolic activity landscape of cancer cell lines.

Inference of pathway score, a proxy for pathway activity, for 49 KEGG metabolic pathways. Hierarchical clustering of: (above) cell lines, colored by their tissue of origin and the batch they were grown in, (side) metabolic pathways, colored by their pathway class (cell lines $n = 180$, three biological replicates and two technical per cell line averaged).

In the expression data provided by the CCLE, we identified 453 associations. Because of this large number, we focused on genes that could be related to each other using the String database (Szklarczyk *et al*, 2017), to find known interactions or shared biological processes among protein coding genes. Two negative associations were identified between type 1 and CDP-diacylglycerol synthase 1 (CDS1) and lysophosphatidic acid phosphatase type 6 (ACP6). Since both target genes which encode for enzymes involved in the biosynthesis of glycerophospholipids, it may suggest a reduction in lipid synthesis in type 1. We identified positive associations between type 1 and THBS1 expression. As previously observed, THBS1 was less methylated in type 1 and is more expressed in type 1, hence consolidating its association to type 1. Finally, we found 109 proteins significantly associated to some branches, obtained from antibody and mass spectrometry-based (Nusinow *et al*, 2020) approaches (Appendix Fig S1). Of note, p53 levels were lower in type 1 compared to the other types. P and E-cadherin, classical markers of epithelial cells (Ribeiro & Paredes, 2015) had significantly lower levels in type 1.

We hypothesized that the observed, robust metabolic types might be driven by common regulatory mechanisms. Therefore, we tested whether transcription factor activity and signaling pathways are associated with pathway score clusters. For 743 transcription factors (TFs), we assessed whether differential genes were overrepresented in known TF-targets (Ortmayr *et al*, 2019). We identified 115 associations between TFs and the clustered metabolic phenotypes. Several interesting hits were linked to the major type 1 and 1B, middle cluster which bears many similarities to type 1: hypoxia-inducible factor 1-alpha (HIF1A), previously associated with aggressive tumor phenotypes, treatment resistance, and poor clinical prognosis (Wigerup *et al*, 2016); TP63, known to regulate migration, invasion, and *in vivo* pancreatic tumor growth (Somerville *et al*, 2018); and snail family transcriptional repressor 1 (SNAI1), involved in EMT induction. We further tested the activity of 14

 © 2022 The Authors

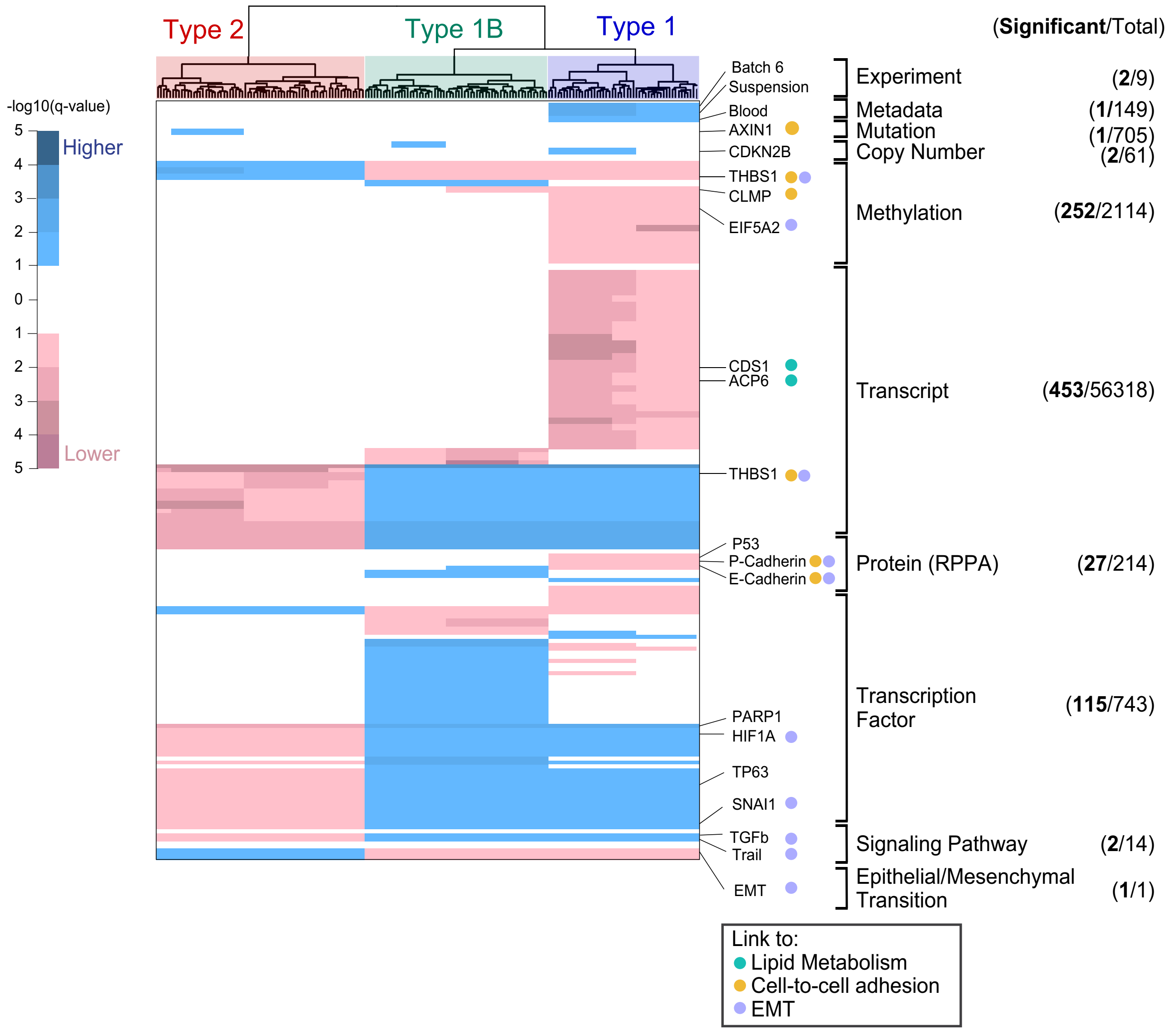

Figure 3. The drivers of metabolic activity phenotypes.

Summary of significant associations between metabolic types, displayed above as hierarchical cluster taken from Fig 2, and multiomics. The list and number of traits integrated to the metabolic phenotype are listed on the right panel, where traits were considered significant at 10% FDR (cell lines $n = 180$). For visualization, q-values were extended with a sign to indicate whether the trait is significantly higher or lower in comparison to the rest of the tree. Only traits significantly associated to the three main types (1, 2, and 1B) are shown for methylation, transcript, protein and transcription. Full results are reported in data availability.

signaling pathways (Schubert *et al*, 2018) and identified a positive association between TGFβ and tumor necrosis factor TNF-related apoptosis-inducing ligand (TRAIL) signaling with type 1 and 1B. Recent findings highlighted the potentially aberrant consequence of TGFβ and TRAIL activation in promoting cell motility and metastasis (Fulda, 2013; Hao *et al*, 2019; Yeh *et al*, 2019).

Association between EMT and metabolic types

Several of the significant associations pointed to an increase of metastasis-related processes, that is, EMT. To directly test this hypothesis, we used the EMT score proposed by Rajapakse *et al* (2018). The score is based on gene expression of known EMT markers to quantify the potential of invasiveness and metastasis formation of cancer. A high EMT score is associated with epithelial state and a low EMT score to mesenchymal state. In our dataset, we could confirm that type 1 and 1B were linked with the mesenchymal state, and type 2 with the epithelial state (last line, Fig 3). We validated the putative EMT association experimentally. We selected representative cell lines of the two main metabolic types 1 and 2, and stained the canonical EMT markers vimentin and E-cadherin using immunofluorescence (Fig EV2). In line with the expectations, the mesenchymal marker vimentin was higher in type 1 (P-value $< 1 \times 10^{-3}$, Student t-test), and the epithelial marker E-cadherin was higher in type 2 (P-value $< 1 \times 10^{-3}$, Student t-test). Microscopy analysis also highlighted the expected morphology

differences. Type 1 cells featured spindle-like shapes resembling fibroblasts, whereas type 2 portraited rounded regular shapes, consistent with EMT progression.

To further substantiate the association to EMT, we have compared the expression of EMT genes across all cell lines (Appendix Fig S2). We found that a high number of EMT genes (57 out of 197) are significantly changing between the types, with notable example of vimentin (VIM) more expressed in type 1 and cadherin (CDH1 and CDH3) more expressed in type 2 (Appendix Fig S2C). A similar trend was found on the protein level for cadherin (Appendix Fig S2D). Altogether, gene expression data, immunostaining and morphology substantiate the association between the main observed metabolic types and EMT.

Differences in metabolic pathway activity unraveled by ^{13}C tracing

The association analysis provided novel leads on the regulatory differences that characterize the main metabolic types, but does not draw robust hypotheses on nutrient utilization or nutrient fluxes. To directly assess differences in pathway usage between the major metabolic types, we used an untargeted ^{13}C-labeling analysis approach. The goal was to confirm whether conserved differences in fluxes could be identified between type 1 and 2. Given the generic preference of cancer cell lines for glucose and glutamine, we grew nine representative but diverse cell lines of the two types in media enriched with either [U-^{13}C]glucose or [U-^{13}C]glutamine for 48 h. Upon metabolite extraction from cells, we used mass spectrometry to measure ^{13}C-enrichment in metabolites and, in turn, to quantify their fractional contribution (FC). Given the experimental design in which a single substrate is labeled, the FC of each detectable metabolite informs on the fraction of carbon that originated from either glucose or glutamine. To highlight differences in carbon fluxes between type 1 and type 2, we computed the difference in FC between the averages of the two types (Fig 4A). This allowed for a ranking of all measured metabolites according to FC differences, with exemplary metabolites with divergent FC displayed in Appendix Fig S3. To consolidate the results at the pathway level, we sought for pathway enrichment in both tails of the ranked metabolite list. For the example of TCA cycle metabolites, the 11 detected metabolites were mostly ranked toward type 2, resulting in a significant enrichment of the pathway with type 2 (q-value < 0.01, Hypergeometric test).

On [U-^{13}C]glucose, the vast majority of pathways of primary metabolism exhibited higher ^{13}C-labeling in type 2 cells (Fig 4B). This indicates that more glucose is used to replenish central carbon metabolism, amino acids, nucleotides, and fatty acids. In contrast, type 1 cells showed a slight enrichment in glucose-derived ^{13}C in the pathways related to carbohydrate metabolism and storage, which are often confused because of the numerous isomers that cannot be resolved analytically. The [U-^{13}C]glutamine revealed less differences between the two types, mostly because the measured FC were generally lower (right panel Fig 4B). This indicates that glutamine-derived carbon is only slightly differentially assimilated between the types.

Alterations in lipid metabolism between metabolic types

Multiple evidence suggested that the main metabolic types might differ in lipid metabolism. To validate this finding, we analyzed the lipidome of seven representative cell lines by LC–MS/MS. We could detect and quantify 305 lipid species (Fig 5) and found that most lipid classes were slightly but reproducibly more abundant in type 1 cell lines (Fig 5A and B, Appendix Fig S4). Despite their minor contribution to total lipids, we stress that total cardiolipins (P-value < 0.01) and phosphatidylglycerols (P-value < 0.05) contents were higher in type 2 (Fig 5C). These lipids constitute the membrane of mitochondria and, therefore, suggest an increase of mitochondrial mass in type 2. Conversely, lipids associated with the extracellular membrane such as phosphatidylserines (P-value < 1×10^{-3}) and sphingomyelins (P-value < 0.01) were higher in type 1, consistent with the spindle-like morphology that requires increased membrane surface. Ether phosphatidylcholines and triacylglycerols were characterized by remarkable within-type shifts (Appendix Fig S4). We thus compared the species by double bond and acyl chain length and found that triacylglycerols had shorter chain length in type 1 cell lines (Appendix Fig S5A) and longer for ether phosphatidylcholines (Appendix Fig S5B). This could point at differences in synthesis compared to uptake in these two lipid classes. Type 2 cell lines had generally higher levels of lipid unsaturation (Fig EV3), including the main constituents of the cell membrane phosphatidylcholines (P-value < 1×10^{-4}) and ether phosphatidylcholines (P-value < 0.01). Lower saturation decreases tight packing of acyl chains and, in turn, increases membrane fluidity (Jain *et al*, 2020).

Given the differences observed in lipid content between the two main metabolic types, we measured differences in lipid biosynthesis rates. To assess how much of the lipids are made *de novo*, we fed cells for 48 h with either [U-^{13}C]glucose or [U-^{13}C]glutamine medium. Lipid extracts were analyzed by LC–MS. Analysis of ^{13}C-labeling in lipids is more challenging than for polar metabolites because of their larger number of carbon atoms which causes a redistribution of the signal detected in an unlabeled sample to dozens of isotopic peaks in a labeling experiment. To maximize the quality of the data, we selected the most abundant representatives for each lipid class and performed targeted data extraction to determine full mass distribution vector.

The resulting data revealed striking differences between the two metabolic types. This is shown exemplarily for TAG 50:1 grown on [U-^{13}C]glucose, an abundant member of triacylglycerols (Fig 5D). In the type 1 representative cell line HS578T, 24% of carbon atoms were labeled and the largest isotopologue was M + 3, which results from the fusion of a $^{13}C_3$-glycerol backbone and unlabeled acyl chains. In contrast, the type 2 representative cell line T47D featured a much higher ^{13}C-enrichment, 50%, with evident incorporation of ^{13}C in the acyl chains. Similar trends were observed for the other representatives of the two types (Fig EV4), with the exception of OVCAR5 that showed a higher enrichment similar to type 2 cell lines. Increased ^{13}C-enrichment indicates potential higher *de novo* fatty acid biosynthesis. If lipogenesis is affected, similar trends should be observable across lipid classes. Indeed, higher ^{13}C-content was observed in 15 abundant lipids from all lipid classes (Fig 5E), and the difference was significant for 11 out of 15 (P-value < 0.05, Wilcoxon–Mann–Whitney test). In the data related to the second tracer [U-^{13}C]glutamine, the labeling enrichment was lower, in the range of 10–20% (Appendix Fig S6). We observed a small but opposite trend with increased ^{13}C in type 1 for 5 of the 15 tested lipids which could reflect a marginal difference in the

 © 2022 The Authors

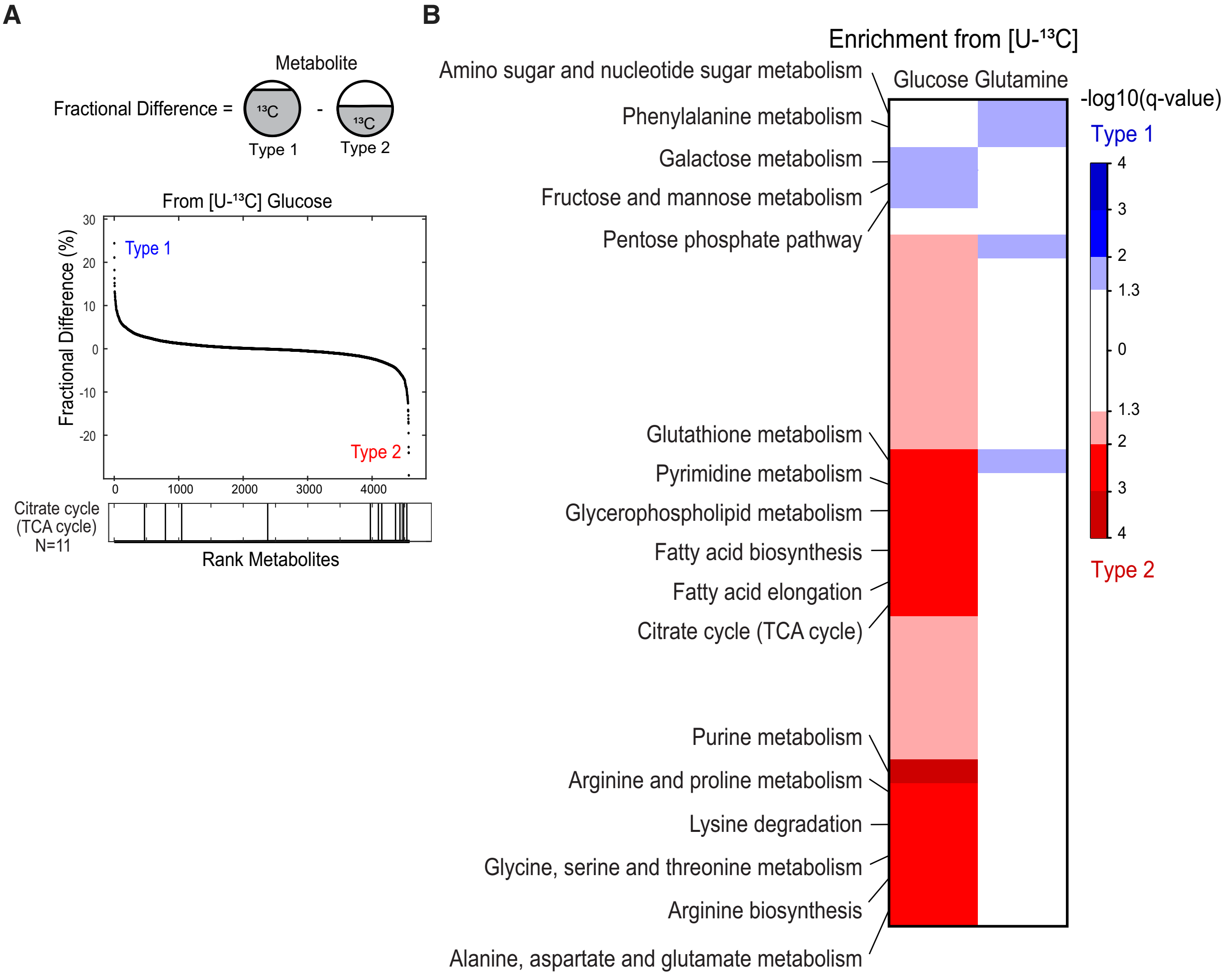

Figure 4. Differential pathway utilization inferred by ^{13}C labeling analysis.

A Schematic representation of fractional difference, difference between each metabolite fractional contribution (percentage of ^{13}C labeled) subtracted between type 1 and type 2. The metabolites are ranked by their fractional difference, where positive values are associated with higher labeling in type 1 and negative values with higher labeling in type 2. The bar plot bellow indicates the position of metabolites of TCA cycle, where seven metabolites out of 11 were more labeled in type 2.

B Metabolic pathways enrichment computed using metabolites fractional differences. Pathways associated to higher labeling in type 1 are displayed in blue and in type 2 in red (cell lines $n = 9$ with three biological replicates, hypergeometric test corrected for FDR). Results of labeling experiment using [U-^{13}C]glucose or [U-^{13}C]glutamine denoted on top of heatmap.

fraction of citrate that originates from glutamine and provides acetyl-CoA monomers to lipogenesis. In conclusion, we observed higher activity in *de novo* lipid synthesis (from the main carbon source glucose) in type 2 cells, which was not coupled to higher lipid content. The remaining fraction of nonlabeled lipids resulted from other carbon sources, which could be from direct lipid uptake.

Main metabolic types have distinct genes and drug sensitivity

To functionally validate the pathway scores, we evaluated if the inferred metabolic types were associated to differences in sensitivity to genetic or pharmacological inhibition. We used the dependency data from a CRISPR knockout screen of 18,333 genes (Tsherniak *et al*, 2017), for which 63 cell lines overlap with the two types. We found that active pathways were more sensitive in one type versus the other. For example, in upper glycolysis we found that phosphoglucomutase 3 (PGM3) and phosphoglycerate mutase 1 (PGAM1) deletion had stronger effect in type 1 (Student *t*-test P-value < 0.05 and P-value < 0.01, respectively; Fig 6A and B). Indeed, PGAM1 knockout has a deleterious effect in both types. However, we observed that the effect was significantly more pronounced for type 1 (−1 vs −0.8 in type 2, Student *t*-test P-value < 0.01) and close to the median value of essential genes (Tsherniak *et al*, 2017). These two results corroborated from a functional standpoint the association of type 1 to higher sugar metabolism activity. Inversely, the sensitivity to gene knockout shifted between types in the TCA cycle. For example, knockout of isocitrate dehydrogenase 2 mitochondrial (IDH2) or succinate dehydrogenase assembly factor 4 mitochondrial (SDHAF4) affected the growth of type 2 cells (P-value < 0.01) more than type 1.

© 2022 The Authors

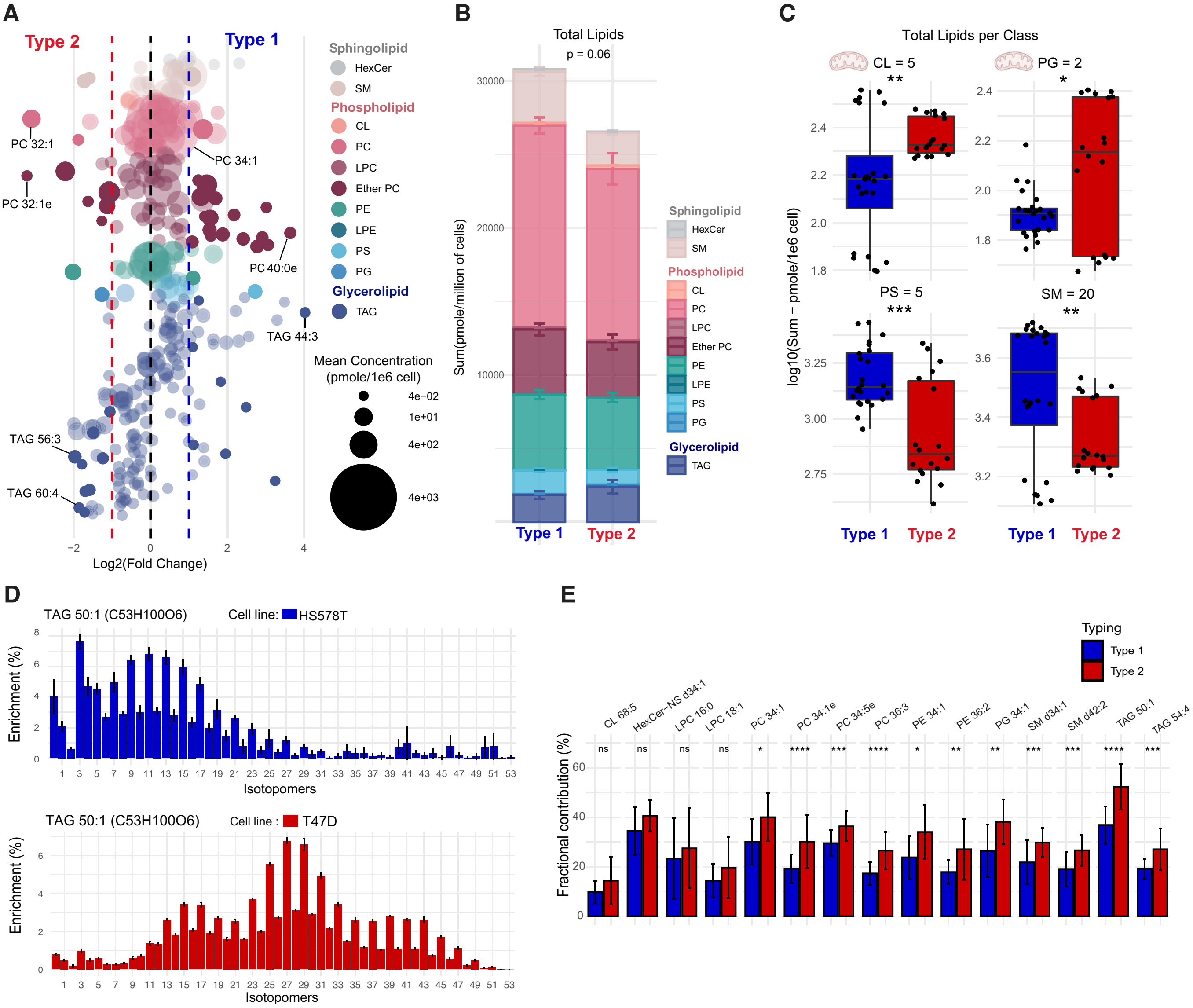

Figure 5. Differences in lipid content and *de novo* biosynthesis between metabolic types.

A Comparison of lipid species' relative abundance between type 1 and 2, sorted by lipid class. Statistically significant lipids are identified by solid colors (adjusted $P < 0.05$, cell lines $n = 7$ with three biological replicates per cell line and two technical replicates, two-sided unpaired student t-test, corrected for FDR).

B Comparison of total lipid content, with mean sum of lipid class ± standard error (cell lines $n = 7$ with three biological replicates per cell line and two technical replicates, two-sided unpaired student t-test).

C Comparison of total content in individual lipid class, with number of lipids measured by class in title. Lipids class linked to mitochondria identified with the schema of the organelle (cell lines $n = 7$ with three biological replicates per cell line and two technical replicates, boxplot depicts first quartile, median, and third quartile, two-sided unpaired student t-test). ns: $P > 0.05$, $^*P < 0.05$, $^{**}P < 0.01$, $^{***}P < 0.001$, $^{****}P < 0.0001$.

D Mass distribution vector of TAG 50:1 labeled from [U-^{13}C]glucose of two breast cancer cell line, HS578T, type 1, and T47D, type 2 (three biological replicates per cell line and two technical replicates, mean ± standard deviation).

E Bar plot (mean ± standard deviation) of fractional contribution from [U-^{13}C]glucose of most abundant lipids per lipid class (cell lines $n = 9$ with three biological replicates per cell line and two technical replicates, Wilcoxon–Mann–Whitney test). ns: $P > 0.05$, $^*P < 0.05$, $^{**}P < 0.01$, $^{***}P < 0.001$, $^{****}P < 0.0001$. Abbreviation: hexosylceramide (HexCer), sphingomyelin (SM), cardiolipins (CL), phosphatidylcholines (PC), lysophosphatidylcholines (LPC), ether phosphatidylcholines (ether PC), phosphatidylethanolamine (PE), lysophosphatidylethanolamine (LPE), phosphatidylserine (PS), phosphatidylglycerols (PG), and triacylglycerols (TAG).

We extended the analysis from single reactions to whole pathways. We ranked all 18,333 genes on their correlation with the two types and performed gene set enrichment analysis to identify pathways that include genes whose knockout lead to differential effects. The top pathways associated with type 1 were linked to sugar metabolism (Fig 6C). More strikingly, the pathways whose knockout caused frequently a growth defect in type 2 were oxidative phosphorylation (q-value = 0, Appendix Fig S7A) and biosynthesis of unsaturated fatty acid (q-value < 0.05, Appendix Fig S7B). These are in line with the higher activity predicted by pathway score and

 © 2022 The Authors

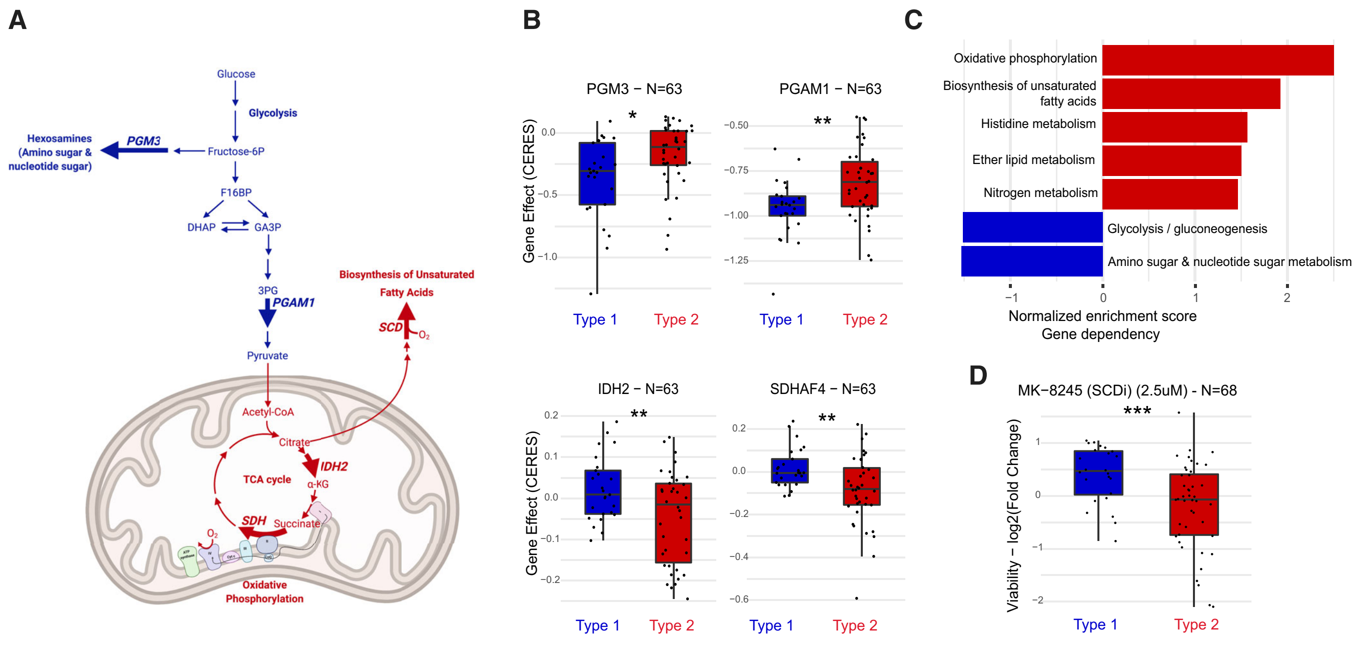

Figure 6. Sensitivity to genetic and pharmacological inhibition between metabolic types.

A Schematic representation of pathways depicting analyzed reactions. Gene effect (CERES score (Meyers *et al*, 2017)) estimate gene-dependency levels from CRISPR-Cas9 knock-out screens on cell line's growth and is scale so that the median score of common essentials genes is −1.

B Gene effect of PGM3, *phosphoglucomutase 3*, from the amino sugar pathway PGAM1, *phosphoglycerate mutase 1*, from glycolysis SDHAF4, *succinate dehydrogenase assembly factor 4 mitochondrial*, and IDH2, *isocitrate dehydrogenase 2 mitochondrial*, both from the TCA cycle (cell lines $n = 63$, two-sided unpaired student t-test).

C Top dependent metabolic pathways in type 1 (blue) or type 2 (red), where normalized enrichment scores are computed using GSEA.

D Growth response to drug MK-8245, which targets SCD, *stearoyl-CoA desaturase*, from the unsaturated fatty acids biosynthesis pathway.

Data information: Boxplot depicts first quartile, median and third quartile, two-sided unpaired student t-test. ns: $P > 0.05$, *$P < 0.05$, **$P < 0.01$, ***$P < 0.001$, ****$P < 0.0001$.

verified by ^{13}C-experiments in the TCA cycle and *de novo* lipogenesis (e.g., PC 34:5e in Fig 5E), respectively. The differentiating relevance of unsaturated fatty acids was confirmed by drug sensitivity data (Corsello *et al*, 2020). Type 2 cells were more susceptible to inhibition of stearoyl-CoA desaturase (SCD), a major contributor for biosynthesis of unsaturated fatty acids (Fig 6D). In summary, we demonstrated that pathway scores and clustering derived from metabolomics data can translate into dependency for cancer cell lines and sensitivity to genetic or pharmacological inhibition. In type 2, the importance of mitochondrial pathways was highlighted by oxidative phosphorylation dependency and the sensitivity to unsaturated fatty acid biosynthesis was confirmed as a therapeutic liability for these cancers.

Discussion

We used a systematic approach to investigate the metabolic reprogramming in 180 cancer cell lines grown *in vitro* in comparable and controlled conditions. Starting from semiquantitative data for 1,809 putative deprotonated metabolites profiled by untargeted metabolomics, we estimated activity scores for 49 pathways by principal component analysis. Unsupervised clustering of cell lines based on pathway scores revealed two main groups, pointing to convergence of metabolic phenotypes. The emergence of only a few overarching groups was unexpected and, therefore, we further characterized the two major cell line metabolic types by computational and experimental means. The two metabolic types differ in pathway usage. Type 1 has enhanced carbohydrate metabolism, and type 2 relies on mitochondrial pathways, amino acid metabolism, and lipogenesis. The activity of these pathways was confirmed by isotopic labeling, and their central role was confirmed by a knockout fitness screen. Our results are coherent with the subtypes identified by Daemen *et al* (2015) in pancreatic ductal adenocarcinomas but generalizable to all cancer lineages and a multitude of pathways in primary and lipid metabolism.

Building on the insights from the first large-scale intracellular metabolomics study on cancer cell lines (Li *et al*, 2019a), we expanded their work by taking a completely different approach to data analysis. Rather than focusing on how single genetic perturbations affect single metabolites, which is covered by Shorthouse *et al* (2022), we have inferred pathway activity for each cell line to unravel metabolic changes at the level of the whole metabolic network. This novel computational approach, which has also been proposed for the analysis of transcriptomics (Segura-Lepe *et al*, 2019), combined with our wider metabolite coverage has enabled the identification of global cancer metabolic phenotypes, for which we have confirmed their functional relevance by orthogonal methods.

The integration of global metabolic phenotypes to all available traits allows for the identification of possible metabolic drivers of

© 2022 The Authors

the phenotype. Our analysis revealed many putative drivers of the major metabolic types, that is HIF1A, TGFβ, and the EMT status. Their role in regulating part of metabolism is well-established (Semenza, 2012; Hua *et al*, 2019; Georgakopoulos-Soares *et al*, 2020), but our work highlights how they dominate over other regulatory axes. Moreover, the association between pathway activity and regulators might also reflect reversed causality, which could point to metabolites modulating a regulator (Jia *et al*, 2021). This could be the case, for the amino sugar pathway that was found to be more active in type 1. Its main product, UDP-acetylglucosamine, is known to affect glycosylation and, in turn, EMT (Akella *et al*, 2019). Therefore, the metabolite change might act upstream of EMT, and not vice versa. Further analyses would be required to verify causality of these relations as the cause-and-effect relationship between EMT and metabolic reprogramming remains elusive (see review by Jia *et al* (2021)). Interestingly, the association of the types with EMT shows a much greater interaction and interconnectedness between malignant factors and global metabolic reprogramming (Georgakopoulos-Soares *et al*, 2020), identifying relation outside of previously well-studied pathways. This greater EMT-metabolism relation was not unexpected as changing motility phenotypes would necessitate altered bioenergetics and, thereby, extensive alteration in metabolism (Jia *et al*, 2021). Interestingly, a study of 949 cancer cell lines has also reported the dominant role of EMT in explaining proteome variability (Gonçalves *et al*, 2022).

What has yet to be explored are the principles that drive these cancer cells to adopt different metabolic program. These could be associated to specific limitations that cancer cells have to bypass to support their development and transformation, such as adaptation to hypoxia, or to solve whole-cell challenges of efficient energy production or proteome allocation (Basan *et al*, 2015). Because of the overwhelming result linking type 2 to aerobic pathways (TCA cycle, oxidative phosphorylation, unsaturated fatty acids, etc.), we hypothesize that oxygen play a major role in shaping the metabolic phenotype. Even though cells were grown under the same normoxic condition, hysteresis due to past oxygen availability could explain these phenotypes. In fact, type 1 cells are characterized by ubiquitous changes that characteristic of hypoxia: activation of HIF1 targets, inhibition of mitochondrial pathways, and increase in lipid uptake, which has been shown to be beneficial against hypoxic stress (Kamphorst *et al*, 2013). Type 2 cells, in contrast, maintain membrane fluidity by producing *de novo* unsaturated fatty acids fueled by the TCA cycle. Moreover, the differences in these aerobic pathway usages could be explained by impaired mitochondria. In fact, type 1 cells had less mitochondrial lipids, less activity in mitochondrial pathways, and were less dependent on respiration. Future work should verify causality, that is, whether mitochondrial dysfunctions, the loss of mitochondrial mass, or stabilization of HIF1 are sufficient to drive shift type 2 cells to type 1.

Materials and Methods

Cell Culture

A total of 182 cell lines were obtained and grown in seven distinct batches over the period of several months. To minimize the effect of the environment, all cell lines were adapted to growth in the same media by maintaining them in culture for at least 2 weeks. Cells were cultured in RPMI 1640 Phenol Red Free (Sigma #R7059) with 2 mM L-glutamine (Sigma #G7513, Lot #RNBD0904) freshly added. The medium was supplemented with 10% fetal calf serum (PAA Laboratories, Linz, Austria, #A15-701, Lot #A30111-3524) and is referred hereafter as RPMI, 10% FCS, L-glutamine. Cell lines were grown at 37°C with 5% CO_2. Each cell line was seeded into 3 wells of 6-well plates and grown for 48 h. Cell lines were maintained according to standard protocols, their identity verified via STR sequencing and tested for mycoplasma infections. All adherent cells were grown to reach similar confluency. The cut-off values for adherent cell lines are a minimum of 50%, and a maximum of 80% confluency. For mixed suspension/adherent lines, they were judged on a case-by-case basis, and the minimum confluency as it does not reflect direct cell density set at 35%. For suspension cells, after 48 h, cell culture counts were accessed in the count plate on an automated cell counter (Cedex, by Roche, Basel, Switzerland). As there was little or no growth lag after splitting the culture, we calculated growth rates from recent counts and seeded accordingly. The final density was at the upper end of the exponential phase of growth.

Metabolite extraction

At 48 h, the medium was removed via aspiration, and cells were washed twice with a wash solution (75 mM Ammonium Carbonate, adjusted to pH 7.4 with Acetic Acid). Metabolites were quenched by dipping the bottom of the plate in liquid nitrogen for 1 min. Metabolites were extracted using a 40% methanol, 40% acetonitrile, and 20% water solvent. The plate was sealed and incubated at −20°C for 10 min. Extracted cells were scraped off the bottom of each well using a pipet with wide-bore tips. Next, the cell extracts were transferred to 96-well plates with conical bottom and centrifuged at 4°C, 2,800 rpm for 30 min to separate cell debris. The cleared supernatants were injected for mass spectrometric analysis.

Untargeted metabolomics

Untargeted metabolite profiling was performed using flow injection analysis on an Agilent 6550 QTOF instrument (Agilent, Santa Clara, CA) using negative ionization, 4 GHz high resolution acquisition, and scanning in MS1 mode between m/z 50 and 1,000 at 1.4 Hz (Fuhrer *et al*, 2011). The solvent was 60:40 isopropanol:water supplemented with 1 mM NH_4F at pH 9.0, as well as 10 nM hexakis (1H, 1H, 3H-tetrafluoropropoxy)phosphazine and 80 nM taurochloric acid for online mass calibration. The seven batches were analyzed sequentially. Within each batch, the injection sequence was randomized. Data were acquired in profile mode, centroided and analyzed with Matlab (The Mathworks, Natick). Missing values were filled by recursion in the raw data. Upon identification of consensus centroids across all samples, ions were putatively annotated by accurate mass and isotopic patterns. Starting from the HMDB v3.0 database (Wishart *et al*, 2013), we generated a list of expected ions including deprotonated, fluorinated, and all major adducts found under these conditions. All formulas matching the measured mass within a mass tolerance of 0.001 Da were enumerated. As this method does not employ chromatographic separation or in-depth MS2 characterization, it is not possible to distinguish between

 © 2022 The Authors

compounds with identical molecular formula. The confidence of annotation reflects Level 4 but—in practice—in the case of intermediates of primary metabolism, it is higher because they are the most abundant metabolites in cells. The resulting data matrix included 1,809 ions that could be matched to deprotonated metabolites listed in HMDB v3.0. All *m/z* peaks that remained unmatched or were associated to adducts or heavy isotopomers were discarded.

Data normalization

As first-line quality control for each measurement, we computed the sum of all intensities (TIC) of each injection. If extremely low or high, the TIC is a sign of problems in the sample preparation, injections, or measurement. Out of the 1,387 injections, 68 exhibited abnormal TIC and were removed. These included all six replicates of PC14PE6 and NCIH446. Out of the initial 182 cell lines included in the screen, we were left with data for 180. Large-scale untargeted metabolomics experiments are subject to various factors that introduce unwanted effects on data: batch effects of sampling and analysis, drifts in measurements, accumulation of dirt, drops in instrument sensitivity, etc. To address these potential issues, we tested numerous normalization methods. To assess the efficacy of each method, we adopted quantitative measures of reproducibility.

The fundament of the reproducibility analysis was the inclusion of the two cell lines as quality controls (QC): MCF7 and MDAMB321 in each of the seven growth batches. Because of sequential analysis of batches, representatives of both cell lines were distributed over the entire injection sequence. The expectation was that all repeated measurements of either MCF7 or MDAMB321 had to be similar, and differences between MCF7 and MDAMB321 had to be preserved across the seven batches. The reproducibility metrics included the following criteria:

- Batch Scoring Fold Change (FC): calculated as the mean fold-change between random subsets of MCF7 cell line measurements. Out of the 66 replicate measurements for this cell lines across all batches, we randomly sampled two sets of 6 samples, calculated the mean intensities for each set and, in turn, the fold-change for each of the 1,809 metabolites ions. Sampling was repeated 1,000 times. From the distribution of all fold-changes, we calculated the Batch Scoring FC as the threshold that corresponds to the 5% false discovery rate. Ideally, Batch Scoring FC between replicates of the same cell line should be zero. Normalization improves reproducibility if the threshold value decreases.
- FC Reproducibility: for each batch, we calculated the fold-change for all 1,809 metabolite ions between the MCF7 and MDAMB321 replicates. We calculated the mean Euclidean distance between the fold-changes of all seven batches. Normalization improves reproducibility if the distance between fold-changes across batches decreases.
- FC Reproducibility amino acids (AA): for each batch, we calculated the fold-change between the MCF7 and MDAMB321 replicates for ions feature matching to AA. We calculated the mean Euclidean distance between the fold-changes of all seven batches. Normalization improves reproducibility if the distance between fold-changes decreases.
- Interbatch Distance: we adopted the metric defined by Wehrens *et al* (2016). It applies principal component analysis and computes the how overlapping are the batches the Bhattacharyya distance. Normalization improves reproducibility if the distance decreases.
- Kolmogorov–Smirnov: we tested for all ions whether MCF7 samples from different batches were on the same distribution. A two-sample Kolmogorov–Smirnov was used to test for this batch effect. We counted the frequency of tests that revealed batch effects, by counting the number of tests significant (P-value < 0.05) on the total number of tests done.

The five criteria were scaled to the not normalized data (value of 1), where 1 denotes no amelioration of reproductivity and values bellow 1 improved the reproducibility.

We tested 15 state-of-the-art normalization methods. Conceptually, these methods can be divided into three different classes:

1. Batch Effect: Methods that correct for user-defined batches. In our case, we used the seven experimental batches. We tested two implementations of Remove Unwanted Variation (RUV) method (Risso *et al*, 2014) and the empirical Bayes method ComBat (Johnson *et al*, 2007). In some cases, MDAMB321 samples were selected as QC samples used for correction.
2. Signal Drift: Methods that correct for signal drifts that occur chronologically during the injection sequence. The drifts might be caused by smooth changes in solvents, the ionization source, ion optics, or the detection process. We used three methods that consider the injection sequence to detect temporal drifts and correct after smooth interpolation. First, we implemented a method that applies a moving median (window 120 min) to all measured samples to estimate a robust trendline. Second, we used a locally weighted regression (LOESS; Cleveland & Devlin, 1988) and its derivate for temporal trends (Robust LOESS; Cleveland, 1979), and third, we used the QC-based support vector regression method (QC-SVR; Kuligowski *et al*, 2015). In the latter case, the MDAMB321 samples and the plate order were selected as QC samples to be used for correction.
3. Sample Variance: Due to differences in cell amount, sampling, pipetting errors, injection errors, etc. variations can occur in single samples. To correct for such issues, we tested normalization using quantiles (Quantile), log10-mean (Mean), median (Median), standard deviation (Std), median absolute deviation (Mad), the sum of all ions (TIC), probabilistic quotient (PQN), and scaling with cellular confluency at the time of sampling (Confluency).

These methods were tested singularly and in reasonable combinations (Combo). Given that the three classes tackle different types of problems, we have combined different methods from the different classes. The choice of methods and the order of combinations was based on their improvement of quality metric scores. Results and quality score are reported in Table EV1.

Metabolic pathway definition

A common issue using pathways is that their definition can be arbitrary, that is the start and end of a pathway is dependent on the database. Kyoto Encyclopedia Genes and Genomes (KEGG; Kanehisa & Goto, 2000), the chosen database because of its high curation, has the disadvantage of defining substantially

© 2022 The Authors

overlapping pathways. We circumvented this limitation by removing reactions, and their corresponding substrate or product, which were present in multiple metabolic pathways. This curation resulted in a smaller pathway definition with less overlapping reactions, and thus resulting in metabolites more specific to a pathway. Out of the 1,809 putatively annotated ions, 367 could be linked to KEGG pathways. As in many cases, the measurement does not allow to distinguish between structural isomers, ion could match to one or multiple metabolites with the same formula. In total, the 367 deprotonated ions matched to 530 metabolites which are part of KEGG metabolic pathways.

Pathway activity scoring

Our goal is to infer flux changes from relative metabolite abundances, one pathway at the time. At the biochemical level, the relation between metabolites and fluxes is governed by enzyme kinetics and capacity. If pathway flux change, we expect all intermediate concentrations to shift in a coherent direction. In some cases, metabolite changes may only be subtle, depending on enzyme saturation and the additional regulatory mechanisms. In general, coherent and distributed changes of pathway metabolites indicate an underlying flux change. In contrast, strong but local metabolite changes reflect pathway interruption (Fuhrer *et al*, 2017) or changes in enzyme kinetics or abundance that are compensated by a modulation of the reactants but do not influence pathway flux (Fendt *et al*, 2010). Hence, we sought to quantify ubiquitous and coordinated changes in multiple metabolites within each pathway. This was done by principal component analysis (PCA). PCA identifies components which best capture metabolite variance. As we are interested in the major metabolite effect, we focused on the first principal component (PC1) and used the PC1 scores as proxy of pathway activity. The same principle has been adopted in the past for the analysis of transcriptomics data (Segura-Lepe *et al*, 2019). To verify the validity of the method in the case of metabolomics, we used a published dataset by Hackett *et al* (2016), which offered both metabolomics and measured fluxes for multiple conditions with sufficiently diverse flux distributions (Fig EV1). For representative pathways, we correlated PC1 scores and measured fluxes. Strong positive correlation is observed between the glycolytic and pentose phosphate metabolites summarized in PC1 and the fluxes of these pathways. To be noted, the direction of the pathway activity score can be reverse, for example, in the case of the purine pathway in Fig EV1C, where a high score denotes low flux. Overall, the PC1 scores correlated favorably with fluxes in all cases tested.

Inference of pathway score in cancer cell lines

Metabolomics data were mapped onto curated KEGG pathways, where only pathways with a minimum of four measured metabolic ions with unique m/z were considered for further analyzed. Regardless of the number of detectable metabolites, the relative pathway activity score for each cell line replicate was obtained by PCA. For each cell line, we averaged the 6 independent scores to assess the pathway activity score. Final scores were scaled to [−1...1] for comparison across pathways.

Metabolic typing and omics association analysis

Metabolic types were identified using hierarchical clustering using Ward's method of pathway scores.

The resulting hierarchical clustering tree of the cell lines was used for omics association analysis, where association over the tree were computed by iterating through all branches of the tree with at least 18 cell lines (10% of the total number). For categorical traits (e.g., batch number or genomic data), we used a hypergeometric test to evaluated if a trait was over-represented in a branch. For continuous traits (e.g., gene expression), we used a Student two-tailed *t*-tests to identify if a trait was over or under expressed in a branch. We assembled all (1,025,576) resulting *P*-values and corrected *in toto* for false discovery rate by the Storey and Tibshirani method to produce *q*-values (Storey & Tibshirani, 2003).

Selection of cell lines for follow ups

We chose 9 cells from type 1 and type 2 for further evaluation (Table EV3). These cell lines were selected to span diverse lineages and growth rates. To show differences in metabolism across the same lineage, we selected pairs of ovarian cell lines (OVCAR3 and OVCAR5) and of breast cell lines (T47D and HS578T) belonging to different types. To confirm that the metabolic types are not associated with growth rate, we selected cell lines with doubling time spanning from 17 to 53 h and mixed type. Cell lines were obtained from the National Cancer Institute (NCI, Bethesda, MD, USA). After thawing, the cell lines were expanded in cell culture flasks (Nunc T75, Thermo Scientific) at 37°C and 5% CO_2 in RPMI-1640 (Biological Industries, cat.no. 01-101-1A) supplemented with 5% fetal bovine serum (FBS, Sigma Aldrich, cat.no. F6178), 2 mM of L-glutamine (Gibco, cat.no. 25030024), 2 g/l of D-glucose (Sigma Aldrich, cat.no. G8644), and 100 U/ml of penicillin/streptomycin (P/S, Gibco, cat.no. 15140122).

Cell imaging and image analysis

Cell lines were fixed with 10% formalin (Sigma-Aldrich #F8775) for 10 min at room temperature. After fixation, cells were permeabilized and unspecific antibody binding was blocked by incubating them with a 10 mg/ml of bovine serum albumin (BSA) solution containing 0.01% (v/v) Triton-X100 (Sigma-Aldrich #T8787) and 10 μg/ml of DAPI (BioLegend #422801) for 30 min at room temperature. Cells were then stained in blocking solution (1 mg/ml BSA in PBS) overnight at 4°C with the following antibodies: AlexaFluor® 488 anti-Vimentin (1:1,000, Biolegend, #677809), AlexaFluor® E-cadherin (1:200, clone 36/E-cadherin, BD #560062), and DAPI (10 mg/ml, 1:1,000, Sigma Aldrich). High-content imaging was performed with an Opera Phenix automated spinning-disk confocal microscope at 40× magnification (Perkin Elmer, HH14000000). To measure cell area shape features, single cells were segmented using CellProfiler 2.2.0 (McQuin *et al*, 2018). Nuclei segmentation relied on the DAPI channel. CellProfiler module "DetectPrimaryObject" was used to identify the nuclei and "DetectSecondaryObject" was used to derive the intensity of the marker in the area around the nucleus. For each cell, nine images from nine biological replicates were segmented, which cumulated into 10,273 segmented cells. Statistical significance was assessed by Student's *t*-test.

 © 2022 The Authors

^{13}C labeling experiments

After two passages, FBS in the growth medium was replaced by dialyzed FBS with a reduced content of low molecular weight compounds (dFBS, Sigma Aldrich, cat.no. F0392). Three replicates of each cell line were grown for 48 h in either growth medium with either [U-^{13}C] glucose (Sigma-Aldrich, cat.no. 389374), [U-^{13}C] glutamine (Cambridge Isotope Laboratories, cat.no. CLM-1822-H-PK), or naturally labeled growth medium. At 48 h, we extracted metabolites as described and analyzed on Agilent 6546 Q-TOF instrument (Agilent, Santa Clara, CA). Mass spectra were recorded from 50 to 1,050 *m/z* in 4 GHz HighRes, negative ionization mode. Annotation was done by matching the measured mass of the ions with reference compounds derived from the Human Metabolome Database (HMDB 4.0), taking labeling patterns of potential metabolites into consideration. For each measured metabolite, mass distribution vector and fractional contribution have been computed using formulas described in the following review (Buescher *et al*, 2015).

Lipidomics

Cells were grown in the same three conditions as above: naturally labeled, [U-^{13}C] glucose, and [U-^{13}C] glutamine medium. At 48 h, internal standard (EquiSPLASH, Avanti Polar Lipids, cat.no. 330731) was added to all cell lines to enable the quantification of lipid species. Lipid extraction was performed using 50:50 (v/v) methanol/isopropanol for 1 h at −20°C. Untargeted lipidomics was performed by LC–MS on a Thermo Fisher Q-Exactive HF-X mass spectrometer (Thermo, Massachusetts, United States). For liquid chromatography, we used a 30 mm Waters ACQUITY UPLC BEH C18 column (cat. no. 186002352) and a 7 min gradient from 15% buffer B (90% (v/v) isopropanol, 10% acetonitrile, 10 mM of ammonium acetate) and 85% buffer A (60% acetonitrile, 50% water, 10 mM of ammonium acetate) to 99% Buffer B. Mass spectra were recorded from 150 to 2,000 *m/z* in positive ionization mode, recording MS1 and MS2 (DDA, top 5 ions) spectra. Lipidomics data processing for nonlabeled samples was done using Compound Discoverer 3.1 (Thermo, Massachusetts, United States). Lipids were annotated with MS2 information. Lipids from each class were quantified using class-specific internal standards.

For labeled lipids, we adopted a targeted data extraction. The most abundant representatives for each lipid class were selected in naturally labeled samples. All ^{13}C-isotopomer traces were extracted as ion chromatograms from labeled samples based on accurate mass and retention time. Related mass isotopomers were integrated with identical boundaries and normalized to unity to obtain labeling fractions.

Gene dependency and drug response analysis

Gene dependencies were obtained from Tsherniak *et al* (2017) and the response from Corsello *et al* (2020). Gene set enrichment was performed using GSEA (https://www.gsea-msigdb.org/gsea/index.jsp) leading-edge analysis (Subramanian *et al*, 2005) by correlating gene dependency score to the two types. Gene sets were taken from the curated KEGG pathways described above. We used 1,000 permutations of the gene-level values to calculate normalized enrichment scores and statistical significance. GSEA results display the enrichment score normalized to mean enrichment of random samples of the same size.

Data availability

Raw metabolomics files of the 180 cancer cell lines can be accessed from the Massive database (https://massive.ucsd.edu/ProteoSAFe/dataset.jsp?accession=MSV000087155). Data tables and raw files of follow up experiments are available at https://doi.org/10.3929/ethz-b-000511784. Code is available at https://github.com/zamboni-lab/CCL180-analysis.

Expanded View for this article is available online.

Acknowledgments
We thank Mattia Zampieri for providing the cell lines for the follow-up experiments, the transcription factor dataset, and for his advice. We thank Julio Saez-Rodriguez, Aurélien Dugourd, Enio Gjerga, and Panuwat Trairatphisan for providing the signaling pathway dataset. We thank Jonathan DeGeer for his comments on the manuscript. This project was supported by the Swiss National Science Foundation (Grant #156319) and by a ETH Zurich Research Grant (ETH-39 16-2).

Author contributions
Sarah Cherkaoui: Conceptualization; data curation; formal analysis; validation; investigation; visualization; writing – original draft; writing – review and editing. **Stephan Durot:** Validation. **Jenna Bradley:** Investigation. **Susan Critchlow:** Supervision; writing – original draft. **Sebastien Dubuis:** Formal analysis; investigation. **Mauro Miguel Masiero:** Validation; investigation. **Rebekka Wegmann:** Formal analysis; validation. **Berend Snijder:** Supervision. **Alaa Othman:** Validation; methodology. **Claus Bendtsen:** Resources; supervision; writing – review and editing. **Nicola Zamboni:** Supervision; writing – original draft; project administration; writing – review and editing.

In addition to the CRediT author contributions listed above, the contributions in detail are:
SCh and NZ designed the study. JB and SCr carried out the original cell lines experiments. SeD performed metabolomic measurements. SCh performed the bioinformatic analysis. StD and SCh performed follow-up cell line experiments, labeling, and lipidomic. AO assisted lipidomic measurements and analyzed lipidomic data. NZ supported labeling analysis. RW and SCh performed microscopy measurement and analysis. MMM evaluated drug response. CB and BS supervised parts of the study. NZ supervised the whole study. SCh and NZ wrote the manuscript. All authors reviewed and approved the final manuscript.

Disclosure and competing interests statement
The authors declare that they have no conflict of interest.

References

Adamovic T, Hamta A, Roshani L, Lü X, Röhme D, Helou K, Klinga-Levan K, Levan G (2008) Rearrangement and allelic imbalance on chromosome 5 leads to homozygous deletions in the CDKN2A/2B tumor suppressor gene region in rat endometrial cancer. *Cancer Genet Cytogenet* 184: 9–21

© 2022 The Authors

Akella NM, Ciraku L, Reginato MJ (2019) Fueling the fire: emerging role of the hexosamine biosynthetic pathway in cancer. *BMC Biol* 17: 52

Bar-Even A, Noor E, Savir Y, Liebermeister W, Davidi D, Tawfik DS, Milo R (2011) The moderately efficient enzyme: evolutionary and physicochemical trends shaping enzyme parameters. *Biochemistry* 50: 4402 – 4410

Basan M, Hui S, Okano H, Zhang Z, Shen Y, Williamson JR, Hwa T (2015) Overflow metabolism in *Escherichia coli* results from efficient proteome allocation. *Nature* 528: 99 – 104

Buescher JM, Antoniewicz MR, Boros LG, Burgess SC, Brunengraber H, Clish CB, DeBerardinis RJ, Feron O, Frezza C, Ghesquiere B *et al* (2015) A roadmap for interpreting (13)C metabolite labeling patterns from cells. *Curr Opin Biotechnol* 34: 189 – 201

Chen P-H, Cai L, Huffman K, Yang C, Kim J, Faubert B, Boroughs L, Ko B, Sudderth J, McMillan EA *et al* (2019) Metabolic diversity in human non-small cell lung cancer cells. *Mol Cell* 76: 838 – 851

Cleveland WS (1979) Robust locally weighted regression and smoothing scatterplots. *J Am Stat Assoc* 74: 829 – 836

Cleveland WS, Devlin SJ (1988) Locally weighted regression: an approach to regression analysis by local fitting. *J Am Stat Assoc* 83: 596 – 610

Corsello SM, Nagari RT, Spangler RD, Rossen J, Kocak M, Bryan JG, Humeidi R, Peck D, Wu X, Tang AA *et al* (2020) Discovering the anticancer potential of non-oncology drugs by systematic viability profiling. *Nat Cancer* 1: 235 – 248

Daemen A, Peterson D, Sahu N, McCord R, Du X, Liu B, Kowanetz K, Hong R, Moffat J, Gao M *et al* (2015) Metabolite profiling stratifies pancreatic ductal adenocarcinomas into subtypes with distinct sensitivities to metabolic inhibitors. *Proc Natl Acad Sci U S A* 112: E4410 – E4417

Dang L, White DW, Gross S, Bennett BD, Bittinger MA, Driggers EM, Fantin VR, Jang HG, Jin S, Keenan MC *et al* (2009) Cancer-associated IDH1 mutations produce 2-hydroxyglutarate. *Nature* 462: 739 – 744

Dayton TL, Jacks T, van der Heiden MG (2016) PKM2, cancer metabolism, and the road ahead. *EMBO Rep* 17: 1721 – 1730

DeBerardinis RJ, Mancuso A, Daikhin E, Nissim I, Yudkoff M, Wehrli S, Thompson CB (2007) Beyond aerobic glycolysis: Transformed cells can engage in glutamine metabolism that exceeds the requirement for protein and nucleotide synthesis. *Proc Natl Acad Sci U S A* 104: 19345 – 19350

Elia I, Haigis MC (2021) Metabolites and the tumour microenvironment: from cellular mechanisms to systemic metabolism. *Nat Metab* 3: 21 – 32

Fendt SM, Buescher JM, Rudroff F, Picotti P, Zamboni N, Sauer U (2010) Tradeoff between enzyme and metabolite efficiency maintains metabolic homeostasis upon perturbations in enzyme capacity. *Mol Syst Biol* 6: 356

Forbes SA, Beare D, Gunasekaran P, Leung K, Bindal N, Boutselakis H, Ding M, Bamford S, Cole C, Ward S *et al* (2015) COSMIC: exploring the world's knowledge of somatic mutations in human cancer. *Nucleic Acids Res* 43: D805 – D811

Fuhrer T, Heer D, Begemann B, Zamboni N (2011) High-throughput, accurate mass metabolome profiling of cellular extracts by flow injection-time-of-flight mass spectrometry. *Anal Chem* 83: 7074 – 7080

Fuhrer T, Zampieri M, Sévin DC, Sauer U, Zamboni N (2017) Genomewide landscape of gene–metabolome associations in *Escherichia coli*. *Mol Syst Biol* 13: 907

Fulda S (2013) The dark side of TRAIL signaling. *Cell Death Differ* 20: 845 – 846

Georgakopoulos-Soares I, Chartoumpekis DV, Kyriazopoulou V, Zaravinos A (2020) EMT factors and metabolic pathways in cancer. *Front Oncol* 10: 499

Ghandi M, Huang FW, Jané-Valbuena J, Kryukov GV, Lo CC, McDonald ER, Barretina J, Gelfand ET, Bielski CM, Li H *et al* (2019) Next-generation characterization of the cancer cell line encyclopedia. *Nature* 569: 503 – 508

Gonçalves E, Poulos RC, Cai Z, Barthorpe S, Manda SS, Lucas N, Beck A, Bucio-Noble D, Dausmann M, Hall C *et al* (2022) Pan-cancer proteomic map of 949 human cell lines. *Cancer Cell* 40: 835 – 849

Hackett SR, Zanotelli VRT, Xu W, Goya J, Park JO, Perlman DH, Gibney PA, Botstein D, Storey JD, Rabinowitz JD (2016) Systems-level analysis of mechanisms regulating yeast metabolic flux. *Science* 354: aaf2786

Hao Y, Baker D, ten Dijke P (2019) TGF-β-mediated epithelial-mesenchymal transition and cancer metastasis. *Int J Mol Sci* 20: 2767

Hua W, ten Dijke P, Kostidis S, Giera M, Hornsveld M (2019) TGFβ-induced metabolic reprogramming during epithelial-to-mesenchymal transition in cancer. *Cell Mol Life Sci* 77: 2103 – 2123

Jain M, Nilsson R, Sharma S, Madhusudhan N, Kitami T, Souza AL, Kafri R, Kirschner MW, Clish CB, Mootha VK (2012) Metabolite profiling identifies a key role for glycine in rapid cancer cell proliferation. *Science* 336: 1040 – 1044

Jain IH, Calvo SE, Markhard AL, Skinner OS, To T-L, Ast T, Mootha VK (2020) Genetic screen for cell fitness in high or low oxygen highlights mitochondrial and lipid metabolism. *Cell* 181: 716 – 727

Jayachandran A, Anaka M, Prithviraj P, Hudson C, McKeown SJ, Lo PH, Vella LJ, Goding CR, Cebon J, Behren A (2014) Thrombospondin 1 promotes an aggressive phenotype through epithelial-to-mesenchymal transition in human melanoma. *Oncotarget* 5: 5782 – 5797

Jeong W-J, Ro EJ, Choi K-Y (2018) Interaction between Wnt/β-catenin and RAS-ERK pathways and an anti-cancer strategy via degradations of β-catenin and RAS by targeting the Wnt/β-catenin pathway. *NPJ Precis Oncol* 2: 5

Jia D, Park JH, Kaur H, Jung KH, Yang S, Tripathi S, Galbraith M, Deng Y, Jolly MK, Kaipparettu BA *et al* (2021) Towards decoding the coupled decision-making of metabolism and epithelial-to-mesenchymal transition in cancer. *Br J Cancer* 124: 1902 – 1911

Johnson WE, Li C, Rabinovic A (2007) Adjusting batch effects in microarray expression data using empirical Bayes methods. *Biostatistics* 8: 118 – 127

Kamphorst JJ, Cross JR, Fan J, de Stanchina E, Mathew R, White EP, Thompson CB, Rabinowitz JD (2013) Hypoxic and Ras-transformed cells support growth by scavenging unsaturated fatty acids from lysophospholipids. *Proc Natl Acad Sci U S A* 110: 8882 – 8887

Kanehisa M, Goto S (2000) KEGG: kyoto encyclopedia of genes and genomes. *Nucleic Acids Res* 28: 27 – 30

Khosravi S, Wong RPC, Ardekani GS, Zhang G, Martinka M, Ong CJ, Li G (2014) Role of EIF5A2, a downstream target of Akt, in promoting melanoma cell invasion. *Br J Cancer* 110: 399 – 408

Kuligowski J, Sánchez-Illana Á, Sanjuán-Herráez D, Vento M, Quintás G (2015) Intra-batch effect correction in liquid chromatography-mass spectrometry using quality control samples and support vector regression (QC-SVRC). *Analyst* 140: 7810 – 7817

Lagziel S, Gottlieb E, Shlomi T (2020) Mind your media. *Nat Metab* 2: 1369 – 1372

Li H, Ning S, Ghandi M, Kryukov GV, Gopal S, Deik A, Souza A, Pierce K, Keskula P, Hernandez D *et al* (2019a) The landscape of cancer cell line metabolism. *Nat Med* 25: 850 – 860

Li S, Zhang Z, Han L (2019b) Molecular treasures of cancer cell lines. *Trends Mol Med* 25: 657 – 659

McQuin C, Goodman A, Chernyshev V, Kamentsky L, Cimini BA, Karhohs KW, Doan M, Ding L, Rafelski SM, Thirstrup D *et al* (2018) CellProfiler 3.0: next-generation image processing for biology. *PLoS Biol* 16: e2005970

Meyers RM, Bryan JG, McFarland JM, Weir BA, Sizemore AE, Xu H, Dharia NV, Montgomery PG, Cowley GS, Pantel S *et al* (2017) Computational correction of copy number effect improves specificity of CRISPR-Cas9 essentiality screens in cancer cells. *Nat Genet* 49: 1779 – 1784

 © 2022 The Authors

Nusinow DP, Szpyt J, Ghandi M, Rose CM, McDonald ER, Kalocsay M, Jané-Valbuena J, Gelfand E, Schweppe DK, Jedrychowski M *et al* (2020) Quantitative proteomics of the cancer cell line encyclopedia. *Cell* 180: 387 – 402

Ortmayr K, Dubuis S, Zampieri M (2019) Metabolic profiling of cancer cells reveals genome-wide crosstalk between transcriptional regulators and metabolism. *Nat Commun* 10: 1841

Park JO, Rubin SA, Xu Y-F, Amador-Noguez D, Fan J, Shlomi T, Rabinowitz JD (2016) Metabolite concentrations, fluxes and free energies imply efficient enzyme usage. *Nat Chem Biol* 12: 482 – 489

Pavlova NN, Thompson CB, Almuhaideb A, Papathanasiou N, Bomanji J, Astuti D, Latif F, Dallol A, Dahia PL, Douglas F *et al* (2016) The emerging hallmarks of cancer metabolism. *Cell Metab* 23: 27 – 47

Rajapakse VN, Luna A, Yamade M, Loman L, Varma S, Sunshine M, Iorio F, Sousa FG, Elloumi F, Aladjem MI *et al* (2018) CellMinerCDB for integrative cross-database genomics and pharmacogenomics analyses of cancer cell lines. *iScience* 10: 247 – 264

Ribeiro AS, Paredes J (2015) P-cadherin linking breast cancer stem cells and invasion: a promising marker to identify an 'intermediate/metastable' EMT state. *Front Oncol* 4: 371

Risso D, Ngai J, Speed TP, Dudoit S (2014) Normalization of RNA-seq data using factor analysis of control genes or samples. *Nat Biotechnol* 32: 896 – 902

Schubert M, Klinger B, Klünemann M, Sieber A, Uhlitz F, Sauer S, Garnett MJ, Blüthgen N, Saez-Rodriguez J (2018) Perturbation-response genes reveal signaling footprints in cancer gene expression. *Nat Commun* 9: 20

Segura-Lepe MP, Keun HC, Ebbels TMD (2019) Predictive modelling using pathway scores: robustness and significance of pathway collections. *BMC Bioinformatics* 20: 543

Semenza GL (2012) Hypoxia-inducible factors in physiology and medicine. *Cell* 148: 399 – 408

Shoemaker RH (2006) The NCI60 human tumour cell line anticancer drug screen. *Nat Rev Cancer* 6: 813 – 823

Shorthouse D, Bradley J, Critchlow SD, Bendtsen C, Hall BA (2022) Heterogeneity of the cancer cell line metabolic landscape. *Mol Syst Biol* 18: e11006

Somerville TDD, Xu Y, Miyabayashi K, Tiriac H, Cleary CR, Maia-Silva D, Milazzo JP, Tuveson DA, Vakoc CR (2018) TP63-mediated enhancer reprogramming drives the squamous subtype of pancreatic ductal adenocarcinoma. *Cell Rep* 25: 1741 – 1755

Son J, Lyssiotis CA, Ying H, Wang X, Hua S, Ligorio M, Perera RM, Ferrone CR, Mullarky E, Shyh-Chang N *et al* (2013) Glutamine supports pancreatic cancer growth through a KRAS-regulated metabolic pathway. *Nature* 496: 101 – 105

Storey JD, Tibshirani R (2003) Statistical significance for genomewide studies. *Proc Natl Acad Sci U S A* 100: 9440 – 9445

Subramanian A, Tamayo P, Mootha VK, Mukherjee S, Ebert BL (2005) Gene set enrichment analysis: a knowledge-based approach for interpreting genome-wide. *Proc Natl Acad Sci U S A* 43: 15545 – 15550

Szklarczyk D, Morris JH, Cook H, Kuhn M, Wyder S, Simonovic M, Santos A, Doncheva NT, Roth A, Bork P *et al* (2017) The STRING database in 2017: quality-controlled protein-protein association networks, made broadly accessible. *Nucleic Acids Res* 45: D362 – D368

Tsherniak A, Vazquez F, Montgomery PG, Weir BA, Kryukov G, Cowley GS, Gill S, Harrington WF, Pantel S, Krill-Burger JM *et al* (2017) Defining a cancer dependency map. *Cell* 170: 564 – 576

Warburg O, Minami S (1923) Versuche an überlebendem carcinom-gewebe. *Klin Wochenschr* 2: 776 – 777

Ward PS, Thompson CB (2012) Metabolic reprogramming: a cancer hallmark even warburg did not anticipate. *Cancer Cell* 21: 297 – 308

Wehrens R, JosA H, van Eeuwijk F, Kooke R, Flood PJ, Wijnker E, Keurentjes JJB, Lommen A, van Eekelen HDLM, Hall RD *et al* (2016) Improved batch correction in untargeted MS-based metabolomics. *Metabolomics* 12: 88

Wigerup C, Påhlman S, Bexell D (2016) Therapeutic targeting of hypoxia and hypoxia-inducible factors in cancer. *Pharmacol Ther* 164: 152 – 169

Wishart DS, Jewison T, Guo AC, Wilson M, Knox C, Liu Y, Djoumbou Y, Mandal R, Aziat F, Dong E *et al* (2013) HMDB 3.0–the human metabolome database in 2013. *Nucleic Acids Res* 41: D801 – D807

Yeh HW, Lee SS, Chang CY, Lang YD, Jou YS (2019) A new switch for TGFβ in cancer. *Cancer Res* 79: 3797 – 3805

Zhu W, Cai MY, Tong ZT, Dong SS, Mai SJ, Liao YJ, Bian XW, Lin MC, Kung HF, Zeng YX *et al* (2012) Overexpression of EIF5A2 promotes colorectal carcinoma cell aggressiveness by upregulating MTA1 through C-myc to induce epithelial – mesenchymaltransition. *Gut* 61: 562 – 575

License: This is an open access article under the terms of the Creative Commons Attribution License, which permits use, distribution and reproduction in any medium, provided the original work is properly cited.

Expanded View Figures

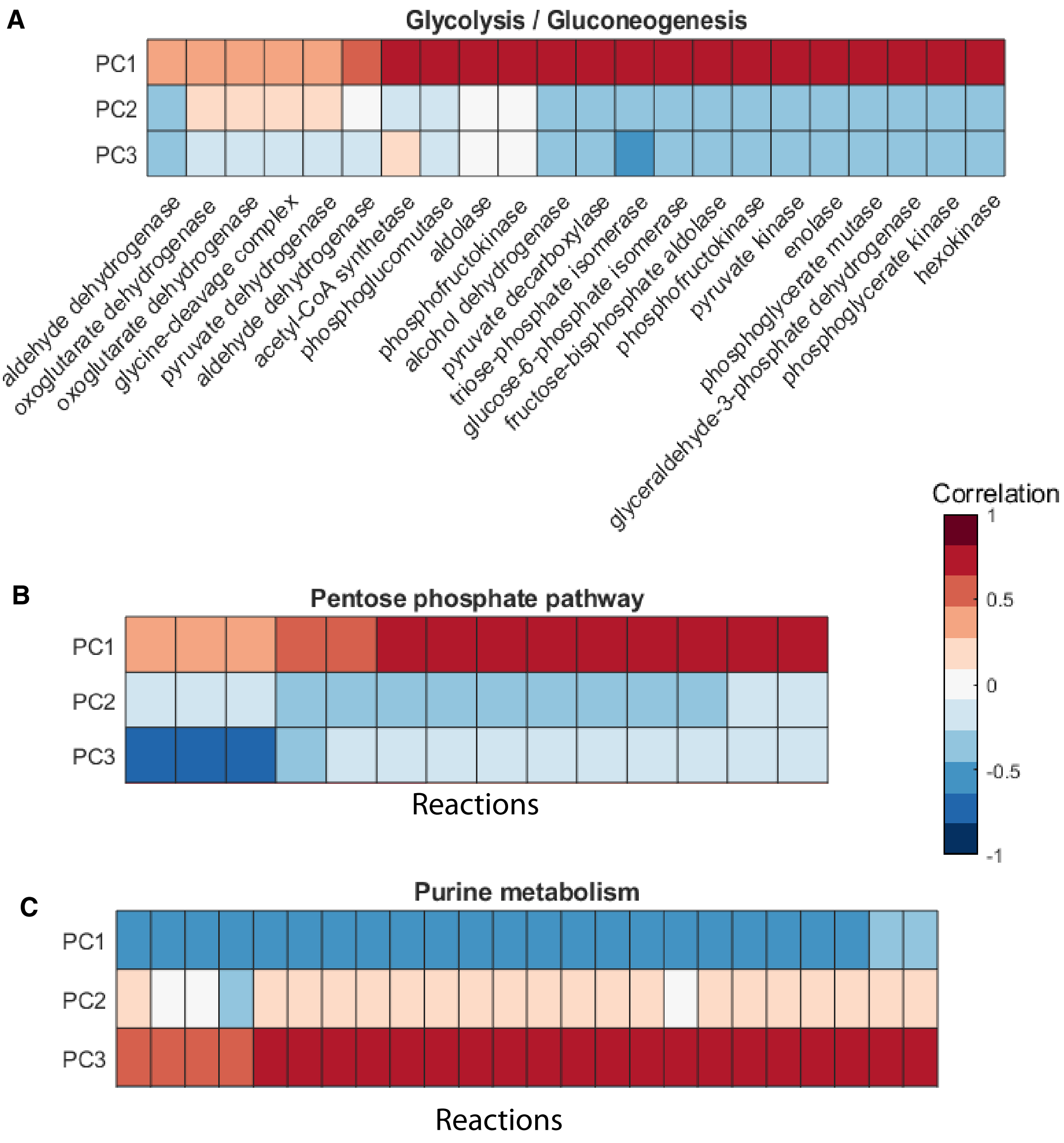

Figure EV1. Pathway activity scoring reveals link between metabolites levels and fluxes.

A–C Pearson correlation between metabolites levels factorized into principal components and fluxes of a pathway. Fluxes and metabolites of (A) glycolysis, (B) pentose phosphate pathway, and (C) purine metabolism. Data taken from Hackett *et al* (2016). PC1 is used as pathway activity score.

 © 2022 The Authors

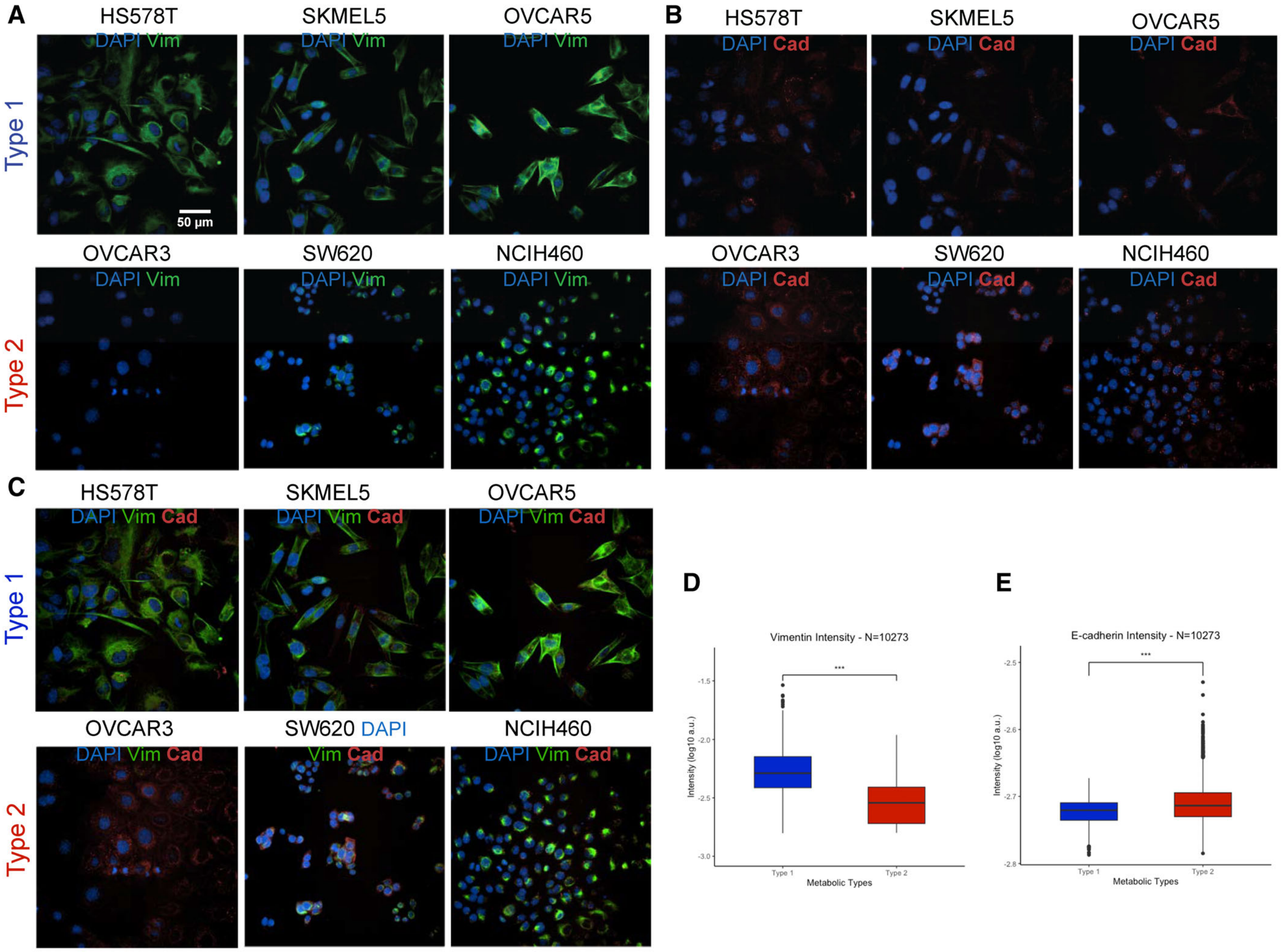

Figure EV2. Relation between metabolic types and epithelial/mesenchymal state.

A–C Metabolic types stained for the nucleus (blue), (A) for vimentin (green), and (B) e-cadherin (red), (C) both, two markers of EMT. Scaling bar in (A) applies to all images.

D, E Quantification of the expression of (D) vimentin and (E) e-cadherin of all segmented cells (number of cells $n = 10,273$, boxplot depicts first quartile, median, and third quartile, two-sided unpaired student t-test). ***$P \leq 0.001$. Abbreviation: a.u. arbitrary units.

Figure EV3. Difference in lipids unsaturation.

A, B Lipid unsaturation, concentration-weighted average double per acyl chain, across metabolic types (cell lines $n = 7$ with three biological replicates per cell line and two technical replicates, boxplot depicts first quartile, median, and third quartile, two-sided unpaired student t-test) for (A) each lipid class and (B) for all lipids. ns: $P > 0.05$, *$P < 0.05$, **$P < 0.01$, ***$P < 0.001$, ****$P < 0.0001$. Abbreviation: cardiolipins (CL), ether phosphatidylcholines (ether PC), hexosyl-ceramide (HexCer), lysophosphatidylcholines (LPC), lysophosphatidylethanolamine (LPE), phosphatidylethanolamine (PE), phosphatidylserine (PS), phosphatidylcholines (PC), sphingomyelin (SM), and triacylglycerols (TAG).

© 2022 The Authors

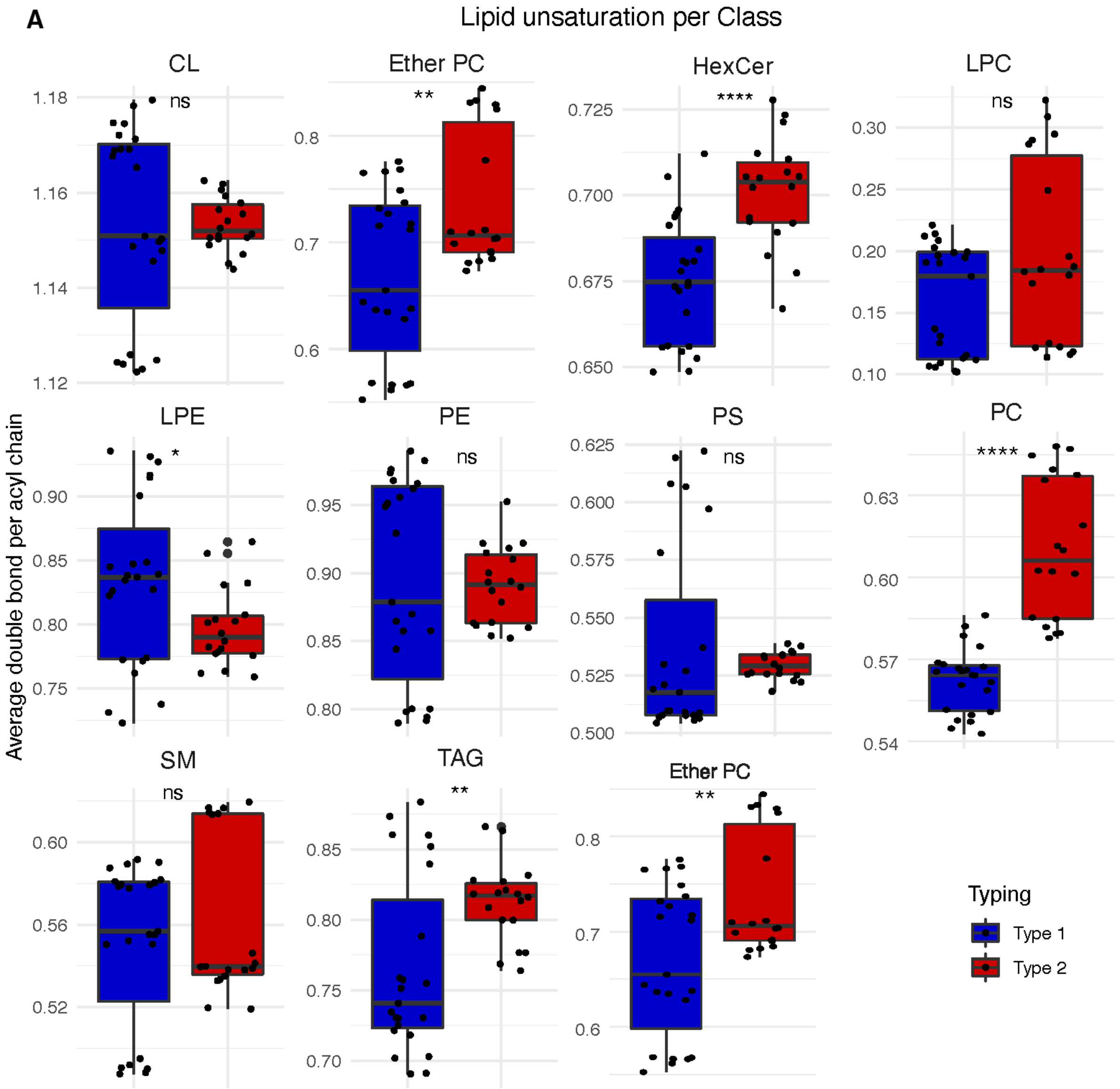

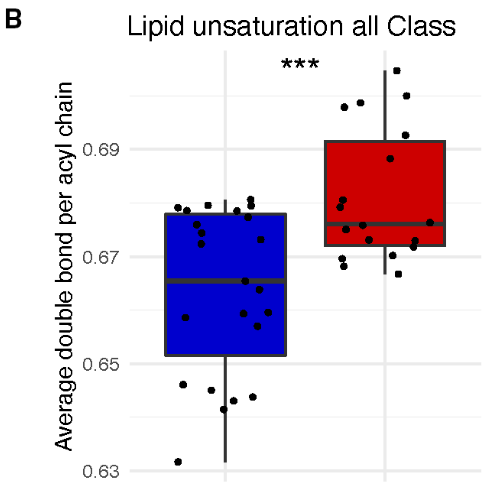

Figure EV3.

 © 2022 The Authors

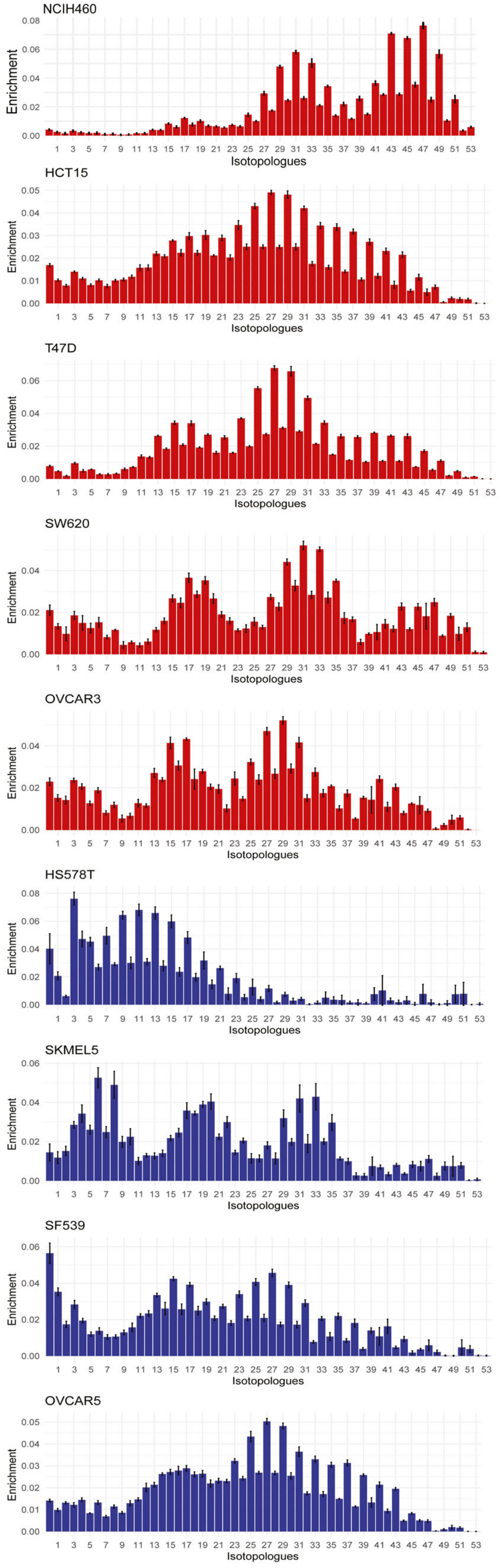

Figure EV4. Mass distribution vector of TAG 50:1 labeled from [U-^{13}C] glucose for all followed up cell lines (cell lines *n* = 9, with three biological replicates per cell line and two technical replicates, mean ± standard deviation).

© 2022 The Authors

Appendix to:

A functional analysis of 180 cancer cell lines reveals conserved intrinsic metabolic programs

Sarah Cherkaoui [1,2], Stephan Durot [1,2], Jenna Bradley [3], Susan Critchlow [3], Sebastien Dubuis [1], Mauro Miguel Masiero [1,2], Rebekka Wegmann [1,2], Berend Snijder [1], Alaa Othman [1,4], Claus Bendtsen [3], Nicola Zamboni [1, 4]

[1] Institute of Molecular Systems Biology, ETH Zürich, Otto-Stern-Weg 3, 8093 Zürich, Switzerland

[2] PhD Program in Systems Biology, Life Science Zürich, 8057 Zürich, Switzerland

[3] AstraZeneca, R&D, 310 Milton Road, CB4 0WG Cambridge, United Kingdom

[4] PHRT Swiss Multi-OMICS Center / smoc.ethz.ch, Switzerland

Table of Content:

Appendix Figures S1-7

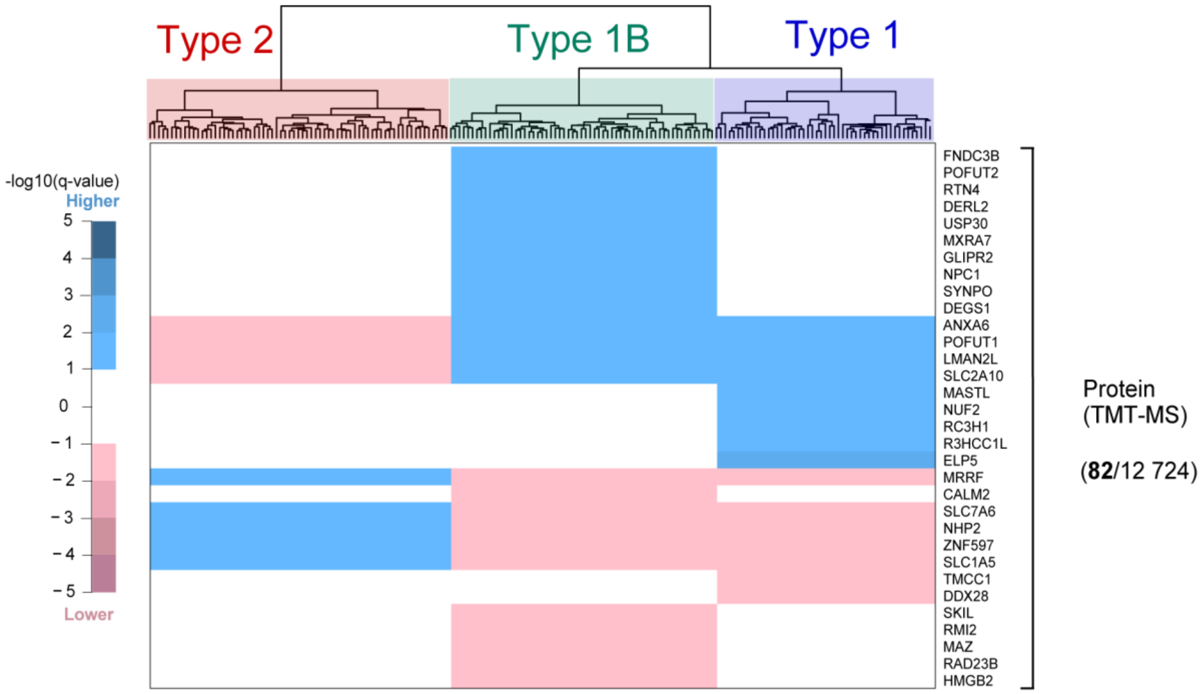

Appendix Figure S1. The proteins associated to metabolic activity phenotypes. Summary of significant associations between metabolic types and proteins levels from Tandem Mass Tag Mass Spectrometry (TMT-MS) dataset. The list and number of traits integrated to the metabolic phenotype are listed on the right panel, where traits were considered significant at 10% FDR (cell lines n=180). For visualization, q-values were extended with a sign to indicate whether the trait is significantly higher or lower in the compared to the rest of the tree. Only traits significantly associated to the tree main types (1, 2, and 1B) are shown. Full results are reported in data availability.

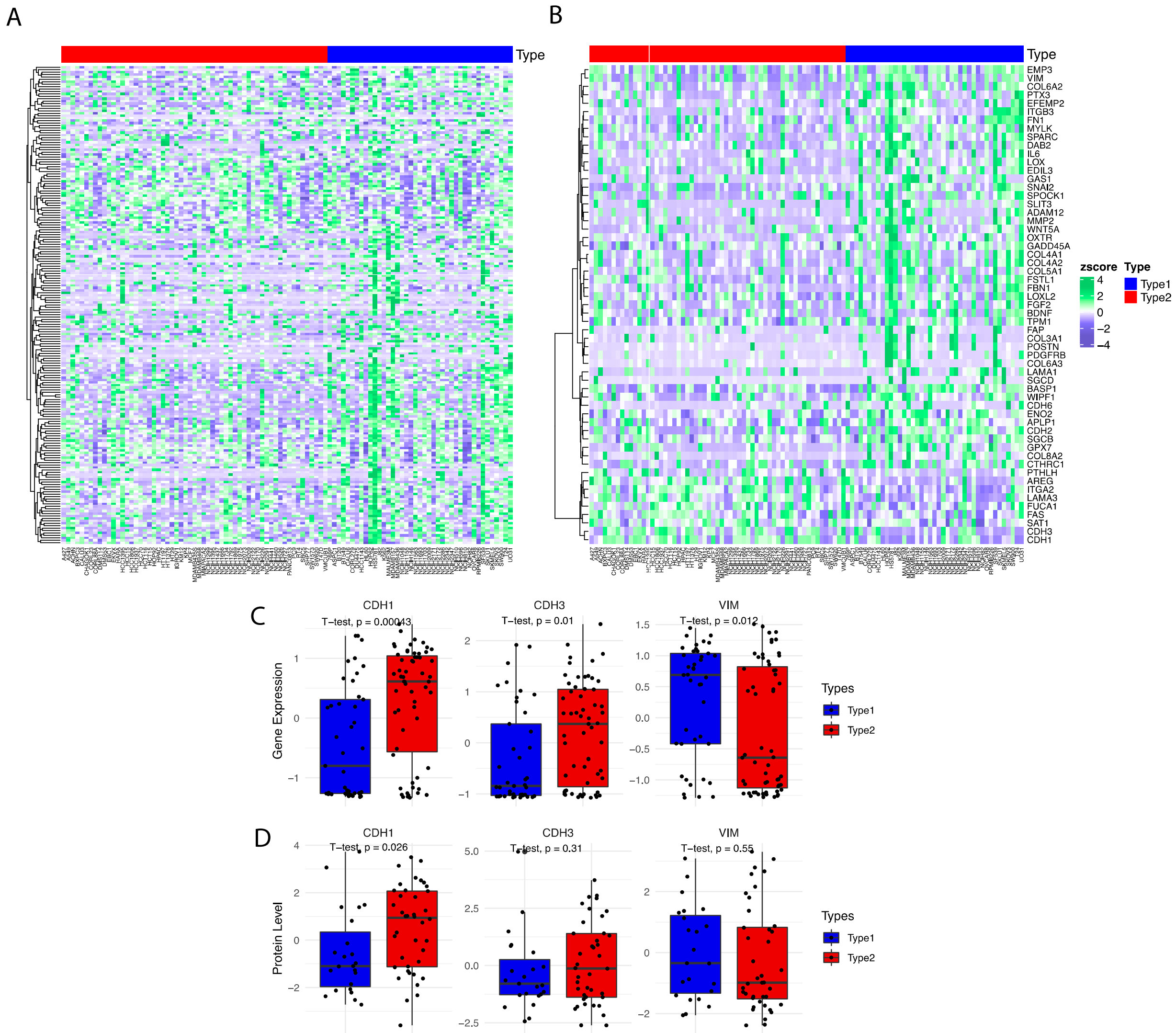

Appendix Figure S2. EMT gene expression signature across types. A. Gene expression EMT in the two metabolic types, where gene set was taken from MSigDB (GSEA). B. Expression of 57 significatively changing EMT genes (out of 197) between type 1 and 2. Comparison of genes expression and protein levels of specific EMT genes, cadherin and vimentin. C. Gene expression data taken from CCLE. D. Protein levels taken from Tandem Mass Tag Mass Spectrometry (TMT-MS) dataset (Nusinow et al.). Data information: Each dot depicts a cell line. Boxplot depicts first quartile, median and third quartile, two-sided unpaired student t-test.

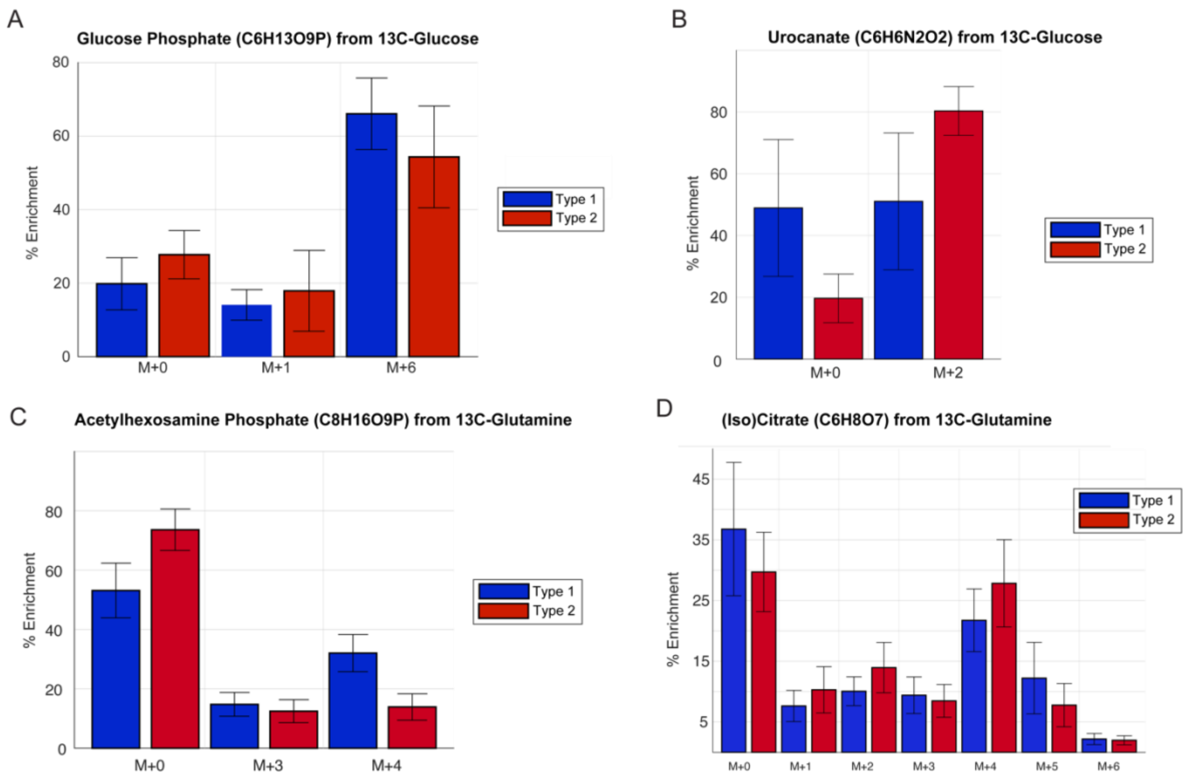

Appendix Figure S3. Mass distribution vector of changing metabolites between metabolic types. A. Glucose phosphate labeled from [U-13C]glucose. B. Urocanate labeled from [U-13C]glucose. C. Acetylhexosamine phosphate labeled from [U-13C]glutamine. D. (Iso)Citrate labeled from [U-13C]glutamine.

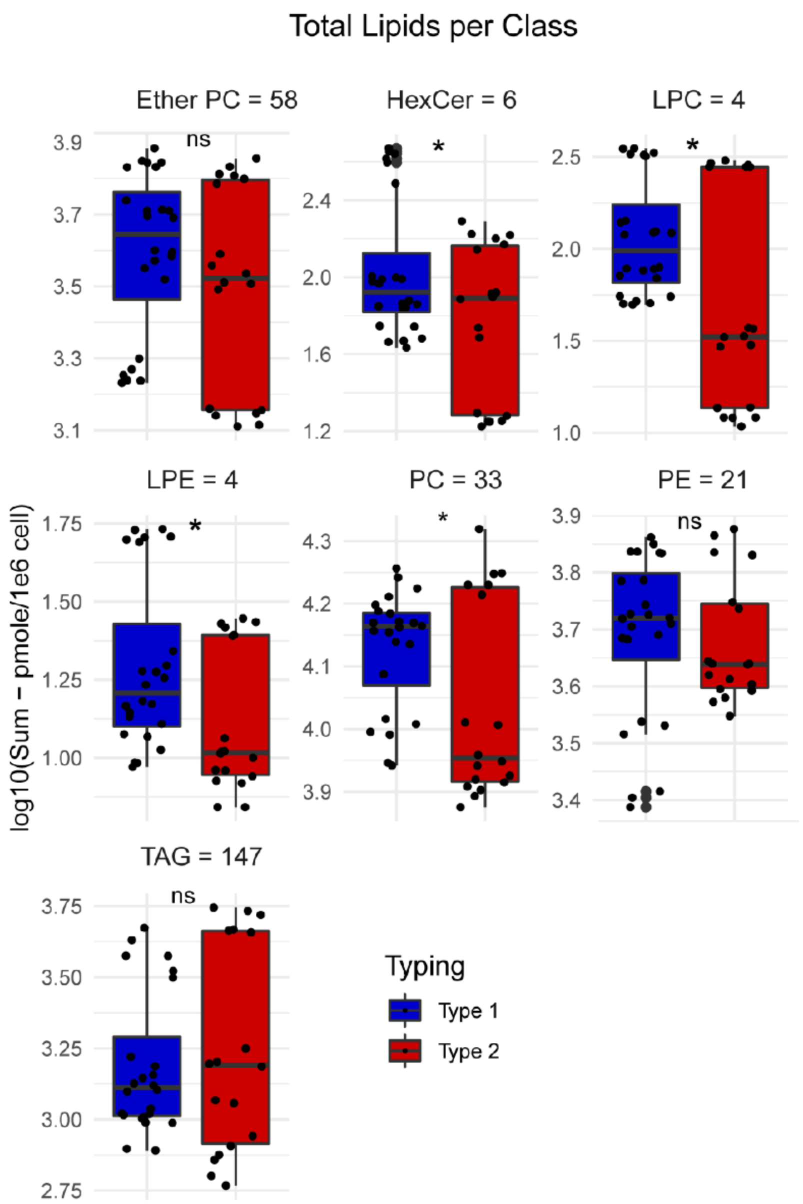

Appendix Figure S4. Differences in lipid content of individual class, with number of lipids per class in title (cell lines n=7, Student t-test). ns: p > 0.05, *: p <= 0.05, **: p <= 0.01, ***: p <= 0.001, ****: p <= 0.0001. Abbreviation: ether phosphatidylcholines (ether PC), hexosyl-ceramide (HexCer), lysophosphatidylcholines (LPC), lysophosphatidylethanolamine (LPE), phosphatidylcholines (PC), phosphatidylethanolamine (PE), sphingomyelin (SM), and triacylglycerols (TAG).

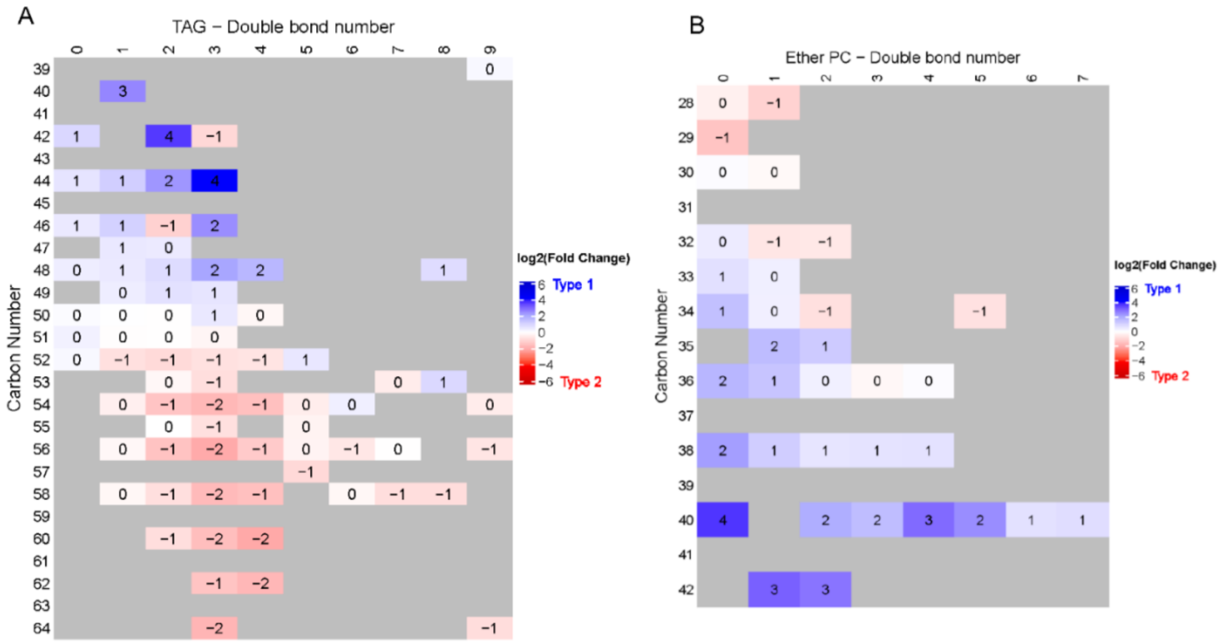

Appendix Figure S5. Comparison of lipid species by double bon and carbon number. Differential analysis of A. TAG and B. Ether PC lipid species displayed per double bond number and carbon number. Abbreviation: triacylglycerols (TAG) and ether phosphatidylcholines (ether PC).

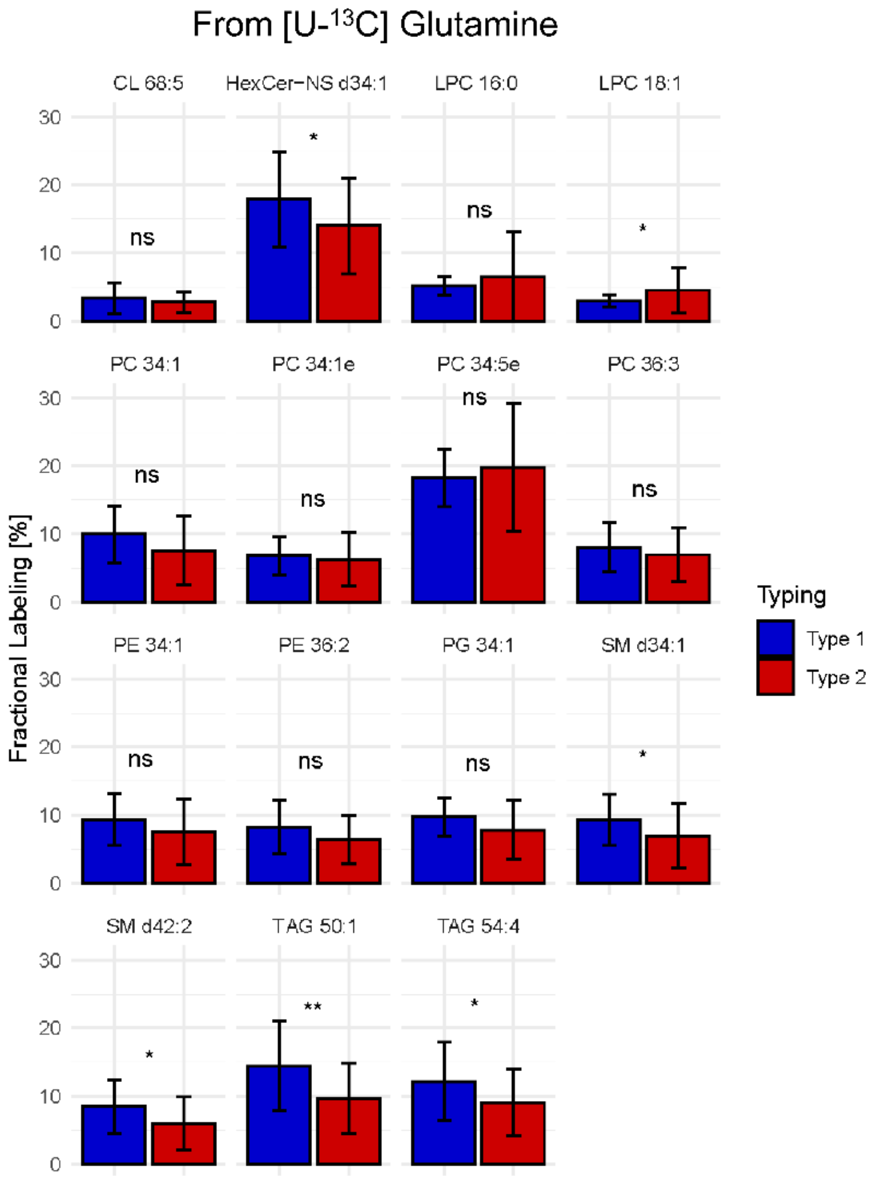

Appendix Figure S6. Differential *de novo* lipid biosynthesis from [U-^{13}C]glutamine. Bar plot (mean ± standard deviation) of fractional labeling contribution from [U-^{13}C]glutamine of the most abundant lipids per lipid class (cell lines n=9, Student t-test). ns: p > 0.05, *: p <= 0.05, **: p <= 0.01, ***: p <= 0.001, ****: p <= 0.0001. Abbreviation: cardiolipins (CL), hexosyl-ceramide (HexCer), lysophosphatidylcholines (LPC), phosphatidylcholines (PC), phosphatidylethanolamine (PE), phosphatidylglycerols (PG), sphingomyelin (SM), and triacylglycerols (TAG).

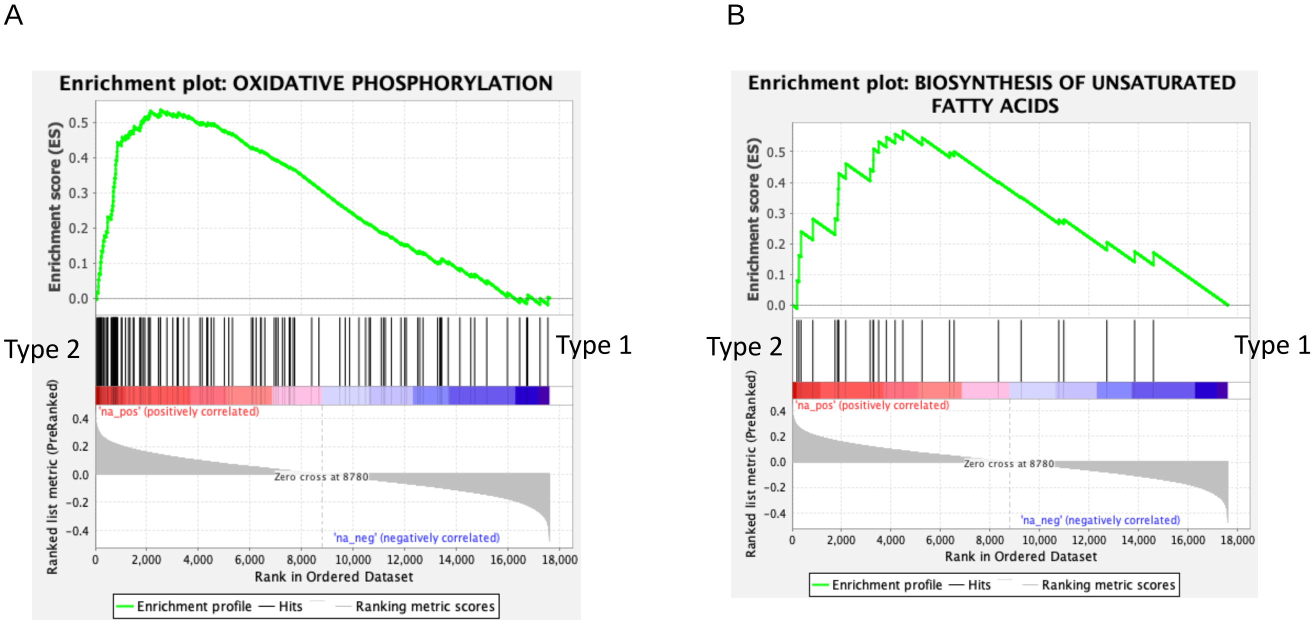

Appendix Figure S7. Pathway dependency of metabolic types. A. Results of gene set enrichment of oxidative phosphorylation and B. biosynthesis of unsaturated fatty acids. Gene set enrichment analysis results and figures were generated using leading edge analysis from GSEA (https://www.gsea-msigdb.org/gsea/index.jsp).
