# Supplementary material for: A functional analysis of 180 cancer cell lines reveals conserved intrinsic metabolic programs
Source: Mol Syst Biol. 2022 Nov 2;18(11):e11033. doi: 10.15252/msb.202211033 (PMC9627673; doi:10.15252/msb.202211033)
Supplement: Supplementary file 7 [file MSB-18-e11033-s001.pdf]

molecular systems biology

# A functional analysis of 180 cancer cell lines reveals conserved intrinsic metabolic programs

Sarah Cherkaoui, Stephan Durot, Jenna Bradley, Susan Critchlow, Sebastien Dubuis, Mauro Masiero, Rebekka Wegmann, Berend Snijder, Alaa Othman, Claus Bendtsen, and Nicola Zamboni
**DOI: 10.15252/msb.202211033**

*Corresponding author(s): Nicola Zamboni (zamboni@imsb.biol.ethz.ch)*

## Review Timeline:

| | |
|---|---|
| Submission Date: | 17th Mar 22 |
| Editorial Decision: | 22nd Apr 22 |
| Revision Received: | 20th Jul 22 |
| Editorial Decision: | 16th Aug 22 |
| Revision Received: | 3rd Oct 22 |
| Accepted: | 7th Oct 22 |

*Editor: Jingyi Hou*

## Transaction Report:

(Note: With the exception of the correction of typographical or spelling errors that could be a source of ambiguity, letters and reports are not edited. Depending on transfer agreements, referee reports obtained elsewhere may or may not be included in this compilation. Referee reports are anonymous unless the Referee chooses to sign their reports.)

Thank you for submitting your work to Molecular Systems Biology. We have now heard back from the three reviewers who agreed to evaluate your manuscript. As you will see from the reports below, the reviewers acknowledge the potential interest of the study and appreciate the resource value of the metabolomics data provided. They raise, however, a series of overlapping concerns, which should be addressed in a major revision of the current manuscript.

Without reiterating all the points raised in the reviews below, some of the more substantial issues are the following:

- As indicated by all three reviewers, a comparison with existing metabolomics data should be performed to assess the reproducibility of the presented data.
- The reviewers' concerns about metabolic pathway analysis, statistics, and several potential confounding factors in the data analysis need to be considered and carefully addressed.
- In light of Reviewer #1's comment, additional details about the data normalization pipeline should be provided, and the data processing differences between the two studies (if any) should be clearly stated in the Materials and Methods section.

Other issues raised by the reviewers need to be satisfactorily addressed as well. As you may already know, our editorial policy allows, in principle, a single round of major revision, and acceptance or rejection of the manuscript will depend on another round of review, it is therefore essential to provide responses to the reviewers' comments that are as complete as possible.

On a more editorial level, we would ask you to address the following issues:

--------------------------------------------------------

Reviewer #1:

In the present manuscript, Cherkaoui et al investigate the landscape of metabolic profiles in a large panel of cancer cell lines. Towards this end, the authors use untargeted metabolomics to quantify the metabolic profile of 180 cancer cell lines grown under identical conditions. Using this metabolic data set together with follow-up experiments (13C labeling, lipidomics, and imaging), the authors show that this cell line panel broadly falls into two distinct metabolic groups, which are strongly associated with their EMT status.
Overall, the manuscript is clearly presented and well written, and the metabolomics data generated here will surely be useful to the broader field of cancer metabolism. I particularly appreciate the authors' efforts to develop an appropriate data normalization strategy (which is a big issue when working with such large metabolomics data sets), and the follow-up experiments (13C-labeling, lipidomics, EMT validation). Therefore, I believe this manuscript will be of interest for the readership of MSB. However, there are currently several issues that should be clarified in a revised manuscript.

Main comments:

1. Relationship to previous literature: The authors' claim that "no systematic characterization of metabolic-wide dysregulation has been accessed to date" (lines 76-77) is unclear to me. As the authors point out themselves, this is not the first attempt to comprehensively access the metabolic state of diverse cancer cell lines. For example, recent works have covered a larger cell panel, but with fewer metabolites measured (PMID 31068703), or a smaller cell panel (~60 cell lines), but with a similar number of measured metabolites (PMID 31015463). Currently the "added value" this study provides to the field remains somewhat unclear, since the authors make little effort to put their findings in the broader context of the recent literature (the same criticism applies to both back-to-back manuscripts). For example, what made the key finding of this study (i.e. two main metabolic types that are strongly associated with EMT) possible: was is simply the scale of the present study, or the focus on pathways rather than individual metabolites? Would this association have emerged already in the previous studies if they had used the same method to determine metabolic pathway activity? I believe that this manuscript would benefit from an additional paragraph e.g. in the discussion tackling this point.

2. Lacking detail on EMT validation: I appreciate the authors' efforts to validate the EMT status of selected cell lines with imaging, but currently the description of the methods used is very sparse. For example, I assume the cells were fixed prior to staining, but the method sections (starting in line 544) does not mention anything along these lines. Also, what exactly was the blocking solution the authors used? Please include the necessary information to enable other researchers to reproduce the results.

3. "Empirical" pathway score: I was very intrigued by the authors' approach to quantify a "pathway activity score" based on principal component analysis. Given that this score is central for most downstream analyses performed in this study, I wonder whether the authors have performed any additional sanity checks (other than the analysis of a previously published yeast data set). For example, how does this metric compare to alternative approaches to quantify metabolic pathway activity, e.g. fraction of significantly altered metabolites?

4. Interpretation of key finding: A key finding of this paper is that the tested cancer cell lines fall into two broad metabolic groups, which are characterized with generally higher/lower pathway activity and 13C-glucose labeling (type 2 and type 1, respectively), and which are associated with an epithelial or mesenchymal cell state, respectively. This is a striking observation, and I am wondering what the underlying process may be. In my understanding, the generally higher pathway activities and especially the higher 13C incorporation suggest a difference in metabolic turn over. Do these types differ in their growth rate, or in their rate of

e.g. protein turn over? Do these metabolic patterns match what we know about EMT outside of cancer, e.g. in development? I believe that this manuscript may benefit from an additional paragraph discussing these points.

5. Along the same lines, I am wondering about the causality here: does EMT state determine the metabolic state, or the other way around? The authors already talk about this point in their discussion, but I am wondering whether there is an (experimental) way to test the different possibilities, e.g. using drugs that modulate EMT: would shifting a cell line pharmacological from a epithelial to mesenchymal state also alter its metabolic profile in the expected direction? I understand that testing such conjectures experimentally is outside the scope of this manuscript, but I encourage the authors to at least discuss potential validation experiments in a revised manuscript.

6. Lacking "harmonization" of the metabolomics data sets in the two back-to-back manuscripts: It is my understanding that this manuscript is part of a back-to-back submission with another manuscript (MSB-2022-11006), which focuses on elucidating the metabolic differences between these cancer cell lines using the metabolomics data set generated here. However, it is unclear to me why these two manuscripts seem to use quite different data normalization methods, in which not only the number of ions considered, but also the number of cell lines are different. As I mentioned in my report of the other manuscript, I strongly suggest that the authors (of both manuscripts) either harmonize their data normalization pipelines or explain in detail why different ones are being used. Otherwise, I worry that having two conflicting data sets based on the same original data published back-to-back will lead to substantial confusion in the field.

Additional comments:
1. Lines 93-94: How did the authors select this panel of cancer cell lines? Was there any specific motivation to select half of them from the same tissue (lung)?

2. Lines 240-241: Please add a pointer to the respective 13C-glutamine labeling data in the text (I think its Figure S6).

Reviewer #2:

The paper from Cherkaoui et al. presents a potentially incredibly useful data resource. They generated untargeted metabolomic profiling data entailing 1809 ions for 180 cancer cell lines. Interestingly, the authors identified only two distinct metabolic types. They performed subsequent 13C-flux analysis, lipidomics, and association with gene essentiality data to explore the differences between the two metabolic subsets.

Major points:

1. While there is only one metabolite name in the "Top.annotation.name", multiple KEGG and HMDB IDs are provided in the Top.annotation.ids". Please describe how was the "top" chosen. Among the isobaric metabolites, which was actually used for the pathway score calculation? If the current analysis includes multiple isobaric metabolites per ion for pathway analysis, wouldn't the pathway score be inflated as an artifact? Can the authors show how would the results be affected if only one most abundant representative metabolite is picked for the ion?
2. In Figure 5C, the jittered points seem to form subclusters. It seems multiple replicates of the same cell lines were included in the analysis. The author should use a mixed-effect model rather than t-test because the inter-replicate variation is much smaller than the inter-cell line variation. Preferably, different colors should be used to color for different cell lines so replicates from the same cell lines will be in the same color.
3. I would like to see a comparison (distribution of metabolite-specific pairwise correlations across shared cell lines for all the shared metabolites) with the existing CCLE metabolomics data to see the cross-study reproducibility of cell line metabolomics data.
4. H460 has previously been characterized as a mesenchymal cell line (PMID 23091115). Here the authors have assigned it to epithelial despite finding plenty of vimentin expression in Figure S2. In Figure 5D, they compared a breast cancer cell line HS578T to H460 although they mentioned they have selected a pair of breast cancer cell line (T47D being the mesenchymal counterpart) in order to compare by lineage. Please show T47D instead of H460. Can the authors use an EMT geneset to show that cell lines from the two types actually separate by their gene expression on a heatmap?

Minor points:
1. Legends like the one in Fig 3 can be quite confusing. I suggest the authors break it down into two sublegends, one in grayscale and use different shades to represent different magnitudes of -log10(p-value), the other using color to represent direction.
2. Try to derive the combined functional loss status for AXIN1, CDKN2b by integrating mutation, copy number, and RPPA data to see if stronger associations can be found.
3. Please provide Cellosaurus RRID for the cell lines in supplementary data
4. Please deposit reproducible codes used for the analyses.
5. Please provide processed 13C labeling metabolomics and lipidomics data in a separate folder so users don't need to download the whole large zip files that contain the raw data in order to access the processed data.
6. It may not be intuitive for a general reader to know how fractional contribution is derived, please provide a formula or citation. It seems from their processed data, for the majority of the metabolites, the full set of isotopologues (m+0, m+1, ..., m+n) is not available. What is the reason for such missing data, undetectable signal? Do the authors assume these missing isotopologues as zero values when they performed the analyses shown in Fig 4?
7. Figures 3 and 4 are high-level summary figures, can the authors provide some concrete examples (like 5D, but for all cell lines) as supplementary figures?
8. Did the authors aggregate their replicate data to cell line-level data (for example, by taking the mean or median) for their analyses? If so, please provide this processed data in the repo too.

Reviewer #3:

Cherkaoui et al. present one of the largest metabolic characterization of cancer cell lines to-date, quantifying using untargeted metabolomics over 1,800 metabolic measurements (deprotonated metabolites) across 180 cancer cell lines. This rich resource is used to estimate the activity of 49 metabolic pathways and assess their heterogeneity across cancers. Surprisingly, the authors conclude that cancer cell lines group only into two major clusters, which they relate to other publicly available multi-omics datasets for biomarker association analysis, and experimentally assess / validate their metabolic profiles using 13-C metabolomics, revealing an association with the epithelial to mesenchymal transition (EMT).

In general, the manuscript is very well written and results are well presented, together with the metabolomics resource I am confident this will be of great interest to the broad scientific community with immediate applications to cancer translation (e.g. biomarker discovery). It also expands similar studies - Li et al. (2019) quantified 225 metabolites across 928 cell lines - particularly on the number of metabolic readouts. The main conclusions of the manuscript are interesting and in line with recent cancer cell line multi-omics studies. Nonetheless, further clarifications are needed on the technological and methodological aspects (e.g. metabolic pathway activity score, lack of genomic associations, cancer cell lines diverse growth rates) to understand if these might not impact the conclusions. The sole focus of the downstream analyses on the metabolic pathway activity scores might have limited the exploration of the richness of this data, as only a portion (20%) of the data is used to calculate these scores

Major points:

1. Can the authors comment on the considerable reduction of the metabolic measurements when only considering those that map to KEGG pathways? If I understand correctly, only 367 annotated ions could be mapped (~20% of all quantified metabolic readouts). Also, the pathway activity scores are mainly derived from PC1, would this mean that metabolic pathway activities will be mostly related to the main driver of variability in the metabolomics and miss other likely interesting sources of variability? Indeed, it would be expected that the first PCs would be associated with EMT as shown by other orthogonal omics (Li et al. 2017, Rajapakse et al. 2018, Gonçalves et al. 2022).

2. Related to my previous main point, the clustering of the cancer cell lines into two major groups is indeed interesting. Considering this conclusion relies on the pathway activity scores, could the authors comment if the cancer cell lines hierarchical clustering and conclusions are preserved when calculated using the total measured ions (1,809), i.e. if other cancer cell lines sub-clusters start to emerge (as it seems the case from Figure 2 and 3)? Also, can the authors comment on how their findings compare with the cell line grouping achieved from the independent metabolic dataset Li et al. (2019)?

3. Presumably this resource has overlapping cell lines with the independent metabolic map presented by Li et al. (2019), and therefore it would be important to understand how these datasets compare to assess reproducibility. For example, could the authors correlate the metabolic profiles (e.g. pathway activity scores, metabolic measurements or other metrics) of the same cancer cell line in both datasets and how these compared to the random expectation (i.e. all-vs-all)?

4. Cancer cell lines can be very heterogeneous (e.g. tissue-type, growth rate, cell size, etc) and this becomes an important consideration for large-scale studies, such as this, to avoid potential confounding factors. The authors have already taken an important step towards this by considering similar culture conditions. Have the authors corrected or assessed the potential impact of cell lines' divergent growth rates on the metabolic measurements? Also, could the authors comment on the potential impact of cell size (with regards to its relation with tumor invasion and EMT)?

5. The lack of genomic associations is surprising. Could this be a consequence of using the pathway activity scores? Would a systematic association with deprotonated ions expand the potential for identifying associations? Perhaps pathway level analysis could be carried after the association analysis using the effect sizes?

6. For the proteomics analysis the authors focused on reverse-phase protein array (RPPA), whilst this has some very specific advantages (e.g. phospho antibodies) a more systematic protein measurement based on TMT-MS is now available quantifying the proteomes (>10,000 proteins) of 375 cancer cell lines (Nusinow et al. 2020). It would be worth expanding the current analysis with this dataset. For example, this would give insights into other canonical markers of EMT, such as VIM.

Minor points:

1. From a PCA analysis of both the pathway activity and metabolite ions what is the percentage of variance captured across the top PCs? Are multiple PCs associated with EMT?

2. Figure 6B IDH2 and SDHAF4 CRISPR-Cas9 gene essentiality scores are overall very low, whilst the mean difference is significant these genes are likely non-essential across all cell lines. SDHAF4 does show some dependency in a small number of cancer cell lines, from the DepMap portal these are enriched for melanoma cancer cell lines.

## Point-by-point answer to Reviewer's comments

Reviewer #1:

In the present manuscript, Cherkaoui et al investigate the landscape of metabolic profiles in a large panel of cancer cell lines. Towards this end, the authors use untargeted metabolomics to quantify the metabolic profile of 180 cancer cell lines grown under identical conditions. Using this metabolic data set together with follow-up experiments (13C labeling, lipidomics, and imaging), the authors show that this cell line panel broadly falls into two distinct metabolic groups, which are strongly associated with their EMT status.
Overall, the manuscript is clearly presented and well written, and the metabolomics data generated here will surely be useful to the broader field of cancer metabolism. I particularly appreciate the authors' efforts to develop an appropriate data normalization strategy (which is a big issue when working with such large metabolomics data sets), and the follow-up experiments (13C-labeling, lipidomics, EMT validation). Therefore, I believe this manuscript will be of interest for the readership of MSB. However, there are currently several issues that should be clarified in a revised manuscript.

Main comments:
1. Relationship to previous literature: The authors' claim that "no systematic characterization of metabolic-wide dysregulation has been accessed to date" (lines 76-77) is unclear to me. As the authors point out themselves, this is not the first attempt to comprehensively access the metabolic state of diverse cancer cell lines. For example, recent works have covered a larger cell panel, but with fewer metabolites measured (PMID 31068703)[1], or a smaller cell panel (~60 cell lines), but with a similar number of measured metabolites (PMID 31015463)[2]. Currently the "added value" this study provides to the field remains somewhat unclear, since the authors make little effort to put their findings in the broader context of the recent literature (the same criticism applies to both back-to-back manuscripts). For example, what made the key finding of this study (i.e. two main metabolic types that are strongly associated with EMT) possible: was is simply the scale of the present study, or the focus on pathways rather than individual metabolites? Would this association have emerged already in the previous studies if they had used the same method to determine metabolic pathway activity? I believe that this manuscript would benefit from an additional paragraph e.g. in the discussion tackling this point.

(1) Different approach

We thank the reviewer for his/her relevant comment. Indeed, this is not the first metabolomic dataset in cell lines. Previous work focused on how individual metabolites are associated with genes/transcripts/proteins (PMID 31068703 & PMID 31015463)[1,2]. We asked how the metabolic network operates across a large panel of cancer cell lines, which 'metabolic routes' are changed and what could be the driver of this rewiring. Thus, our approach differs in the use of metabolomics data (integrated into pathway activity scores encoded by first PCs) and by

confirming flux differences by isotope tracing. As requested by Reviewer 3 (Question 2), we have compared our pathway-centric analysis with the metabolite-centric analysis. The differences are striking:  more traits are associated with the metabolic score than with single metabolite ions (see the answer for details). This result was expected because of the baseline metabolome differences that occur across CCLs. Ultimately, it is one of the reasons that prompted us to adopt pathway scores.

(2) Coverage

We have applied our pathway approach to the data set by Li et al. (PMID 31068703)[1]. After manual curation, we identified 136 (out of the 225) metabolites in KEGG, which resulted in the inference of pathway scores for 19 KEGG pathways. Even though the pathway number is somewhat limited, we could confirm our observation of two distinct types, as seen in Figure A. Both our study and Li et al reveal a dichotomy in cell lines in metabolic pathway activity. This confirms the robustness of your results.

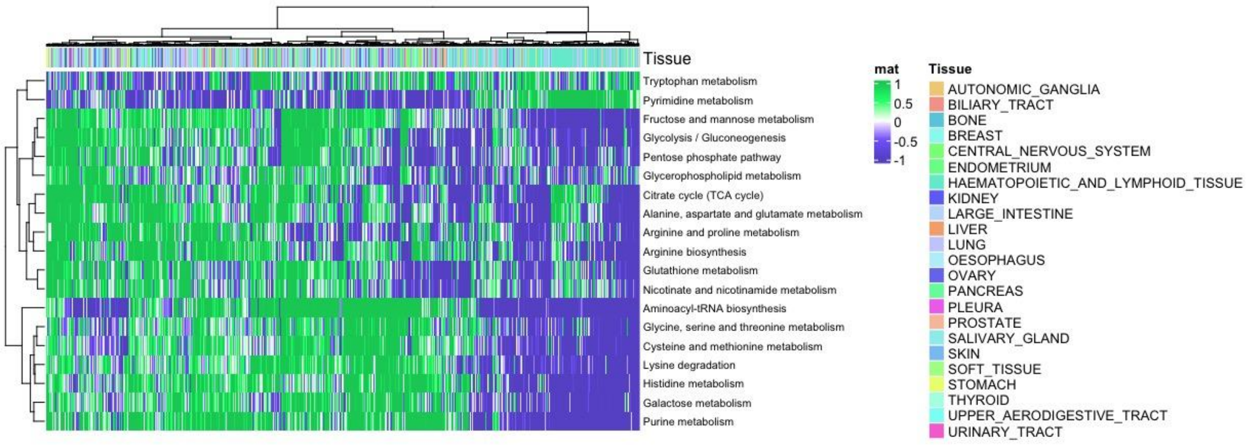


**Figure A. The metabolic activity landscape of Li et al**. Inference of pathway score for 19 KEGG metabolic pathways. Hierarchical clustering of: (above) cell lines, colored by their tissue of origin.

To clarify the aforementioned points, we have added further explication in the discussion (line 340).

2. Lacking detail on EMT validation: I appreciate the authors' efforts to validate the EMT status of selected cell lines with imaging, but currently the description of the methods used is very sparse. For example, I assume the cells were fixed prior to staining, but the method sections (starting in line 544) does not mention anything along these lines. Also, what exactly was the blocking solution the authors used? Please include the necessary information to enable other researchers to reproduce the results.

We thank the reviewer for his comment. We apologize for the omission. We have added further information in the method section (line 559).

3. "Empirical" pathway score: I was very intrigued by the authors' approach to quantify a "pathway activity score" based on principal component analysis. Given that this score is central for most downstream analyses performed in this study, I wonder whether the authors have performed any additional sanity checks (other than the analysis of a previously published yeast data set). For example, how does this metric compare to alternative approaches to quantify metabolic pathway activity, e.g. fraction of significantly altered metabolites?

We appreciate the reviewer's feedback on the pathway score. As we are comparing non-predefined groups, we had to develop a method which would find interesting metabolic changes within unlabelled data. We have tested our lab's previous approach based on affinity propagation clustering (PMID: 28038952) and have compared it to other unsupervised methods (PCA, ICA, NMF, k-means clustering etc.). The fraction of altered metabolites (as suggested by the Reviewer, and implemented in e.g. MetaboAnalyst) was not considered because it relies on thresholds that heavily depend on the number of detectable metabolite ions and neglects the sign of changes even though, biochemically, one would expect coherent changes.

We ended up selecting PCA as (i) the use of principal components is consistent with the expected “distributed” and coherent changes in metabolite levels that one would expect based on biochemistry [this is clearly not the case for ICA], and (ii) it gave quantitative results which correlated to flux in the exemplary yeast data. Some of this preliminary work has been published in a Doctoral thesis (https://doi.org/10.3929/ethz-b-000499744). We omitted all preparative and comparative work from this submission because it would have further diluted an already bulky manuscript, and also because we provide plenty of experimental validation (i.e. with tracing) to support the results provided by the pathway scoring.

Interestingly, an analogous pathway score approach has in the meantime been proposed for the analysis of transcriptomics data by the Ebbels group (PMID: 31684857)[3]. They compared has thoroughly compared single sample pathway analysis approaches application to metabolomics and have reported in their latest preprint that this PCA approach (using PC1) outperforms other methods in precision[4].

We included additional explanations and an explicit reference to the work by the Ebbels group to the discussion (line 339).

4. Interpretation of key finding: A key finding of this paper is that the tested cancer cell lines fall into two broad metabolic groups, which are characterized with generally higher/lower pathway activity and 13C-glucose labeling (type 2 and type 1, respectively), and which are associated with an epithelial or mesenchymal cell state, respectively. This is a striking observation, and I am wondering what the underlying process may be. In my understanding, the generally higher pathway activities and especially the higher 13C incorporation suggest a difference in metabolic turn over. Do these types differ in their growth rate, or in their rate of e.g. protein turn over? Do

these metabolic patterns match what we know about EMT outside of cancer, e.g. in development? I believe that this manuscript may benefit from an additional paragraph discussing these points.

This is a great question that we try to address when designing our follow-up experiments, i.e. for lipidomics and $^{13}C$-tracing. Both for type 1 and type 2, we selected cell lines with different growth rates (line 153, Table EV3 and data table in data availability). As the observed differences between the two types are significant despite mixing of growth rate, we tend to exclude that the observed differences are caused by a baseline difference in growth or metabolic activity. Moreover, we added growth rate data taken from DepMap to our tree enrichment analysis and found only a slight non-significative trend (q-value = 0.69), thus revealing that growth rate is not the biggest driver of these types.

We want to point out that the higher $^{13}C$ incorporation does not occur in all pathways, where some pathways have higher incorporation in type 1, e.g. pentose phosphate pathway.
Interestingly, it was been reported that cells undergoing EMT are often associated with metabolic changes, as the change in phenotype often correlates with a different balance of proliferation versus energy-intensive migration (PMID: 33859341)[5]. We have expanded our discussion to highlight these points.

5. Along the same lines, I am wondering about the causality here: does EMT state determine the metabolic state, or the other way around? The authors already talk about this point in their discussion, but I am wondering whether there is an (experimental) way to test the different possibilities, e.g. using drugs that modulate EMT: would shifting a cell line pharmacological from a epithelial to mesenchymal state also alter its metabolic profile in the expected direction? I understand that testing such conjectures experimentally is outside the scope of this manuscript, but I encourage the authors to at least discuss potential validation experiments in a revised manuscript.

We appreciate the reviewer's feedback on that end. Indeed, discerning the cause and effect of EMT and the observe metabolic changes would be of great value. We believe that future work should address these questions by modulating EMT and measuring the metabolic pathways we have identified in this study. Following the reviewer's guidance, we have added further information on that subject (line 356) and have referred to an extensive review that exactly covers that topic (PMID: 33859341)[5].

6. Lacking "harmonization" of the metabolomics data sets in the two back-to-back manuscripts: It is my understanding that this manuscript is part of a back-to-back submission with another manuscript (MSB-2022-11006), which focuses on elucidating the metabolic differences between these cancer cell lines using the metabolomics data set generated here. However, it is unclear to me why these two manuscripts seem to use quite different data normalization methods, in which not only the number of ions considered, but also the number of cell lines are different. As I mentioned in my report of the other manuscript, I strongly suggest that the authors (of both manuscripts) either harmonize their data normalization pipelines or explain in detail why

different ones are being used. Otherwise, I worry that having two conflicting data sets based on the same original data published back-to-back will lead to substantial confusion in the field.

The differences are due to historical reasons. We'd like to stress that the small differences in dimensions are cosmetic. There are differences in the normalization but they are not conflicting. We have described in detail the normalization procedure in our manuscript. Specifically, we introduced a set of six quantitative criteria that allows us to test a large number of normalization procedures in a possibly agnostic way and using domain knowledge (Supp Table 1, details in Methods section). In other words, the data set that we present (available at https://doi.org/10.3929/ethz-b-000511784) was curated in more detail to ensure that it can be used "safely" for multiple purposes.

Additional comments:
1. Lines 93-94: How did the authors select this panel of cancer cell lines? Was there any specific motivation to select half of them from the same tissue (lung)?

The panel was selected after long meetings and discussions. We wanted to include ca. 200 cell lines. Inclusion of the NCI60 would have guaranteed to cover different tissues of origins and comparability with previous studies. In complement to that, we also wanted to include a large set of cell lines related to the same tissue, and we opted for the lung. Several additional cell lines were added because they were used in chemical screens, or additional data of all kinds were available in-house for analyses of a different kind (not shown here).

2. Lines 240-241: Please add a pointer to the respective 13C-glutamine labeling data in the text (I think its Figure S6).

Thank you for the comment. We added the pointer to the figure.

Reviewer #2:

The paper from Cherkaoui et al. presents a potentially incredibly useful data resource. They generated untargeted metabolomic profiling data entailing 1809 ions for 180 cancer cell lines. Interestingly, the authors identified only two distinct metabolic types. They performed subsequent 13C-flux analysis, lipidomics, and association with gene essentiality data to explore the differences between the two metabolic subsets.

Major points:
1. While there is only one metabolite name in the "Top.annotation.name", multiple KEGG and HMDB IDs are provided in the Top.annotation.ids". Please describe how was the "top" chosen. Among the isobaric metabolites, which was actually used for the pathway score calculation? If the current analysis includes multiple isobaric metabolites per ion for pathway analysis, wouldn't the pathway score be inflated as an artifact? Can the authors show how would the results be affected if only one most abundant representative metabolite is picked for the ion?

We thank the reviewer for his comment. The point is well taken. Pathway analyses are not done on the basis of putative metabolite IDs (i.e. glucose-6P, fructose-6P, etc) or a supposed ranking. Hence, we neither use a “top” candidate nor inflate the counting by replicating the same observation multiple times. Instead, for each pathway, we first convolute all associated metabolites by m/z (tolerance = 0.001 Da), and use each unique ion only once in the pathway analysis. Thereby, we shrink the size of pathways to precisely account for the data we acquired by FIA. We apologize for the confusion caused and we have now updated the method section to clarify the point (line 531).

2. In Figure 5C, the jittered points seem to form subclusters. It seems multiple replicates of the same cell lines were included in the analysis. The author should use a mixed-effect model rather than t-test because the inter-replicate variation is much smaller than the inter-cell line variation. Preferably, different colors should be used to color for different cell lines so replicates from the same cell lines will be in the same color.

Type 1 and Type 2 include non-overlapping (unpaired) sets of cell lines. Hence, it is not possible to formulate the problem as LMM and estimate the variance of the intercept associated with the CL labels. Regardless of the test used on “canonical” lipidomics, we’d like to stress that the main evidence for differences in de novo lipogenesis is provided by the 13C-labeling experiment (Figure 5D). The differences between the two types are striking and coherent across lipid classes.

3. I would like to see a comparison (distribution of metabolite-specific pairwise correlations across shared cell lines for all the shared metabolites) with the existing CCLE metabolomics data to see the cross-study reproducibility of cell line metabolomics data.

We thank the reviewer for his suggestion. We have performed the analysis to assess the similarity between the CCLE dataset (Li et al. PMID 31068703)[1] and our dataset. We found a higher Pearson correlation between the same cell line than between pairs of cell lines chosen at random (Figure B). Of note, CCLE used different media for each cell line which could explain the disparity of some correlation.

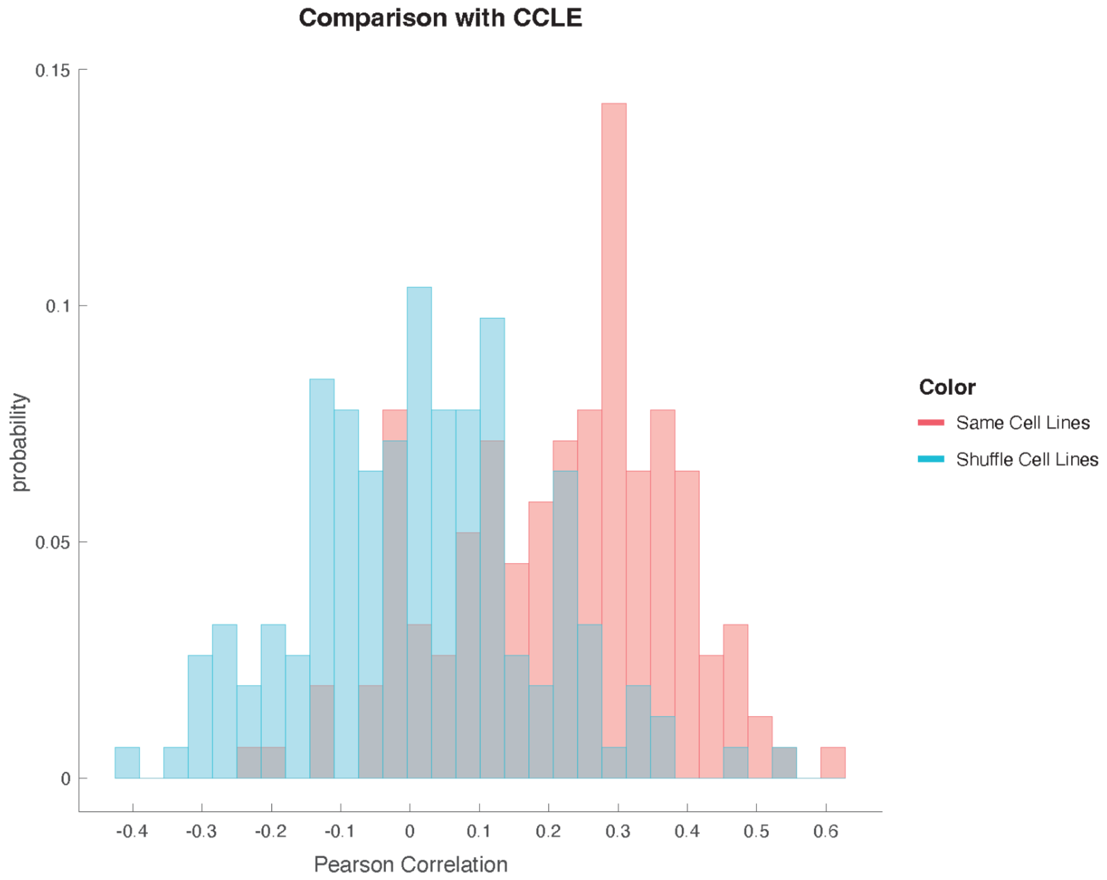


**Figure B. Comparison of our dataset with CCLE (Li et al)**. Person correlation of metabolite levels between the same cell lines and cell line selected at random.

4. NCIH460 has previously been characterized as a mesenchymal cell line (PMID 23091115). Here the authors have assigned it to epithelial despite finding plenty of vimentin expression in Figure S2. In Figure 5D, they compared a breast cancer cell line HS578T to NCIH460 although they mentioned they have selected a pair of breast cancer cell line (T47D being the mesenchymal counterpart) in order to compare by lineage. Please show T47D instead of NCIH460. Can the authors use an EMT geneset to show that cell lines from the two types actually separate by their gene expression on a heatmap?

We appreciate the reviewer's feedback and would like to provide our perspective. EMT is a progressive, non-dichotomic process. Our analysis revealed that type 1 is more advanced in the EMT than type 2 but does not imply that type 2 is purely epithelial.

As requested by the reviewer, we have exchanged NCIH460 with T47D in Figure 5D. As suggested by the reviewer, we report the gene expression below (Figure C). We found that a high number of EMT genes (57 out of 197) are significantly changing between the types, with notably vimentin (VIM) more expressed in type 1 and cadherin in type 2.

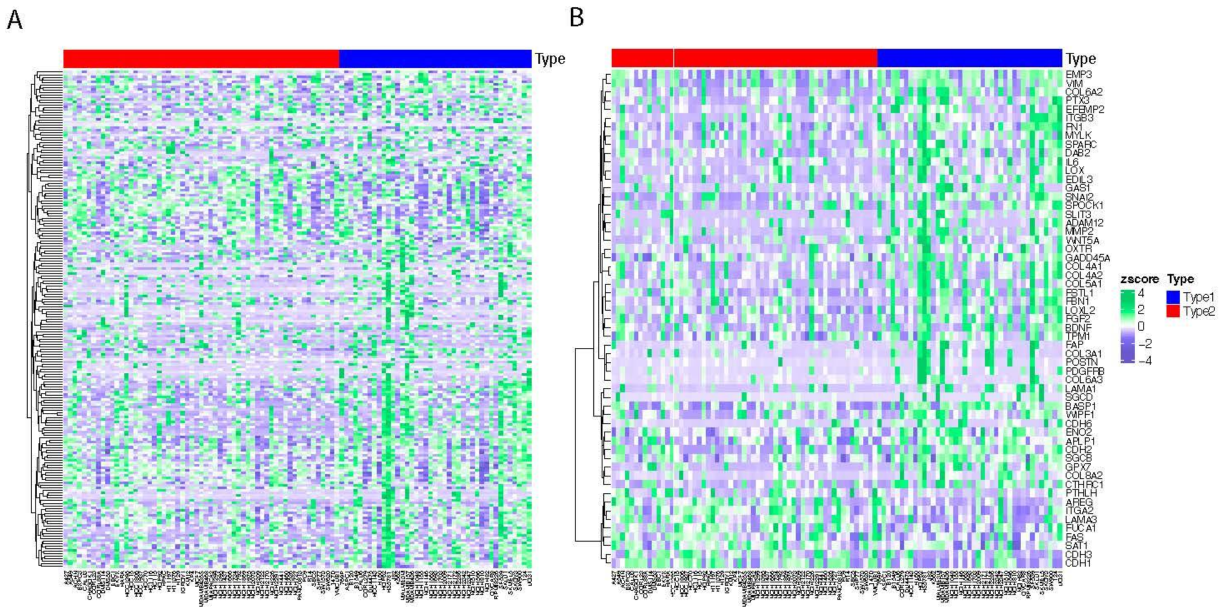


**Figure C. EMT gene expression signature across types**. A. Gene expression EMT in the two metabolic types, where gene set was taken from MSigDB (GSEA). B. Expression of significatively changing genes between type 1 and 2.

Minor points:

1. Legends like the one in Fig 3 can be quite confusing. I suggest the authors break it down into two sublegends, one in grayscale and use different shades to represent different magnitudes of -log10(p-value), the other using color to represent direction.

Thank you for the suggestion. We generated the grayscale version but felt that keeping the colors was more intuitive. This way, any color in the heatmap can be immediately linked to a q-value, and the direction. We hope this is acceptable to the reviewer. The previous color scale reported negative values. This was corrected.

2. Try to derive the combined functional loss status for AXIN1, CDKN2b by integrating mutation, copy number, and RPPA data to see if stronger associations can be found.

We thank the reviewer for his comment. We indeed have tested all omics simultaneously and have applied a combined p-value correction by merging all tested traits/omics, resulting in highly stringent statistical significance level. For AXIN1, the genetic alteration might not be reflected at the gene expression or protein level, which could explain the lack of association. For the tumor suppressor gene CDKN2b, we have added the new proteomics dataset by Nusinow et al.[6] (described in answer to Reviewer 3, Question 6) which includes this protein. Unfortunately, the data are not conclusive as protein measurements were available only for 20 cell lines present in our study. One positive association across omics layers was for THBS1 methylation and gene expression levels, which pointed in the same direction.

3. Please provide Cellosaurus RRID for the cell lines in supplementary data

We added a table with the cell line name and their corresponding Cellosaurus RRID both to the data and the code repositories.

4. Please deposit reproducible codes used for the analyses.

The code is available at https://github.com/zamboni-lab/CCL180-analysis

5. Please provide processed 13C labeling metabolomics and lipidomics data in a separate folder so users don't need to download the whole large zip files that contain the raw data in order to access the processed data.

Done.

6. It may not be intuitive for a general reader to know how fractional contribution is derived, please provide a formula or citation. It seems from their processed data, for the majority of the metabolites, the full set of isotopologues (m+0, m+1, ..., m+n) is not available. What is the reason for such missing data, undetectable signal? Do the authors assume these missing isotopologues as zero values when they performed the analyses shown in Fig 4?

We thank the reviewer for is comment. We added a reference for the fractional contribution at line 583.

Missing values: the intuition of the Reviewer is correct. Missing values indicate isotopologues for which no peak was found in the MS data despite data recursion. As FT-based instruments (like the Orbitrap we used) trim the baseline, missing values are quite common and interpreted as zeros.

7. Figures 3 and 4 are high-level summary figures, can the authors provide some concrete examples (like 5D, but for all cell lines) as supplementary figures?

We have added 4 examples in Appendix Figure S2.

8. Did the authors aggregate their replicate data to cell line-level data (for example, by taking the mean or median) for their analyses? If so, please provide this processed data in the repo too.

Only pathway scores (described at line 529ff) have been averaged. In all further analyses, we did not aggregate by cell lines. All 6 replicates per cell line are reported in the supplementary data files for metabolomics, lipidomics, and labelling experiments.

Reviewer #3:

Cherkaoui et al. present one of the largest metabolic characterization of cancer cell lines to-date, quantifying using untargeted metabolomics over 1,800 metabolic measurements (deprotonated metabolites) across 180 cancer cell lines. This rich resource is used to estimate the activity of 49 metabolic pathways and assess their heterogeneity across cancers. Surprisingly, the authors conclude that cancer cell lines group only into two major clusters, which they relate to other publicly available multi-omics datasets for biomarker association analysis, and experimentally assess / validate their metabolic profiles using 13-C metabolomics, revealing an association with the epithelial to mesenchymal transition (EMT).

In general, the manuscript is very well written and results are well presented, together with the metabolomics resource I am confident this will be of great interest to the broad scientific community with immediate applications to cancer translation (e.g. biomarker discovery). It also expands similar studies - Li et al. (2019) quantified 225 metabolites across 928 cell lines - particularly on the number of metabolic readouts. The main conclusions of the manuscript are interesting and in line with recent cancer cell line multi-omics studies. Nonetheless, further clarifications are needed on the technological and methodological aspects (e.g. metabolic pathway activity score, lack of genomic associations, cancer cell lines diverse growth rates) to understand if these might not impact the conclusions. The sole focus of the downstream analyses on the metabolic pathway activity scores might have limited the exploration of the richness of this data, as only a portion (20%) of the data is used to calculate these scores

Major points:

1. Can the authors comment on the considerable reduction of the metabolic measurements when only considering those that map to KEGG pathways? If I understand correctly, only 367 annotated ions could be mapped (~20% of all quantified metabolic readouts). Also, the pathway activity scores are mainly derived from PC1, would this mean that metabolic pathway activities will be mostly related to the main driver of variability in the metabolomics and miss other likely interesting sources of variability? Indeed, it would be expected that the first PCs would be associated with EMT as shown by other orthogonal omics (Li et al. 2017, Rajapakse et al. 2018, Gonçalves et al. 2022).

For the purpose of our study on metabolic heterogeneity across CLs, it was important to cover primary metabolism densely. This is in the range of a few 100s of compounds and, hence, we are very satisfied with having obtained 367 matched metabolite ions and 49 pathways with sufficient coverage. To compare, the previous study by Li et al.[1] had data for only 19 pathways (Reviewer 1, Question 1). All additional compounds beyond primary metabolism are more difficult to embed in a similar analysis because they have more uncertain annotation, tend to be widespread over very heterogeneous classes of compounds or aren’t organized in pathways (e.g. lipids).

We believe that this association of EMT found in other omics studies is extremely encouraging. As pointed out by the reviewer, Gonçalves et al.[7] have found that protein levels' main source of variation is also EMT. With our study, we can now pinpoint which metabolic pathway activity is implicated in both mesenchymal and epithelial cells. To the best of our knowledge, this is the first time such association has been reported in a large-scale metabolomic study.

2. Related to my previous main point, the clustering of the cancer cell lines into two major groups is indeed interesting. Considering this conclusion relies on the pathway activity scores, could the authors comment if the cancer cell lines hierarchical clustering and conclusions are preserved when calculated using the total measured ions (1,809), i.e. if other cancer cell lines sub-clusters start to emerge (as it seems the case from Figure 2 and 3)? Also, can the authors comment on how their findings compare with the cell line grouping achieved from the independent metabolic dataset Li et al. (2019)?

As suggested, we have performed the enrichment on the tree on the measured ions instead of the pathway activity scores. As displayed below in the resulting clustering, where we did observe a similar pattern as for the pathway score analysis (Figure D).

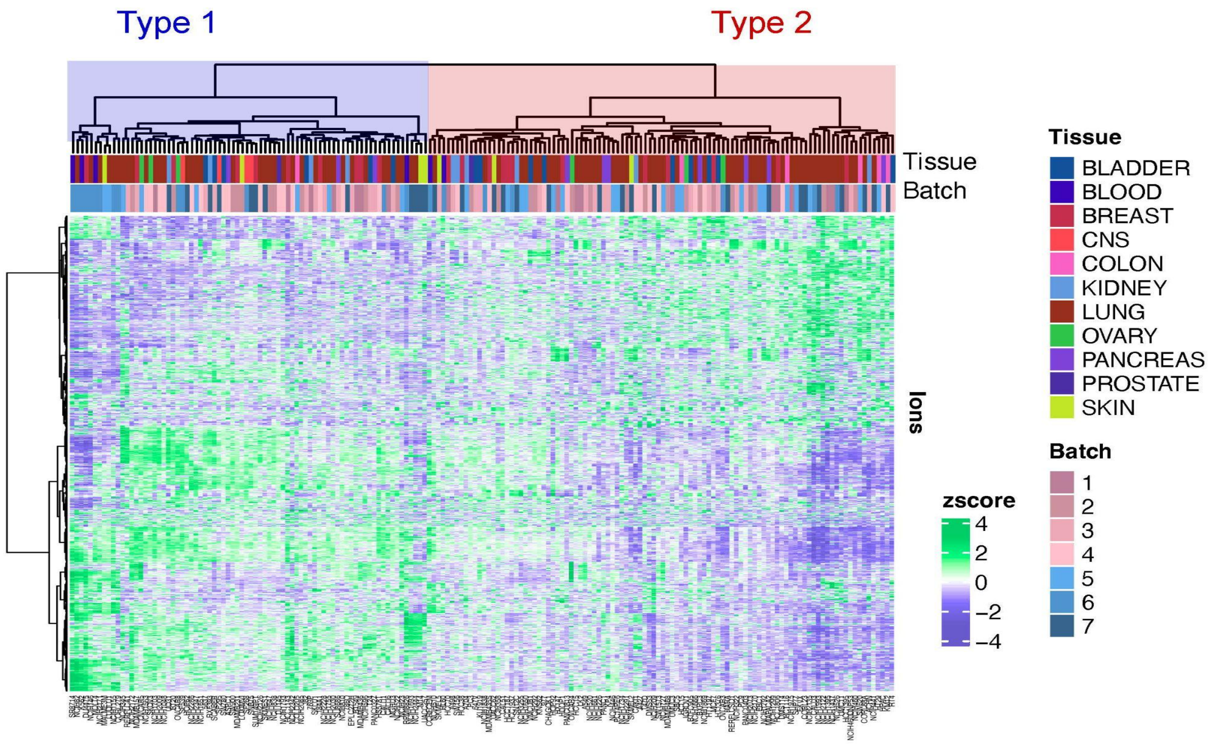


**Figure D. Analysis of clustering by metabolic ions**

To compare the groups obtained from pathway score and ions, we have calculated how many cell lines overlapped in the two main types. We have found a good overlap, where a high number of cell lines clusters into the same types.

| Number of overlapping cell lines | | Pathway Score Clustering | |
|---|---|---|---|
| | | Type 1 | Type 2 |
| Ion Clustering | Type 1 | 74 | 28 |
| | Type 2 | 45 | 33 |

**Table 1. Similarity in the clustering by Pathway Score clustering compared to Ions clustering.**

To address if indeed the conclusions are preserved when calculated using the total measured ions, we have assessed the 'tree enrichment' analysis on the ions. Overall, we have identified considerably fewer associations: 362 instead of 938 using our pathway scores (Table EV1). The main association to EMT/TGFB/HIF1A is preserved, confirming the robustness of our finding and the added value of our pathway score approach.

| Type | Trait | # of Traits | # Significant | Source |
|---|---|---|---|---|
| Experiment | Confluence/Suspension | 2 | 1 | This Study |
| | Batch | 7 | 1 | This Study |
| | Growth Rate | 1 | 0 | CCLE[13] |
| Metadata | Tissue Type | 11 | 1 | This Study |
| | Pathology | 4 | 0 | CCLE[13] |
| | Histology | 23 | 0 | CCLE[13] |
| | Histology Subtype | 69 | 0 | CCLE[13] |
| | Cancer Type | 38 | 0 | CCLE[13] |
| | Ethnicity | 3 | 0 | CCLE[13] |
| | Mutation rate | 1 | 0 | CCLE[13] |
| Omics | Mutation | 705 | 0 | Li et al.[12] |

| | | | | |
|---|---|---|---|---|
| | Copy Number | 61 | 1 | Li et al.[12] |
| | Methylation | 2114 | 134 | Li et al.[12] |
| | Transcript | 56 318 | 106 | CCLE[13] |
| | Protein (RPPA) | 214 | 18 | CCLE[13] |
| | Protein (TMT-MS) | 12 724 | 38 | Nusinow et al. |
| Aggregate | Transcription Factor | 743 | 59 | Ortmayr et al.[31] |
| | Signaling Pathway | 14 | 2 | Schubert et al.[18] |
| | Epithelial to Mesenchymal Transition | 1 | 1 | Rajapakse et al.[38] |
| TOTAL | | 73 053 | 362 | |

**Table 2. Similarity in the clustering by pathway scores compared to ions.**

3. Presumably this resource has overlapping cell lines with the independent metabolic map presented by Li et al. (2019), and therefore it would be important to understand how these datasets compare to assess reproducibility. For example, could the authors correlate the metabolic profiles (e.g. pathway activity scores, metabolic measurements or other metrics) of the same cancer cell line in both datasets and how these compared to the random expectation (i.e. all-vs-all)?

We thank the reviewer for his suggestion. We have performed the analysis to assess the similarity between the CCLE dataset (Li et al. PMID 31068703)[1] and our dataset. We found a higher Pearson correlation between the same cell line than between pairs of cell lines chosen at random (Figure B). Of note, CCLE used different media for each cell line which could explain the disparity of some correlation.

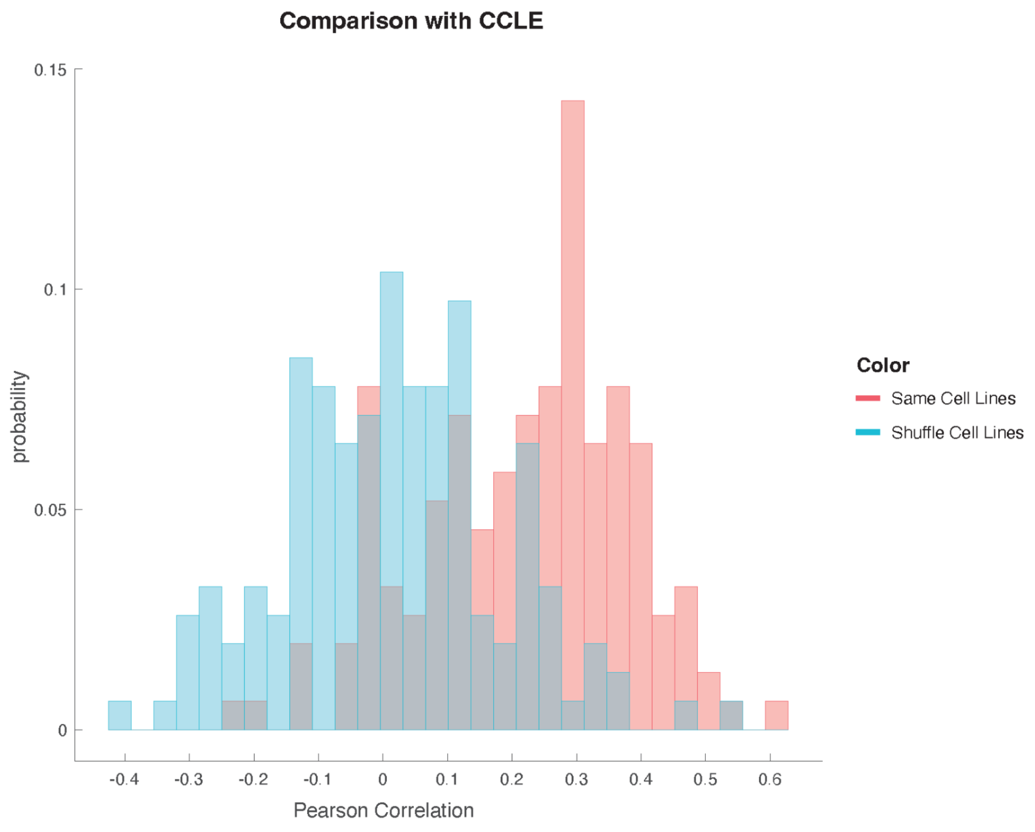


**Figure B. Comparison of our dataset with CCLE (Li et al)**. Person correlation of metabolite levels between the same cell lines and cell line selected at random.

4. Cancer cell lines can be very heterogeneous (e.g. tissue-type, growth rate, cell size, etc) and this becomes an important consideration for large-scale studies, such as this, to avoid potential confounding factors. The authors have already taken an important step towards this by considering similar culture conditions. Have the authors corrected or assessed the potential impact of cell lines' divergent growth rates on the metabolic measurements? Also, could the authors comment on the potential impact of cell size (with regards to its relation with tumor invasion and EMT)?

We have addressed this question above (Reviewer 1, Question 3). Moreover, we want to point out that we did not find a significant association to confluency, which was measured in our study. Factors such as confluence, cell size, and cell number are expected to affect equally intracellular metabolites. In other words, we would expect a coherent increase/decrease of most metabolites proportionally to the starting volume/material. However, such deviations are effectively corrected by the normalization procedure we adopted.

5. The lack of genomic associations is surprising. Could this be a consequence of using the pathway activity scores? Would a systematic association with deprotonated ions expand the

potential for identifying associations? Perhaps pathway level analysis could be carried after the association analysis using the effect sizes?

As pathway scores evaluate functional changes in the metabolic network, this result is not completely unexpected as metabolism is coordinated by much more complicated layers of regulation. For our analysis, we have used curated mutational data from Li et al (PMID 31068703)[1]. A complete analysis between individual mutations and metabolites has been performed by the complementary manuscript by Shorthouse et al. (MSB-2022-11006).

6. For the proteomics analysis the authors focused on reverse-phase protein array (RPPA), whilst this has some very specific advantages (e.g. phospho antibodies) a more systematic protein measurement based on TMT-MS is now available quantifying the proteomes (>10,000 proteins) of 375 cancer cell lines (Nusinow et al. 2020). It would be worth expanding the current analysis with this dataset. For example, this would give insights into other canonical markers of EMT, such as VIM.

We thank the reviewer for this suggestion. We have performed the analysis and have added the significant proteins to our analysis (Appendix Figure S1. 2, Table EV2 and data table in Data availability). We could confirm some of the associations found by RPPA with e-cadherin (CDH1) but not with the p-cadherin (CDH3) and vimentin (VIM). Interestingly, gene expression profiles of these genes (Figure E) and our microscopy results pointed toward strong differences between the types.

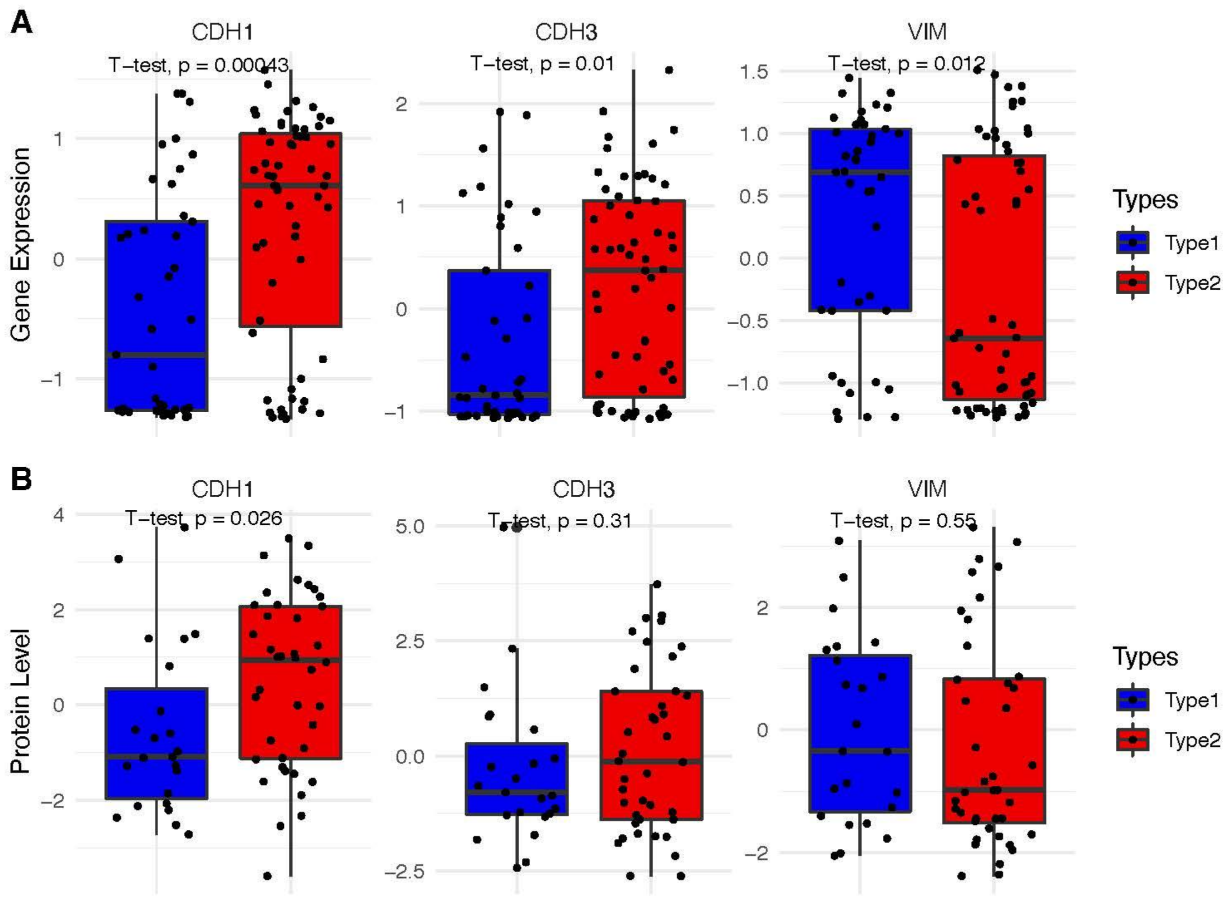


**Figure E. Comparison of genes expression and protein levels of mentioned EMT genes. A. Gene expression taken from CCLE. B. Protein levels taken from Nusinow et al.[6].**

Minor points:

1. From a PCA analysis of both the pathway activity and metabolite ions what is the percentage of variance captured across the top PCs? Are multiple PCs associated with EMT?

On average, PC1 captures most of the explained variance, on average 36.6%. EMT correlated mostly with PC1 compared to PC2.

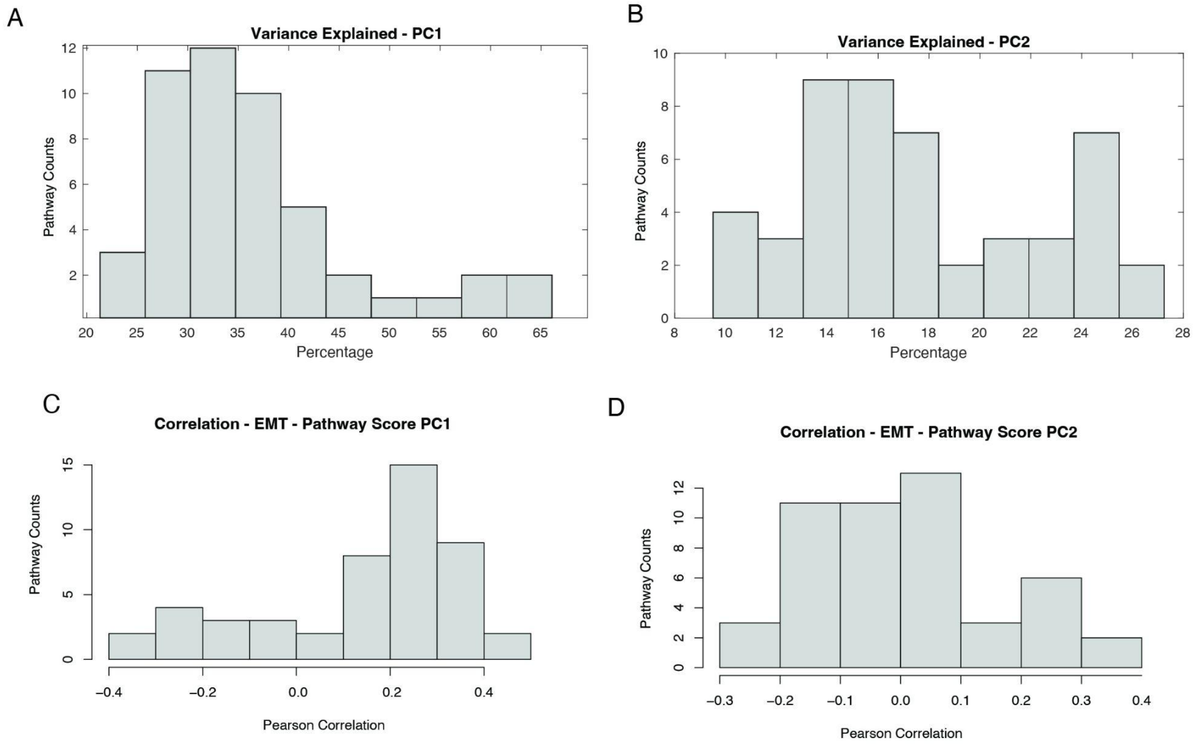


**Figure F. First PCA component analysis. A. Distribution of variance explained in PC1 of all pathways B. Distribution of variance explained in PC2 of all pathways. C. Correlation of EMT to PC1 of all pathways. D. Correlation of EMT to PC2 of all pathways.**

2. Figure 6B IDH2 and SDHAF4 CRISPR-Cas9 gene essentiality scores are overall very low, whilst the mean difference is significant these genes are likely non-essential across all cell lines. SDHAF4 does show some dependency in a small number of cancer cell lines, from the DepMap portal these are enriched for melanoma cancer cell lines.

We agree with the reviewer that IDH2 and SDHAF4 are not as essential as, for example, glycolytic genes. However, the goal of this analysis was to confirm the relative differences between types, where one would expect that reactions which are more metabolically active, carrying more flux, in two different conditions, will be more sensitive to inhibition.

## References


1. Li, H. *et al.* The landscape of cancer cell line metabolism. *Nature Medicine* **25**, 850–860 (2019).
2. Ortmayr, K., Dubuis, S. & Zampieri, M. Metabolic profiling of cancer cells reveals genome-wide crosstalk between transcriptional regulators and metabolism. *Nature Communications* **10**, (2019).
3. Segura-Lepe, M. P., Keun, H. C. & Ebbels, T. M. D. Predictive modelling using pathway scores: robustness and significance of pathway collections. *BMC Bioinformatics* **20**, 543 (2019).
4. Wieder, C., Lai, R. P. & Ebbels, T. Single sample pathway analysis in metabolomics: performance evaluation and application. *bioRxiv* 2022.04.11.487976 (2022) doi:10.1101/2022.04.11.487976.
5. Jia, D. *et al.* Towards decoding the coupled decision-making of metabolism and epithelial-to-mesenchymal transition in cancer. *British Journal of Cancer 2021 124:12* **124**, 1902–1911 (2021).
6. Nusinow, D. P. *et al.* Quantitative Proteomics of the Cancer Cell Line Encyclopedia. *Cell* **180**, 387-402.e16 (2020).
7. Gonçalves, E. *et al.* Pan-cancer proteomic map of 949 human cell lines reveals principles of cancer vulnerabilities. *bioRxiv* 2022.02.26.482008 (2022) doi:10.1101/2022.02.26.482008.

Thank you for sending us your revised manuscript. We have now heard back from the three reviewers who were asked to evaluate your study. You will see from the comments below that while Reviewers #1 and #3 are overall satisfied with the revisions, Reviewer #2 still raised several concerns, which need to be addressed in a revision of the current manuscript.

Without reiterating all the points, some of the more substantial issues are the following:

- Reviewer #2 is concerned about the reported association between metabolic types and EMT types. During our cross-commenting process (in which the reviewers are given a chance to make additional comments, including on each other's reports), Reviewer #1 mentioned that they think "the authors provide sufficient evidence that their metabolic types are associated with EMT status". Therefore, we would ask you to follow Reviewer #1' suggestions to better clarify this point.

- Reviewer #2 raised significant concerns about Figure 5. Considering Reviewers #1 and #2's comments, please provide results for all cell line pairs in Appendix.

- Reviewer #2's comment regarding LMM analysis needs to be carefully addressed.

--------------------------------------------------------

Reviewer #1:

I thank the authors for addressing all my concerns, and I am happy to recommend this manuscript for publication.

I only have a few minor suggestions:

1. Maybe you can also define what you mean by the term "metabolic reprogramming" (or "metabolic-wide reprogramming") in the context of this study (Lines 79-80). In my naïve understanding, the term "reprogramming" implies a comparison with a reference state (e.g. when comparing metabolomics data between two conditions), but I don't think this is how you use the term here (rather, I assume you essentially mean "metabolic-wide pathway activity").

2. Please add the reference 5 from the response-to-reviewers (PMID 33859341) to the main text (Line 363).

3. In their response to reviewer 3 (point 1), the authors note that recent proteomics data also suggests a dominant role of EMT in explaining proteome variability across cell lines (Goncalves et al 2022). This is actually a very interesting point: maybe the authors can include a sentence along these lines in their discussion?

Reviewer #2:

The point-by-point response is very helpful and have addressed many of my questions. This is a paper with a lot of good results. However, I still have the following issues/comments that I hope the authors can address.

The github repo is very helpful! But I can't find the link in the merged manuscript pdf, please also put the link in the paper.

In Table EV1, what does "FC" and "AA" stand for in "Batch Scoring FC", "FC Reproducibility,""FC Reproducibility AA"? Please provide full name and explanation for these and other headers with abbreviations in the legend sheet.

In Figure C, in the heatmap on the left, I can't see much gene expression difference between type 1 and type 2 cell lines for the EMT geneset. The Gonçalves paper found a very strong association between proteomic variations and vimentin and e-cadherin levels. In this metabolomics paper, EMT marker assessment were only made for selected cell lines from the whole panel. The authors should directly compare VIM and CDH1 RNA and/or protein levels in all type1 and type2 cells.

I do not understand the author's point about "Type 1 and Type 2 include non-overlapping (unpaired) sets of cell lines. Hence, it is not possible", LMM doesn't have to use paired samples. In R, with the lme4 package, the author can use fit = lmer(metabolite ~ type + (1|cell_line), data=data) to control for by-cell-line variability.

I agree difference in 5D is striking, but with the author's code, I checked the other lines and found HS578T not very representative of type 1. It seems to me that HS578T and H460 are skewed towards low and high labeling respectively whereas the rest fo the cell lines all have medium level labeling. The other cancer type-matched cell lines OVCAR5 and OVCAR3 barely showed any difference. I hope the authors can provide the full panel (for the 9 cell lines) in a supplementary figure so readers can assess these results more objectively. Overall, I believe there is metabolic heterogeneity in de novo lipid synthesis among this panel of cell lines, but I do not think the association between this particular metabolic phenotype and EMT types is well supported.

"Only pathway scores (described at line 529ff) have been averaged. In all further analyses, we did not aggregate by cell lines." While it is very helpful to report data in replicates, to facilitate reuse of the data, please generate cell line-level data to be included in the supplementary data file. This will facilitate analysis with existing molecular data generated by other studies.

Reviewer #3:

The authors have addressed all my comments successfully, adding further details to the analyses and comparisons with other datasets strengthened the manuscript and its conclusions. I have no doubt this will be an important study and resource for the community.

-------------------------

Referee Cross-Commenting

Reviewer #1

Regarding the comments made by reviewer 2:

1. I do think the authors provide sufficient evidence that their metabolic types are associated with EMT status. For example, in figure C of their rebuttal, the authors do identify about 50 EMT-related genes whose expression is significantly different between type 1 and type 2 cell lines. In order to clarify this point, I would suggest that a) the authors include this figure in the manuscript (e.g. as a supplementary figure), b) explicitly point out in the text that a recent proteomics based study has come to a similar conclusion (Goncalves et al), and c) include the caveat that they only tested EMT markers for a few selected cell lines (in my eyes, it is completely fine to label the association of metabolic types with EMT status as a "hypothesis supported by orthogonal data", instead of a "definite proof").

2. Regarding the differences in lipid labeling patterns between metabolic types: I did not have the opportunity to check all of the cell line pairs (as reviewer 2 did). But this reviewers' suggestion to show all cell line pairs in the supplementary data seems fair. I was already convinced that the summary plots in figure 5 (i.e. 5A and 5E) support the authors' claims.

Reviewer #2

I still hold my opinion that LMM should be used, and the authors should provide a figure to show de novo lipid synthesis for all their cell lines that were tested.

Reviewer #3

I agree with the reviewers comments, I think these changes will help dissipate some concerns about the EMT clustering.

Thank you for your constructive feedback. We are submitting a revised version of our manuscript. Specific answers are embedded below (in red).

Best regards,

Nicola & co-authors.

Dear Prof Zamboni,

Thank you for sending us your revised manuscript. We have now heard back from the three reviewers who were asked to evaluate your study. You will see from the comments below that while Reviewers #1 and #3 are overall satisfied with the revisions, Reviewer #2 still raised several concerns, which need to be addressed in a revision of the current manuscript.

Without reiterating all the points, some of the more substantial issues are the following:

- Reviewer #2 is concerned about the reported association between metabolic types and EMT types. During our cross-commenting process (in which the reviewers are given a chance to make additional comments, including on each other's reports), Reviewer #1 mentioned that they think "the authors provide sufficient evidence that their metabolic types are associated with EMT status". Therefore, we would ask you to follow Reviewer #1' suggestions to better clarify this point.

A: We thank all Reviewers and the Editor for their helpful suggestions. In this case, we adhered to Reviewer #1. Details are reported below.

- Reviewer #2 raised significant concerns about Figure 5. Considering Reviewers #1 and #2's comments, please provide results for all cell line pairs in Appendix.

A: We added Figure EV4 with all cell lines for the case of TG50:1 (as in Figure 5D).

- Reviewer #2's comment regarding LMM analysis needs to be carefully addressed.

A: To demonstrate the significance of the observed changes, we switched to a non-parametric test that doesn’t depend on assumptions on the distribution (as a t-test). For reasons outlined in detail below, LMM isn’t suited for this problem.

On a more editorial level, please do the following:

.

**Reviewer #1:**

I thank the authors for addressing all my concerns, and I am happy to recommend this manuscript for publication.

I only have a few minor suggestions:

1. Maybe you can also define what you mean by the term "metabolic reprogramming" (or "metabolic-wide reprogramming") in the context of this study (Lines 79-80). In my naïve understanding, the term "reprogramming" implies a comparison with a reference state (e.g. when comparing metabolomics data between two conditions), but I don't think this is how you use the term here (rather, I assume you essentially mean "metabolic-wide pathway activity").

A: We thank the reviewer and adapted the text accordingly.

2. Please add the reference 5 from the response-to-reviewers (PMID 33859341) to the main text (Line 363).

A: Added.

3. In their response to reviewer 3 (point 1), the authors note that recent proteomics data also suggests a dominant role of EMT in explaining proteome variability across cell lines (Goncalves et al 2022). This is actually a very interesting point: maybe the authors can include a sentence along these lines in their discussion?

A: Added (line 380).

**Reviewer #2:**

The point-by-point response is very helpful and have addressed many of my questions. This is a paper with a lot of good results. However, I still have the following issues/comments that I hope the authors can address.

The github repo is very helpful! But I can't find the link in the merged manuscript pdf, please also put the link in the paper.

A: We added the link to Data availability.

In Table EV1, what does "FC" and "AA" stand for in "Batch Scoring FC", "FC Reproducibility,""FC Reproducibility AA"? Please provide full name and explanation for these and other headers with abbreviations in the legend sheet.

A: The terms are explained in detail in the Method section (Data normalization, line 445-490). This information is provided on line 6 of the legend sheet.

In Figure C, in the heatmap on the left, I can't see much gene expression difference between type 1 and type 2 cell lines for the EMT geneset. The Gonçalves paper found a very strong association between proteomic variations and vimentin and e-cadherin levels. In this metabolomics paper, EMT marker assessment were only made for selected cell lines from the whole panel. The authors should directly compare VIM and CDH1 RNA and/or protein levels in all type1 and type2 cells.

A: We thank the Reviewer for his/her comment. In Figure C all EMT genes were added in all overlapping cell lines. For the expression of VIM and CDH1, we addressed this point in the previous revision with ed the comparisons in Figure E (reviewer 3, question 6). Expression of both VIM and CDH1 were significantly different, and the protein level of CDH1 was significant when comparing Type 1 and 2 across all overlapping cell lines.

I do not understand the author's point about "Type 1 and Type 2 include non-overlapping (unpaired) sets of cell lines. Hence, it is not possible", LMM doesn't have to use paired samples. In R, with the lme4 package, the author can use fit = lmer(metabolite ~ type + (1|cell_line), data=data) to control for by-cell-line variability.

A: We apologize for the choice of wording. The term “not possible” was wrong, but we stand to the point that an LMM regression isn’t suited to assess whether the 13C enrichment is significantly different between the two types. If we fit a FC ~ Type + (1 | CL) model as

suggested, the intercept is estimated as a fixed value for each cell line. Since we don't have observations in which CLs and Types are mixed, shifts in enrichments between any cell line are masked by the estimated intercept. Not surprisingly, if we apply LMM to the 13C-enrichments reported in Figure 5E, all differences among cell lines are significant, but none of the differences between types is. This is show exemplarity for the TG 50:1 case that we use in Figure 5D. Similar results are obtained for all of the other lipids. Note that p-values are estimated with the afex package.

```
Linear mixed model fit by REML. t-tests use Satterthwaite's method
['lmerModLmerTest']
Formula: frac ~ Typing + (1 | CellLines)
   Data: FC

REML criterion at convergence: -270.4

Scaled residuals:
    Min      1Q  Median      3Q     Max
-1.4957 -0.4948 -0.0545  0.2988  3.3713

Random effects:
 Groups    Name        Variance  Std.Dev.
 CellLines (Intercept) 0.0086554 0.09303
 Residual              0.0001107 0.01052
Number of obs: 53, groups:  CellLines, 9

Fixed effects:
             Estimate Std. Error      df t value Pr(>|t|)
(Intercept)   0.36883    0.04657 6.99996   7.920 9.72e-05 ***
TypingType 2  0.15360    0.06248 7.00049   2.458   0.0436 *
---
Signif. codes:  0 '***' 0.001 '**' 0.01 '*' 0.05 '.' 0.1 ' ' 1

Correlation of Fixed Effects:
            (Intr)
TypingType2 -0.745
```

Therefore, we have to approach the problem differently. The original concern of the Reviewer is on the use of t-tests to compare non-normal distributions. Newly, **we addressed this concern by adopting the Wilcoxon-Mann-Whitney rank-sum test, i.e. a non-parametric alternative that is agnostic of assumptions on distributions specific**. The results are reported in Figure 5E: we found that $p < 0.05$ for 11 out 15 lipids analyzed, which supports the claim that 13C enrichment of lipids and thus the relative contribution of de novo biosynthesis is different between the groups.

I agree difference in 5D is striking, but with the author's code, I checked the other lines and found HS578T not very representative of type 1. It seems to me that HS578T and H460 are skewed towards low and high labeling respectively whereas the rest fo the cell lines all have

medium level labeling. The other cancer type-matched cell lines OVCAR5 and OVCAR3 barely showed any difference. I hope the authors can provide the full panel (for the 9 cell lines) in a supplementary figure so readers can assess these results more objectively. Overall, I believe there is metabolic heterogeneity in de novo lipid synthesis among this panel of cell lines, but I do not think the association between this particular metabolic phenotype and EMT types is well supported.

A: The point about OVCAR5 is well taken. The figure was added as suggested (EV4). Overall, the claim is not based on visual inspection of single cell lines, but on the analysis of the total 13C-enrichment of 15 high abundant lipids. This was found to be significant for 11 of 15, and the 4 non-significant lipids show a coherent trend. We have rephrased the text accordingly (lines 293ff).

"Only pathway scores (described at line 529ff) have been averaged. In all further analyses, we did not aggregate by cell lines." While it is very helpful to report data in replicates, to facilitate reuse of the data, please generate cell line-level data to be included in the supplementary data file. This will facilitate analysis with existing molecular data generated by other studies.

A: This is an unusual request, which we are hesitant to fulfil. The information provided by biological replicates is essential to evaluate the significance of any difference. A good example is given just above by the comment of Reviewer #2 on the labelling patterns. We don't want to encourage reusing only the means and would refrain from providing them. Any scientist that wants to use means in a knowledgeably way will manage to calculate them with a couple of lines of code.

**Reviewer #3:**

The authors have addressed all my comments successfully, adding further details to the analyses and comparisons with other datasets strengthened the manuscript and its conclusions. I have no doubt this will be an important study and resource for the community.

A: Many thanks for the support and the constructive help!

-------------------------

Referee Cross-Commenting

Reviewer #1

Regarding the comments made by reviewer 2:

1. I do think the authors provide sufficient evidence that their metabolic types are associated with EMT status. For example, in figure C of their rebuttal, the authors do identify about 50 EMT-related genes whose expression is significantly different between type 1 and type 2 cell lines. In order to clarify this point, I would suggest that a) the authors include this figure in the manuscript (e.g. as a supplementary figure), b) explicitly point out in the text that a recent proteomics based study has come to a similar conclusion (Goncalves et al), and c) include the caveat that they only tested EMT markers for a few selected cell lines (in my eyes, it is completely fine to label the association of metabolic types with EMT status as a "hypothesis supported by orthogonal data", instead of a "definite proof").

A: Thank you for the support. Appendix Figure S2 was added to the manuscript to reflect these EMT expression changes and the text was changed accordingly.

2. Regarding the differences in lipid labeling patterns between metabolic types: I did not have the opportunity to check all of the cell line pairs (as reviewer 2 did). But this reviewers' suggestion to show all cell line pairs in the supplementary data seems fair. I was already convinced that the summary plots in figure 5 (i.e. 5A and 5E) support the authors' claims.

Thank you. We replaced the test in figure 5E with the non-parametric rank sum test, which doesn't assume Gaussianity. The results are similar and support the conclusion that type 2 has an higher 13C enrichment originating from 13C-glucose.

Reviewer #2

I still hold my opinion that LMM should be used, and the authors should provide a figure to show de novo lipid synthesis for all their cell lines that were tested.

A: The limitation of LMM for this question is described above. To bypass the problem of non-normal distributions, we now use the Wilcoxon Mann-Whitney test. We added an EV figure 4 for the case of TG 50:1 as suggested by the Reviewer.

Reviewer #3

I agree with the reviewers comments, I think these changes will help dissipate some concerns about the EMT clustering.

Thank you again for sending us your revised manuscript. We are now satisfied with the modifications made and I am pleased to inform you that your paper has been accepted for publication.

## EMBO Press Author Checklist


| | |
|---|---|
| Corresponding Author Name: Nicola Zamboni | |
| Journal Submitted to: Molecular Systems Biology | |
| Manuscript Number: MSB-2022-11033 | |


**USEFUL LINKS FOR COMPLETING THIS FORM**
The EMBO Journal - Author Guidelines
EMBO Reports - Author Guidelines
Molecular Systems Biology - Author Guidelines
EMBO Molecular Medicine - Author Guidelines

**Reporting Checklist for Life Science Articles (updated January**
This checklist is adapted from Materials Design Analysis Reporting (MDAR) Checklist for Authors. MDAR establishes a minimum set of requirements in transparent reporting in the life sciences (see Statement of Task: 10.31222/osf.io/9sm4x). Please follow the journal's guidelines in preparing your
**Please note that a copy of this checklist will be published alongside your article.**

**Abridged guidelines for figures**
**1. Data**
The data shown in figures should satisfy the following conditions:
- the data were obtained and processed according to the field's best practice and are presented to reflect the results of the experiments in an accurate and unbiased manner.
- ideally, figure panels should include only measurements that are directly comparable to each other and obtained with the same assay.
- plots include clearly labeled error bars for independent experiments and sample sizes. Unless justified, error bars should not be shown for technical
- if n<5, the individual data points from each experiment should be plotted. Any statistical test employed should be justified.
- Source Data should be included to report the data underlying figures according to the guidelines set out in the authorship guidelines on Data

**2. Captions**
Each figure caption should contain the following information, for each panel where they are relevant:
- a specification of the experimental system investigated (eg cell line, species name).
- the assay(s) and method(s) used to carry out the reported observations and measurements.
- an explicit mention of the biological and chemical entity(ies) that are being measured.
- an explicit mention of the biological and chemical entity(ies) that are altered/varied/perturbed in a controlled manner.
- the exact sample size (n) for each experimental group/condition, given as a number, not a range;
- a description of the sample collection allowing the reader to understand whether the samples represent technical or biological replicates (including how many animals, litters, cultures, etc.).
- a statement of how many times the experiment shown was independently replicated in the laboratory.
- definitions of statistical methods and measures:
  - common tests, such as t-test (please specify whether paired vs. unpaired), simple χ2 tests, Wilcoxon and Mann-Whitney tests, can be unambiguously identified by name only, but more complex techniques should be described in the methods section;
  - are tests one-sided or two-sided?
  - are there adjustments for multiple comparisons?
  - exact statistical test results, e.g., P values = x but not P values < x;
  - definition of 'center values' as median or average;
  - definition of error bars as s.d. or s.e.m.

**Please complete ALL of the questions below.**
**Select "Not Applicable" only when the requested information is not relevant for your study.**

**Materials**

| Newly Created Materials | Information included in the manuscript? | In which section is the information available? (Reagents and Tools Table, Materials and Methods, Figures, Data Availability Section) |
|---|---|---|
| New materials and reagents need to be available; do any restrictions apply? | Not Applicable | Not Applicable |

| Antibodies | Information included in the manuscript? | In which section is the information available? (Reagents and Tools Table, Materials and Methods, Figures, Data Availability Section) |
|---|---|---|
| For **antibodies** provide the following information:<br>- Commercial antibodies: RRID (if possible) or supplier name, catalogue number and or/clone number<br>- Non-commercial: RRID or citation | Yes | Methods - Cell imaging and image analysis |

| DNA and RNA sequences | Information included in the manuscript? | In which section is the information available? (Reagents and Tools Table, Materials and Methods, Figures, Data Availability Section) |
|---|---|---|
| **Short novel DNA or RNA including primers, probes**: provide the sequences. | Not Applicable | Not Applicable |

| Cell materials | Information included in the manuscript? | In which section is the information available? (Reagents and Tools Table, Materials and Methods, Figures, Data Availability Section) |
|---|---|---|
| **Cell lines:** Provide species information, strain. Provide accession number in repository **OR** supplier name, catalog number, clone number, and/**OR** RRID. | Yes | Data Availability - RRID in CellLine_CellorusIDs.txt |
| **Primary cultures:** Provide species, strain, sex of origin, genetic modification status. | Not Applicable | Not Applicable |
| Report if the cell lines were recently **authenticated** (e.g., by STR profiling) and tested for mycoplasma contamination. | Yes | Yes - See Methods - Cell Culture |

| Experimental animals | Information included in the manuscript? | In which section is the information available? (Reagents and Tools Table, Materials and Methods, Figures, Data Availability Section) |
|---|---|---|
| **Laboratory animals or Model organisms:** Provide species, strain, sex, age, genetic modification status. Provide accession number in repository **OR** supplier name, catalog number, clone number, **OR** RRID. | Not Applicable | Not Applicable |
| **Animal observed in or captured from the field:** Provide species, sex, and age where possible**.** | Not Applicable | Not Applicable |
| Please detail **housing and husbandry conditions**. | Not Applicable | Not Applicable |

| Plants and microbes | Information included in the manuscript? | In which section is the information available? (Reagents and Tools Table, Materials and Methods, Figures, Data Availability Section) |
|---|---|---|
| **Plants:** provide species and strain, ecotype and cultivar where relevant, unique accession number if available, and source (including location for collected wild specimens). | Not Applicable | Not Applicable |
| **Microbes:** provide species and strain, unique accession number if available, and source. | Not Applicable | Not Applicable |

| Human research participants | Information included in the manuscript? | In which section is the information available? (Reagents and Tools Table, Materials and Methods, Figures, Data Availability Section) |
|---|---|---|
| If collected and within the bounds of privacy constraints report on age, sex and gender or ethnicity for all study participants. | Not Applicable | Not Applicable |

| Core facilities | Information included in the manuscript? | In which section is the information available? (Reagents and Tools Table, Materials and Methods, Figures, Data Availability Section) |
|---|---|---|
| If your work benefited from core facilities, was their service mentioned in the acknowledgments section? | Not Applicable | Not Applicable |

**Design**

| Study protocol | Information included in the manuscript? | In which section is the information available? (Reagents and Tools Table, Materials and Methods, Figures, Data Availability Section) |
|---|---|---|
| If study protocol has been **pre-registered, provide DOI in the manuscript**. For clinical trials, provide the trial registration number **OR** cite DOI. | Not Applicable | Not Applicable |
| Report the **clinical trial registration number** (at ClinicalTrials.gov or equivalent), where applicable. | Not Applicable | Not Applicable |

| Laboratory protocol | Information included in the manuscript? | In which section is the information available? (Reagents and Tools Table, Materials and Methods, Figures, Data Availability Section) |
|---|---|---|
| Provide DOI OR other citation details if **external detailed step-by-step protocols** are available. | Yes | Methods |

| Experimental study design and statistics | Information included in the manuscript? | In which section is the information available? (Reagents and Tools Table, Materials and Methods, Figures, Data Availability Section) |
|---|---|---|
| Include a statement about **sample size** estimate even if no statistical methods were used. | Yes | Figures |
| Were any steps taken to minimize the effects of subjective bias when allocating animals/samples to treatment (e.g. **randomization procedure**)? If yes, have they been described? | Yes | Methods -Selection of cell lines for follow ups AND Table EV3 |
| Include a statement about **blinding** even if no blinding was done. | Not Applicable | Not Applicatble |
| Describe **inclusion/exclusion criteria** if samples or animals were excluded from the analysis. Were the criteria pre-established?<br><br>If sample or data points were omitted from analysis, report if this was due to attrition or intentional exclusion and provide justification. | Yes | Methods -Selection of cell lines for follow ups AND Table EV3 |
| For every figure, are **statistical tests** justified as appropriate? Do the data meet the assumptions of the tests (e.g., normal distribution)? Describe any methods used to assess it. Is there an estimate of variation within each group of data? Is the variance similar between the groups that are being statistically compared? | Yes | Figures |

| Sample definition and in-laboratory replication | Information included in the manuscript? | In which section is the information available? (Reagents and Tools Table, Materials and Methods, Figures, Data Availability Section) |
|---|---|---|
| In the figure legends: state number of times the experiment was **replicated** in laboratory. | Yes | Figures |
| In the figure legends: define whether data describe **technical or biological replicates**. | Yes | Figures |

## Ethics

| Ethics | Information included in the manuscript? | In which section is the information available? (Reagents and Tools Table, Materials and Methods, Figures, Data Availability Section) |
|---|---|---|
| Studies involving **human participants**: State details of **authority granting ethics approval** (IRB or equivalent committee(s), provide reference number for approval. | Not Applicable | |
| Studies involving **human participants**: Include a statement confirming that **informed consent** was obtained from all subjects and that the experiments conformed to the principles set out in the WMA Declaration of Helsinki and the Department of Health and Human Services Belmont Report. | Not Applicable | |
| Studies involving **human participants:** For publication of **patient photos**, include a statement confirming that consent to publish was obtained. | Not Applicable | |
| Studies involving experimental **animals**: State details of **authority granting ethics approva**l (IRB or equivalent committee(s), provide reference number for approval. Include a statement of compliance with ethical regulations. | Not Applicable | |
| Studies involving **specimen and field samples:** State if relevant **permits** obtained, provide details of authority approving study; if none were required, explain why. | Not Applicable | |

| Dual Use Research of Concern (DURC) | Information included in the manuscript? | In which section is the information available? (Reagents and Tools Table, Materials and Methods, Figures, Data Availability Section) |
|---|---|---|
| Could your study fall under dual use research restrictions? Please check biosecurity documents and list of **select agents and toxins** (CDC): https://www.selectagents.gov/sat/list.htm | Not Applicable | |
| If you used a select agent, is the security level of the lab appropriate and reported in the manuscript? | Not Applicable | |
| If a study is subject to dual use research of concern regulations, is the name of the **authority granting approval and reference number** for the regulatory approval provided in the manuscript? | Not Applicable | |

## Reporting

The MDAR framework recommends adoption of discipline-specific guidelines, established and endorsed through community initiatives. Journals have their own policy about requiring specific guidelines and recommendations to complement MDAR.

| Adherence to community standards | Information included in the manuscript? | In which section is the information available? (Reagents and Tools Table, Materials and Methods, Figures, Data Availability Section) |
|---|---|---|
| State if relevant guidelines or checklists (e.g., **ICMJE, MIBBI, ARRIVE, PRISMA**) have been followed or provided. | Yes | We adhere to the reporting standards of the Metabolomics Standards Initiative (PMC3772505). Details in Methods. Data are available in mzTab format. |
| For **tumor marker prognostic studies,** we recommend that you follow the **REMARK** reporting guidelines (see link list at top right). See author guidelines, under 'Reporting Guidelines'. Please confirm you have followed these guidelines. | Not Applicable | Not applicable |
| For **phase II and III randomized controlled trials**, please refer to the **CONSORT** flow diagram (see link list at top right) and submit the CONSORT checklist (see link list at top right) with your submission. See author guidelines, under 'Reporting Guidelines'. Please confirm you have submitted this list. | Not Applicable | Not applicable |

## Data Availability

| Data availability | Information included in the manuscript? | In which section is the information available? (Reagents and Tools Table, Materials and Methods, Figures, Data Availability Section) |
|---|---|---|
| Have **primary datasets** been deposited according to the journal's guidelines (see 'Data Deposition' section) and the respective accession numbers provided in the Data Availability Section? | Yes | Data Availability Section |
| Were **human clinical and genomic datasets** deposited in a public access-controlled repository in accordance to ethical obligations to the patients and to the applicable consent agreement? | Not Applicable | |
| Are **computational models** that are central and integral to a study available without restrictions in a machine-readable form? Were the relevant accession numbers or links provided? | Yes | Data Availability Section (github) |
| If publicly available data were reused, provide the respective **data citations in the reference list**. | Yes | Methods |
